# Synthesis and Structure–Activity Analysis of Novel Potential Antifungal Cyclotryptamine Alkaloid Derivatives

**DOI:** 10.3390/molecules28062617

**Published:** 2023-03-13

**Authors:** Yazhou Yang, Yujie Zhou, Qiaoju Jiang, Yi Tan, Wenbin Wu, Ke Han, Rui Zhu, Shaojun Zheng

**Affiliations:** School of Environmental and Chemical Engineering, Jiangsu University of Science and Technology, Zhenjiang 212003, China; ads4cn@gmail.com (Y.Y.); zyj33662023@163.com (Y.Z.); 15136801650@163.com (Q.J.); tanyiris2020@163.com (Y.T.); b752526149@163.com (W.W.); h15289369325@163.com (K.H.); rzhu508@163.com (R.Z.)

**Keywords:** cyclotryptamine alkaloids, synthesis, biological activity, agrochemicals, SAR

## Abstract

A total of 39 novel cyclotryptamine alkaloid derivatives were prepared from 2-(1H-indol-3-yl) acetonitrile. The prepared compounds were evaluated against six plant pathogen fungi. Bioassay results revealed that most of the compounds displayed higher in vitro antifungal activities than the positive control. Notably, compound **b2** displayed the broadest and most effective activity among the tested cyclotryptamine alkaloid derivatives and might be a novel potential leading compound for further development as an antifungal agent.

## 1. Introduction

The abuse of chemical pesticides has had a serious impact on the environment and caused great harm to the survival of human beings and other organisms. Therefore, it is urgent to develop an efficient, low toxicity and environment-friendly pesticide [1,2].

**Figure 1:**
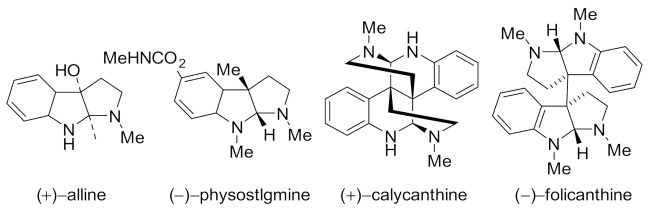
Structure of cyclotryptamine alkaloids.

Cyclotryptamine alkaloids (Figure 1), which can be isolated from plants, microorganisms and marine organisms, are a class of natural products with specific structural units of hexahydropyrrolo[2,3-b]indole [3]. The family is large and has a rich and important biological activity. One example is (-)-physistigmine, an alkaloid derived from the seeds of lentils grown in Africa, which now can be artificially synthesized and has an inhibitory effect on cholinesterase. It can shrink pupils and reduce intraocular pressure clinically. (-)-Folicanthine has very good biological activity. (-)-Win-64745 is a good neurokinin antagonist. (-)-Asperdimin has antiviral activity [4,5,6,7,8]. Cyclotryptamine alkaloids have unique structures and various medicinal values, which have attracted great interest among many synthetic chemists [8,9,10,11,12,13,14].

Research on the synthesis and activity of cyclotryptamine alkaloids mainly focuses more on the research and development of medicine, but less on the antifungal activity of agriculture. Pesticide research and pharmaceutical research have been learning from each other, and they have many similarities. Pesticide scientists have studied the agricultural antifungal activity of cyclotryptamine alkaloid compounds in recent years. The research shows that monomer cyclotryptamine alkaloids also have a strong inhibitory effect on many agricultural pathogenic fungi; *(+)-D calycanthine* has significant antibacterial activity against *Watermelon Fusarium Wilt* and other pathogens. Compound **c2** showed strong activity against acetylcholinesterase; the IC_50_ value was 0.01 ng mL^−1^ [15]. In addition, our group has reported the synthesis and biological profiling of a wide variety of half-cyclotryptamine alkaloid derivatives. It showed that changing substituents on heterocycles can affect biological activity [16,17,18].

Therefore, we put emphasis on the structural optimization of cyclotryptamine alkaloids. The aim is that compounds with better activity could be obtained. Herein, 39 cyclotryptamine alkaloid analogs were synthesized in good yield.

## 2. Results and Discussion

### 2.1. Synthesis of Cyclotryptamine Alkaloids

**Scheme 1:**
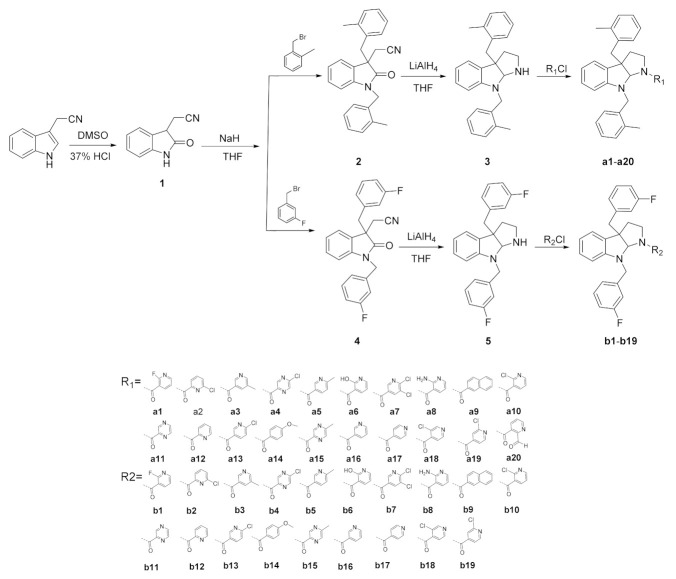
Synthesis route to the title compounds **a1**–**a20** and **b1–b19**.

Cyclotryptamine alkaloids were synthesized as depicted in Scheme 1. The target compounds were prepared starting from the inexpensive and readily obtainable 2-(1*H*-indol-3-yl) acetonitrile according to the route development in our group [19]. A total of 39 derivatives of cyclotryptamine alkaloids were prepared and characterized by ^1^H-NMR, ^13^C-NMR and ESI-MS. (^1^H and ^13^C NMR Spectra could be found in the Supplementary Materials).

### 2.2. Antifungal Acitivity

Antifungal activity tests of the target compounds are shown in Table 1. We use carbendazim and amphotericin B as positive controls, and MIC values were determined to evaluate the biological activities of cyclotryptamine alkaloids against *Sclerotinia sclerotiorum, Altenaria solani, Verticillium dahliae, Fusarium oxysporum, Walnut pythium and Curvularia lunata*.

It is observed that most of the compounds generally exhibited more effective antifungal activity than the positive controls. Compounds **b2**, **b4**, **b6**, **b10**, **b13** and **b15** showed significant antifungal activity against *Sclerotinia sclerotiorum*, of which **b2** and **b6** were the most effective compared with *carbendazim* and *amphotericin B*, with the same MIC values of 1.90 µg/mL. Compounds **b2**, **b4**, **b6**, **b10**, **b15** and **b17** revealed improved activity against *Altenaria solani* compared with the positive controls *carbendazim* and *amphotericin B*, with the same MIC value of 1.90 µg mL^−1^. Compounds **b2**, **b10** and **b17** manifested much more activity against *Verticillium dahliae* than *carbendazim*; **b10** and **b17** were the most effective, with a MIC value of 1.9 µg mL^−1^. The activity of compounds **b4**, **b5** and **b15** was more potent than *carbendazim* and *amphotericin B* against *Fusarium oxysporum,* all with the same MIC value of 3.90 µg mL^−1^. The activity of compound **b13** is more potent than *carbendazim* and *amphotericin B* against *Walnut pythium,* with a MIC value of 7.80 µg mL^−1^. Compounds **a2**, **a9**, **a11**, **a12**, **b3**, **b4**, **b8**, **b9**, **b11**, **b13**, **b16** and **b18** manifested much more activity against *Curvularia lunata* than carbendazim and amphotericin B, with MIC values between 7.8 and 31.3 µg mL^−1^. Compound **b4** in particular exhibited significant antifungal activity against *Curvularia lunata*, with a MIC value of 7.80 µg mL^−1^.

Although it is difficult to extract clear structure–activity relationships from the biological data, some conclusions can still be drawn. Firstly, when the position of two, five or six in the substituent is a Cl atom, such as in compounds **a13**, **b2**, **b4**, **b10** and **b13**, excellent antifungal effects are displayed. Secondly, when the position in the substituent is methyl group, such as in compounds **a3**, **a5**, **a15**, **b3**, **b5** and **b15**, an improved antifungal effect was shown. Thirdly, from compounds **a11**, **a12, b11 and b12**, it can be seen that when the compound contains pyrazine, the increase of the number of N atoms in the compound has a promoting effect on the antibacterial activity. Finally, as far as the parent structure is concerned, when the compound contains 3-F benzyl bromide, it is less active than compounds containing methyl bromide.

## 3. Materials and Methods

### 3.1. Instruments and Chemicals

All solvents and substances used were commercially purchased and not further purified. The reactions were monitored by thin-layer chromatography (TLC) with silica gel plates using silica gel 60 GF_254_ (Qingdao Haiyang Chemical Co., Ltd., Qingdao, China). ^1^H−NMR (400 MHz) and ^13^C−NMR (100 MHz) were measured by an AM−500 FT−NMR spectrometer (Bruker Corporation, Fällanden, Switzerland) with CDCl_3_, acetone-*d_6_* or DMSO-*d_6_* as the solvent and TMS as the internal standard. MS was recorded under ESI conditions using LCQ Fleet instruments (Thermo Fisher, Waltham, MA, USA). The yields of the reactions were measured before optimization.

### 3.2. Synthesis

The general synthesis method of compounds **a1**–**a20** and **b1**–**b19** is shown in Scheme 1.

#### 3.2.1. General Procedures for the Synthesis of Target Compound **1**

Concentrated hydrochloric acid (37%, 100 mL) was added slowly to a stirred solution of 3-Indoleacetonitrile (3.12 g, 20 mmol) in dimethyl sulfoxide (25 mL) at 0 °C. The resulting mixture was allowed to warm to room temperature for 1 h. The solvents were removed to obtain crude compound **1** (3.20 g) with a yield of 93%.

#### 3.2.2. General Procedures for the Synthesis of Target Compound **2**

Under a N_2_ atmosphere, NaH (1.8 g, 75 mmol) was added portionwise to a stirred solution of compound **1** (2.58 g, 15 mmol) in dry THF through an injector. 2-methylbenzyl bromide (6.45 g, 37.5 mmol) was added dropwise into the flask in an ice bath. The resulting solution was stirred for 11 h at room temperature. Then, it was quenched with ammonium chloride (3 mL) and stirred for 10 min. The reaction mixture was continuously extracted with acetic ether (3 × 50 mL), and the combined organic layer was washed with saturated salt water. The organic extracts were combined, washed with brine, dried over Na_2_SO_4_ and concentrated. The residue was purified by flash column chromatography (V_PE_: V_EA_ = 6:1) to obtain compound **2** (4.28 g, 75%).

#### 3.2.3. General Procedures for the Synthesis of Target Compound **3**

LiAlH_4_ (60 mmol, 7.5 eq) was added to compound **2** (3.52 g, 10 mmol) in anhydrous THF (25 mL) at −78 °C by a low temperature reactor under a nitrogen and anhydrous atmosphere. After stirring at room temperature for 1 h, the reaction mixture was heated to 75 °C for 4 h. When TLC monitoring indicated that the starting material, compound **2,** had disappeared, the mixture was cooled to room temperature and quenched by acetic ether and water dropwise in an ice bath. The resulting solution was filtered to remove the deposition, and the filtrate was extracted with acetic ether (3 × 50 mL). The combined organic layer was washed with saturate salt water and dried over Na_2_SO_4_. The residue was purified by flash column chromatography (V_PE_:V_EA_:V_Et3N_ = 100:50:2) to obtain compound **3** (2.50 g, 68%).

#### 3.2.4. General Procedures for the Synthesis of Target Compound **4**

Under a N_2_ atmosphere, NaH (1.8 g, 75 mmol) was added portionwise to a stirred solution of compound **1** (2.58 g, 15 mmol) in dry THF through an injector. 3-fluorobenzyl bromide (7.05 g, 37.5 mmol) was added dropwise into the flask in an ice bath. The resulting solution was stirred for 11 h at room temperature. Then, it was quenched with ammonium chloride (3 mL) and stirred for 10 min. The reaction mixture was continuously extracted with acetic ether (3 × 50 mL), and the combined organic layer was washed with saturated salt water. The organic extracts were combined, washed with brine, dried over Na_2_SO_4_ and concentrated. The residue was purified by flash column chromatography (silica gel, 12.5% petroleum ether/acetic ether) to obtain compound **4** (4.13 g, 71%).

#### 3.2.5. General Procedures for the Synthesis of Target Compound **5**

LiAlH_4_ (60 mmol, 7.5 eq) was added to compound **4** (3.88 g, 10 mmol) in anhydrous THF (25 mL) at −78 °C by a low temperature reactor under a nitrogen and anhydrous atmosphere. After stirring at room temperature for 1 h, the reaction mixture was heated to 75 °C for 4 h. When TLC monitoring indicated that the starting material, compound **4,** had disappeared, the mixture was cooled to room temperature and quenched by acetic ether and water dropwise in an ice bath. The resulting solution was filtered to remove the deposition, and the filtrate was extracted with acetic ether (3 × 50 mL). The combined organic layer was washed with saturate salt water and dried over Na_2_SO_4_. The residue was purified by flash column chromatography (V_PE_:V_EA_:V_Et3N_ = 100:50:2) to obtain compound **5** (2.74 g, 73%).

#### 3.2.6. General Procedure for the Synthesis of **a1**–**a20** and **b1**–**b19**

The corresponding desired reagent (2.5 eq) was dissolved in dry CH_2_Cl_2_ (10 mL). Then, sulfoxide chloride (4 eq) was added to the solution, and the solution was refluxed at 55 °C for 2 h. After the solvent had been evaporated under reduced pressure, the reagent of sulfonyl chlorination was afforded.

To a stirred solution of compound **3** or **5** in anhydrous CH_2_Cl_2_ (10 mL) was added Et_3_N (1.5 eq) and the corresponding desired reagent of sulfonyl chlorination successively in an ice bath. The reaction mixture was transferred to room temperature and stirred for 2 h (monitored by TLC). The resulting solution was quenched by NaHCO_3_ (3 mL) and then extracted with dichloromethane (3 × 30 mL), washed with saturate salt water and dried over anhydrous Na_2_SO_4_. After the removal of the solvent, the residue was purified by flash chromatography using a mixture of acetic ether and petroleum ether as an eluent to afford the target products **a1**–**a20** and **b1**–**b19**.

(3a,8-bis(2-methylbenzyl)-3,3a,8,8a-tetrahydropyrrolo[2,3-b]indol-1(2H)-yl)(2-fluoropyridin-3-yl) methanone(**a1**): Yield: 89%. White solid. Melting point: 128–130 °C. R_f_ = 0.24 (PE/EA 4:1). ^1^H-NMR (400 MHz, Acetone-*d*_6_) δ 8.30–8.27 (m, 1H), 7.85 (ddd, *J* = 9.3, 7.4, 2.0 Hz, 1H), 7.38 (ddd, *J* = 7.2, 4.9, 2.1 Hz, 1H), 7.16–7.12 (m, 3H), 7.11 (d, *J* = 2.0 Hz, 1H), 7.08 (dd, *J* = 4.2, 2.7 Hz, 2H), 7.07–7.02 (m, 2H), 7.00 (d, *J* = 7.8, 1.2 Hz, 2H), 6.76–6.72 (m, 1H), 6.59 (t, *J* = 7.4 Hz, 1H), 6.22 (d, *J* = 7.9 Hz, 1H), 5.95 (s, 1H), 4.77 (d, *J* = 17.0 Hz, 1H), 4.60 (d, *J* = 16.9 Hz, 1H), 3.47 (d, *J* = 10.8, 6.4 Hz, 1H), 3.31–3.23 (m, 2H), 3.11 (d, *J* = 13.7 Hz, 1H), 2.37–2.26 (m, 4H), 2.01 (s, 3H). ^13^C-NMR (100 MHz Acetone-*d*_6_) δ 164.04 (d, *J* = 4.7 Hz), 158.82 (d, *J* = 237.0 Hz), 150.71, 149.03 (d, *J* = 14.9 Hz), 140.53, 140.51, 137.28, 137.00, 136.08, 135.32, 131.40, 130.40 (d, *J* = 3.7 Hz), 130.02, 128.55, 126.69, 126.38, 125.76, 125.61, 123.47, 122.04 (d, *J* = 4.1 Hz), 119.61 (d, *J* = 33.3 Hz), 117.35, 106.01, 83.23, 57.32, 48.21, 47.28, 39.87, 37.24, 19.33, 18.56. MS (ESI(+)) calcd for C_32_H_30_FN_3_O^+^ [M + H]^+^: 492.2; found: 492.2.

(3a,8-bis(2-methylbenzyl)-3,3a,8,8a-tetrahydropyrrolo[2,3-b]indol-1(2H)-yl)(6-chloropyridin-2-yl) methanone(**a2**): Yield: 84%. White solid. Melting point: 140–142 °C. R_f_ = 0.23 (PE/EA 1:1). ^1^H NMR (400 MHz, Acetone-*d*_6_) δ 8.65 (s, 2H), 7.08 (ddd, *J* = 21.8, 14.8, 5.8 Hz, 10H), 6.60 (t, *J* = 7.4 Hz, 1H), 6.24 (d, *J* = 7.8 Hz, 1H), 5.98 (s, 1H), 4.80 (d, *J* = 16.8 Hz, 1H), 4.59 (d, *J* = 16.8 Hz, 1H), 3.91 (dd, *J* = 11.5, 6.9 Hz, 1H), 3.37–3.21 (m, 2H), 3.05 (d, *J* = 13.7 Hz, 1H), 2.31 (s, 3H), 2.22 (s, 1H), 2.09 (s, 2H), 2.01 (s, 3H). ^13^C NMR (100 MHz, Acetone-*d*_6_) δ 165.99, 154.50, 150.53, 149.23, 140.27, 137.28, 137.00, 136.06, 135.31, 131.60, 130.51, 130.31, 129.96, 129.52, 128.43, 126.62, 126.32, 125.72, 125.67, 125.55, 123.33, 122.81, 117.26, 106.12, 83.52, 56.70, 49.21, 47.14, 39.64, 36.85, 19.23, 18.57. MS (ESI(+)) calcd for C_32_H_30_CN_3_O^+^ [M + H]^+^: 508.2; found: 508.3.

(3a,8-bis(2-methylbenzyl)-3,3a,8,8a-tetrahydropyrrolo[2,3-b]indol-1(2H)-yl)(5-methylpyridin-3-yl) methanone(**a3**): Yield: 91%. White solid. Melting point: 162–164 °C. R_f_ = 0.23 (PE/EA 4:1). ^1^H NMR (400 MHz, Acetone-*d*_6_) δ 8.43 (d, *J* = 16.2 Hz, 2H), 7.58 (s, 1H), 7.26–6.93 (m, 10H), 6.72 (d, *J* = 7.2 Hz, 1H), 6.58 (t, *J* = 7.3 Hz, 1H), 6.22 (d, *J* = 7.8 Hz, 1H), 6.03 (s, 1H), 4.76 (d, *J* = 16.9 Hz, 1H), 4.59 (d, *J* = 17.0 Hz, 1H), 3.64–2.83 (m, 6H), 2.31 (d, *J* = 13.8 Hz, 8H). ^13^C NMR (100 MHz, Acetone-*d*_6_) δ 168.02, 151.38, 150.68, 145.63, 137.29, 137.09, 136.11, 135.28, 135.16, 132.73, 131.71, 131.45, 130.41, 130.32, 129.98, 128.47, 126.63, 126.30, 125.69, 125.56, 123.41, 117.12, 105.87, 83.13, 70.34, 56.91, 49.52, 46.96, 39.78, 37.22, 19.26, 18.51, 17.24. MS (ESI(+)) calcd for C_33_H_33_N_2_O^+^ [M + H]^+^: 488.2; found: 488.2.

(3a,8-bis(2-methylbenzyl)-3,3a,8,8a-tetrahydropyrrolo[2,3-b]indol-1(2H)-yl)(5-chloropyrazin-2-yl) methanone(**a4**): Yield: 84%. Light yellow solid. Melting point: 134–136 °C. R_f_ = 0.23 (PE/EA 8:1). ^1^H NMR (400 MHz, Acetone-*d*_6_) δ 8.65 (s, 1H), 8.20 (d, *J* = 35.3 Hz, 1H), 7.16–6.89 (m, 12H), 6.76 (d, *J* = 7.3 Hz, 1H), 6.60 (t, *J* = 7.4 Hz, 1H), 6.24 (d, *J* = 7.8 Hz, 1H), 5.98 (s, 1H), 4.80 (d, *J* = 16.8 Hz, 1H), 4.59 (d, *J* = 16.8 Hz, 1H), 3.91 (dd, *J* = 11.5, 6.9 Hz, 1H), 3.47–3.21 (m, 3H), 3.05 (d, *J* = 13.7 Hz, 1H), 2.31 (s, 3H), 1.88 (s, 1H). ^13^C NMR (100 MHz, Acetone-*d*_6_) δ 164.85, 147.71, 144.99, 142.38, 137.27, 136.95, 135.99, 135.33, 130.51, 130.33, 129.99, 128.46, 126.65, 126.55, 126.36, 125.69, 125.56, 123.32, 117.36, 106.15, 83.53, 56.73, 49.06, 47.22, 39.60, 36.78, 31.42, 22.40, 19.21, 18.54, 13.47. MS (ESI(+)) calcd for C_31_H_29_ClN_4_O^+^ [M + H]^+^: 509.2; found: 509.2.

(3a,8-bis(2-methylbenzyl)-3,3a,8,8a-tetrahydropyrrolo[2,3-b]indol-1(2H)-yl)(6-methylpyridin-3-yl) methanone(**a5**): Yield: 89%. White solid. Melting point: 187–189 °C. R_f_ = 0.32 (PE/EA 8:1). ^1^H NMR (400 MHz, DMSO-*d*_6_) δ 8.45 (s, 1H), 7.71 (dd, *J* = 8.0, 2.1 Hz, 1H), 7.26 (d, *J* = 8.0 Hz, 1H), 7.13–6.96 (m, 9H), 6.71 (d, *J* = 7.1 Hz, 1H), 6.56 (d, *J* = 7.3 Hz, 1H), 6.17 (d, *J* = 7.8 Hz, 1H), 5.90 (s, 1H), 4.57 (d, *J* = 8.6 Hz, 2H), 3.54–3.44 (m, 1H), 3.13 (d, *J* = 13.6 Hz, 1H), 3.02 (d, *J* = 13.6 Hz, 1H), 2.47 (s, 3H), 2.24–2.19 (m, 4H), 2.09 (d, *J* = 1.1 Hz, 2H), 1.93 (s, 3H). ^13^C NMR (100 MHz, DMSO-*d*_6_) δ 168.12, 160.39, 150.58, 148.17, 137.45, 137.16, 136.36, 136.06, 135.50, 131.68, 130.70, 130.42, 129.22, 128.91, 127.08, 126.73, 126.29, 126.13, 126.02, 123.83, 122.99, 117.43, 106.10, 83.09, 56.96, 49.82, 47.20, 37.54, 31.26, 24.53, 20.07, 19.37. MS (ESI(+)) calcd for C_33_H_33_N_3_O^+^ [M + H]^+^: 488.3; found: 488.3.

(3a,8-bis(2-methylbenzyl)-3,3a,8,8a-tetrahydropyrrolo[2,3-b]indol-1(2H)-yl)(2-hydroxypyridin-3-yl) methanone(**a6**): Yield: 90%. White solid. Melting point: 248–250 °C. R_f_ = 0.25 (PE/EA 4:1). ^1^H NMR (400 MHz, DMSO-*d*_6_) δ 12.41 (s, 1H), 9.79 (t, *J* = 5.8 Hz, 1H), 8.26 (dd, *J* = 7.2, 2.1 Hz, 1H), 7.75–7.55 (m, 2H), 7.24–7.17 (m, 1H), 7.09 (d, *J* = 7.5 Hz, 2H), 7.04–6.98 (m, 4H), 6.92 (t, *J* = 7.2 Hz, 1H), 6.85 (t, *J* = 7.4 Hz, 1H), 6.58 (d, *J* = 7.5 Hz, 1H), 6.43–6.37 (m, 1H), 5.95 (d, *J* = 7.6 Hz, 1H), 5.01 (s, 2H), 3.87 (s, 2H), 3.50 (dd, *J* = 13.1, 6.8 Hz, 2H), 2.93 (t, *J* = 7.1 Hz, 2H), 2.13 (s, 3H), 2.09 (s, 3H). ^13^C NMR (100 MHz, DMSO-*d*_6_) δ 163.80, 162.68, 144.22, 139.71, 137.45, 137.09, 136.76, 136.07, 135.63, 134.93, 130.29, 127.95, 127.51, 127.12, 126.66, 126.62, 126.55, 124.82, 121.47, 120.96, 119.40, 118.82, 110.98, 110.15, 106.65, 44.49, 27.59, 24.90, 19.61, 19.06. MS (ESI(+)) calcd for C_32_H_31_N_3_O_2_^+^ [M + H]^+^: 490.3; found: 490.3.

(3a,8-bis(2-methylbenzyl)-3,3a,8,8a-tetrahydropyrrolo[2,3-b]indol-1(2H)-yl)(5,6-dichloropyridin-3-yl) methanone(**a7**): Yield: 83%. White solid. Melting point: 162–164 °C. R_f_ = 0.34 (PE/EA 8:1). ^1^H NMR (400 MHz, Acetone-*d*_6_) δ 8.33 (d, *J* = 1.8 Hz, 1H), 7.95 (d, *J* = 1.8 Hz, 1H), 7.04 (d, *J* = 16.6, Hz, 9H), 6.71 (d, *J* = 7.2 Hz, 1H), 6.56 (t, *J* = 7.4 Hz, 1H), 6.21 (d, *J* = 7.8 Hz, 1H), 5.97 (s, 1H), 4.70 (d, *J* = 17.0 Hz, 1H), 4.57 (d, *J* = 17.0 Hz, 1H), 3.73–3.58 (m, 1H), 3.42 (td, *J* = 10.5, 6.8 Hz, 1H), 3.23 (d, *J* = 13.7 Hz, 1H), 3.05 (d, *J* = 13.7 Hz, 1H), 2.33–2.23 (m, 5H), 1.98 (s, 3H). ^13^C NMR (100 MHz, Acetone-*d*_6_) δ 165.35, 150.65, 149.38, 146.35, 138.09, 137.31, 137.11, 136.05, 135.36, 132.92, 131.39, 130.44, 130.05, 129.65, 128.56, 126.70, 126.39, 125.71, 123.46, 117.28, 105.97, 83.44, 57.05, 49.42, 47.06, 39.89, 37.32, 31.50, 22.50, 19.32, 18.56, 13.57. MS (ESI(+)) calcd for C_32_H_29_Cl_2_N_3_O^+^ [M + H]^+^: 543.2; found: 543.2.

(2-aminopyridin-3-yl)(3a,8-bis(2-methylbenzyl)-3,3a,8,8a-tetrahydropyrrolo[2,3-b]indol-1(2H)-yl) methanone(**a8**): Yield: 89%. White solid. Melting point: 157–159 °C. R_f_ = 0.24 (PE/EA 3:1). ^1^H NMR (400 MHz, DMSO-*d*_6_) δ 7.97 (d, *J* = 4.0 Hz, 1H), 7.30 (s, 1H), 7.15–6.91 (m, 9H), 6.64 (d, *J* = 7.1 Hz, 1H), 6.52 (t, *J* = 7.4 Hz, 1H), 6.11 (s, 3H), 4.52 (s, 1H), 3.14 (d, *J* = 13.6 Hz, 1H), 3.05 (d, *J* = 13.6 Hz, 1H), 2.17 (dd, *J* = 16.9, 11.7 Hz, 5H), 2.07 (s, 5H), 1.90 (s, 3H). ^13^C NMR (100 MHz, DMSO-*d*_6_) δ 157.02, 150.26, 149.87, 137.01, 136.74, 136.02, 135.03, 131.17, 130.22, 130.16, 129.98, 128.42, 126.59, 126.28, 125.69, 125.54, 123.43, 116.95, 112.94, 112.67, 111.46, 105.57, 104.71, 89.84, 82.62, 46.69, 37.36, 30.80, 19.62, 18.88, 14.09. MS (ESI(+)) calcd for C_32_H_32_N_4_O^+^ [M + H]^+^: 489.2; found: 489.2.

(3a,8-bis(2-methylbenzyl)-3,3a,8,8a-tetrahydropyrrolo[2,3-b]indol-1(2H)-yl)(naphthalen-2-yl) methanone(**a9**): Yield: 91%. White solid. Melting point: 177–179 °C. R_f_ = 0.21 (PE/EA 10:1). ^1^H NMR (400 MHz, Acetone-*d*_6_) δ 7.91 (d, *J* = 8.2 Hz, 2H), 7.59–7.37 (m, 4H), 7.29–7.09 (m, 9H), 7.03 (t, *J* = 7.7 Hz, 1H), 6.82 (d, *J* = 7.3 Hz, 1H), 6.59 (t, *J* = 7.4 Hz, 1H), 6.30–6.15 (m, 2H), 4.89 (d, *J* = 17.0 Hz, 1H), 4.78 (d, *J* = 17.0 Hz, 1H), 3.32 (d, *J* = 13.7 Hz, 1H), 3.21–2.98 (m, 3H), 2.36 (s, 3H), 2.27–2.07 (m, 5H). ^13^C NMR (100 MHz, Acetone-*d*_6_) δ 169.23, 150.83, 137.32, 136.16, 135.38, 135.25, 133.46, 131.92, 130.47, 130.42, 130.05, 129.34, 128.97, 128.47, 128.31, 126.89, 126.68, 126.37, 126.27, 126.24, 125.77, 125.65, 125.11, 124.79, 123.85, 123.37, 117.19, 105.67, 57.29, 48.22, 39.76, 37.45, 19.39, 18.51. MS (ESI(+)) calcd for C_37_H_34_N_2_O^+^ [M + H]^+^: 523.3; found: 523.3.

(3a,8-bis(2-methylbenzyl)-3,3a,8,8a-tetrahydropyrrolo[2,3-b]indol-1(2H)-yl)(2-chloropyridin-3-yl) methanone(**a10**): Yield: 78%. White solid. Melting point: 123–125 °C. R_f_ = 0.24 (PE/EA 2:1). ^1^H NMR (400 MHz, Acetone-*d*_6_) δ 8.40 (dd, *J* = 4.6, 1.8 Hz, 1H), 7.40 (s, 1H), 7.16–6.97 (m, 10H), 6.79 (d, *J* = 7.2 Hz, 1H), 6.58 (t, *J* = 7.4 Hz, 1H), 6.20 (d, *J* = 7.9 Hz, 1H), 5.94 (s, 1H), 4.80 (d, *J* = 16.9 Hz, 1H), 4.61 (s, 1H), 3.33–3.23 (m, 2H), 3.13 (d, *J* = 13.8 Hz, 2H), 2.36–2.22 (m, 5H), 2.05 (d, *J* = 3.4 Hz, 3H). ^13^C NMR (100 MHz, Acetone-*d*_6_) δ 166.46, 151.03, 150.90, 150.55, 147.25, 137.42, 137.21, 136.30, 135.56, 131.56, 130.73, 130.68, 130.46, 128.95, 127.11, 126.77, 126.27, 126.15, 126.05, 123.85, 122.46, 121.34, 117.53, 106.14, 83.22, 57.13, 49.33, 47.17, 37.57, 20.09, 19.39. C_32_H_30_ClN_3_O^+^ [M + H]^+^: 508.2; found: 508.2.

(3a,8-bis(2-methylbenzyl)-3,3a,8,8a-tetrahydropyrrolo[2,3-b]indol-1(2H)-yl)(pyrazin-2-yl) methanone (**a11**): Yield: 83%. Light yellow solid. Melting point: 140–142 °C. R_f_ = 0.23 (PE/EA 4:1). ^1^H NMR (400 MHz, DMSO-*d*_6_) δ 8.55–8.52 (m, 1H), 7.89 (td, *J* = 7.7, 1.7 Hz, 1H), 7.61–7.58 (m, 1H), 7.46 (d, *J* = 7.6, 4.8, 1.1 Hz, 1H), 7.12–7.06 (m, 5H), 7.00–6.93 (m, 5H), 6.76–6.71 (m, 1H), 6.56 (t, *J* = 7.3 Hz, 1H), 6.18 (d, *J* = 7.8 Hz, 1H), 5.84 (s, 1H), 4.66 (d, *J* = 16.8 Hz, 1H), 4.51 (d, *J* = 16.8 Hz, 1H), 3.11 (d, *J* = 13.6 Hz, 1H), 2.99 (d, *J* = 13.6 Hz, 1H), 2.23 (s, 3H), 2.08 (s, 1H), 1.94 (s, 3H), 1.81 (s, 1H). ^13^C NMR (100 MHz, DMSO-*d*_6_) δ 167.97, 154.11, 150.55, 148.67, 147.44, 137.75, 137.42, 137.17, 136.34, 135.62, 131.93, 130.72, 130.45, 128.84, 127.07, 126.81, 126.64, 126.03, 125.75, 124.62, 124.05, 123.75, 117.57, 106.28, 83.13, 56.84, 49.46, 47.19, 37.29, 20.05, 19.44. MS (ESI(+)) calcd for C_31_H_30_N_4_O^+^ [M + H]^+^: 475.2; found: 475.2.

(3a,8-bis(2-methylbenzyl)-3,3a,8,8a-tetrahydropyrrolo[2,3-b]indol-1(2H)-yl)(pyridin-2-yl) methanone(**a12**): Yield: 87%. White solid. Melting point: 182–184 °C. R_f_ = 0.23 (PE/EA 6:1). ^1^H NMR (400 MHz, DMSO-*d*_6_) δ 8.57–8.51 (m, 1H), 7.89 (ddd, *J* = 7.8, 1.7, 0.8 Hz, 1H), 7.60 (d, *J* = 7.8 Hz, 1H), 7.51–7.45 (m, 1H), 7.14–7.07 (m, 5H), 7.00–6.94 (m, 5H), 6.74 (d, *J* = 7.2 Hz, 1H), 6.56 (t, *J* = 7.4 Hz, 1H), 6.18 (d, *J* = 7.8 Hz, 1H), 5.85 (s, 1H), 4.67 (d, *J* = 16.8 Hz, 1H), 4.51 (d, *J* = 16.8 Hz, 1H), 3.75–3.64 (m, 1H), 3.12 (d, *J* = 13.6 Hz, 1H), 3.00 (d, *J* = 13.5 Hz, 1H), 2.24 (s, 3H), 2.10–2.08 (m, 1H), 1.94 (s, 3H), 1.82 (s, 1H). ^13^C NMR (100 MHz, DMSO-*d*_6_) δ 167.97, 154.12, 150.55, 148.68, 147.44, 137.75, 137.42, 137.17, 136.34, 135.62, 131.93, 130.72, 130.45, 128.84, 127.07, 126.80, 126.65, 126.18, 126.02, 125.75, 124.63, 124.05, 123.75, 117.57, 106.28, 83.14, 56.84, 49.45, 47.19, 37.30, 20.04, 19.44. MS (ESI(+)) calcd for C_32_H_31_N_3_O^+^ [M + H]^+^: 474.2; found: 474.2.

(3a,8-bis(2-methylbenzyl)-3,3a,8,8a-tetrahydropyrrolo[2,3-b]indol-1(2H)-yl)(6-chloropyridin-3-yl) methanone(**a13**): Yield: 84%. White solid. Melting point: 180–182 °C. R_f_ = 0.31 (PE/EA 6:1). ^1^H NMR (400 MHz, DMSO-*d*_6_) δ 8.39 (s, 1H), 7.86 (d, *J* = 6.6 Hz, 1H), 7.50 (d, *J* = 7.2 Hz, 1H), 7.12–6.92 (m, 9H), 6.70 (d, *J* = 6.1 Hz, 1H), 6.53 (s, 1H), 6.16 (d, *J* = 6.0 Hz, 1H), 5.84 (s, 1H), 4.54 (s, 2H), 3.10 (d, *J* = 13.1 Hz, 1H), 2.99 (d, *J* = 12.7 Hz, 1H), 2.20 (s, 4H), 2.06 (s, 4H), 1.91 (s, 2H). ^13^C NMR (100 MHz, Chloroform-*d*) δ 167.02, 153.08, 150.32, 148.64, 138.18, 137.57, 136.73, 135.61, 135.57, 130.76, 130.71, 130.66, 130.40, 128.95, 126.95, 126.67, 126.14, 125.84, 125.75, 124.07, 123.55, 117.54, 106.41, 83.91, 57.03, 49.89, 47.51, 40.17, 37.15, 31.09, 20.01, 19.49. MS (ESI(+)) calcd for C_32_H_30_ClN_3_O^+^ [M + H]^+^: 508.2; found: 508.2.

(3a,8-bis(2-methylbenzyl)-3,3a,8,8a-tetrahydropyrrolo[2,3-b]indol-1(2H)-yl)(4-methoxyphenyl) methanone(**a14**): Yield: 90%. White solid. Melting point: 144–146 °C. R_f_ = 0.26 (PE/EA 6:1). ^1^H NMR (400 MHz, Acetone-*d*_6_) δ 7.45 (d, *J* = 5.6 Hz, 2H), 7.18–7.01 (m, 9H), 6.91 (d, *J* = 7.2 Hz, 2H), 6.60 (dd, *J* = 31.2, 24.0 Hz, 2H), 6.22–5.97 (m, 2H), 4.77 (d, *J* = 16.4 Hz, 1H), 4.52 (d, *J* = 16.3 Hz, 1H), 3.81 (s, 3H), 3.62 (d, *J* = 25.1 Hz, 1H), 3.30 (d, *J* = 13.6 Hz, 1H), 3.06 (d, *J* = 13.6 Hz, 1H), 2.27 (s, 5H), 2.07–2.03 (m, 4H). ^13^C NMR (100 MHz, Acetone-*d*_6_) δ 169.76, 161.28, 150.78, 137.29, 137.03, 136.20, 135.24, 131.59, 130.46, 130.30, 129.92, 129.70, 128.47, 128.39, 126.59, 126.42, 126.28, 125.71, 125.54, 123.36, 117.04, 113.17, 105.89, 83.00, 70.34, 56.76, 54.83, 49.99, 46.86, 39.80, 37.14, 19.25, 18.51. MS (ESI(+)) calcd for C_34_H_34_N_2_O_2_^+^ [M + H]^+^: 502.2; found: 502.2.

(3a,8-bis(2-methylbenzyl)-3,3a,8,8a-tetrahydropyrrolo[2,3-b]indol-1(2H)-yl)(5-methylpyrazin-2-yl) methanone(**a15**): Yield: 82%. Light yellow solid. Melting point: 116–118 °C. R_f_ = 0.21 (PE/EA 5:1). ^1^H NMR (400 MHz, Acetone-*d*_6_) δ 8.72 (s, 1H), 8.46 (s, 1H), 7.08 (ddd, *J* = 25.4, 12.0, 6.1 Hz, 10H), 6.74 (t, *J* = 6.6 Hz, 1H), 6.60 (dd, *J* = 13.2, 5.7 Hz, 1H), 6.23 (d, *J* = 7.8 Hz, 1H), 5.99 (s, 1H), 4.82 (d, *J* = 16.8 Hz, 1H), 4.58 (d, *J* = 16.8 Hz, 1H), 3.94–3.85 (m, 1H), 3.59 (s, 1H), 3.25 (dd, *J* = 13.8, 4.8 Hz, 1H), 3.05 (d, *J* = 13.6 Hz, 1H), 2.55 (s, 3H), 2.36 (s, 1H), 2.30 (d, *J* = 6.2 Hz, 4H), 2.23 (s, 1H), 1.84 (s, 1H). ^13^C NMR (100 MHz, Acetone-*d*_6_) δ 166.05, 155.36, 150.55, 144.38, 142.07, 140.46, 137.28, 137.01, 136.07, 135.32, 131.64, 130.51, 130.32, 129.97, 128.42, 126.63, 126.54, 126.33, 125.71, 125.56, 123.32, 117.25, 106.08, 83.42, 56.65, 49.11, 47.16, 39.61, 36.87, 20.76, 19.25, 18.58. MS (ESI(+)) calcd for C_32_H_32_N_4_O^+^ [M + H]^+^: 489.2; found: 489.2.

(3a,8-bis(2-methylbenzyl)-3,3a,8,8a-tetrahydropyrrolo[2,3-b]indol-1(2H)-yl)(pyridin-3-yl) methanone(**a16**): Yield: 87%. White solid. Melting point: 159–161 °C. R_f_ = 0.23 (PE/EA 3:1). ^1^H NMR (400 MHz, Acetone-*d*_6_) δ 8.60 (s, 2H), 7.79 (d, *J* = 7.6 Hz, 1H), 7.43–7.31 (m, 1H), 7.23–6.91 (m, 10H), 6.72 (d, *J* = 7.2 Hz, 1H), 6.57 (t, *J* = 7.3 Hz, 1H), 6.21 (d, *J* = 7.8 Hz, 1H), 6.03 (s, 1H), 4.75 (d, *J* = 16.9 Hz, 1H), 4.59 (d, *J* = 17.0 Hz, 1H), 3.64–3.54 (m, 1H), 3.40 (dd, *J* = 17.3, 10.4 Hz, 1H), 3.26 (d, *J* = 13.6 Hz, 1H), 3.08 (d, *J* = 13.6 Hz, 1H), 2.38–2.19 (m, 5H), 2.00 (s, 2H). ^13^C NMR (100 MHz, Acetone-*d*_6_) δ 167.87, 150.96, 150.69, 148.49, 137.28, 137.06, 136.10, 135.27, 134.91, 132.21, 131.46, 130.41, 130.31, 129.97, 128.46, 126.62, 126.29, 125.68, 125.54, 123.40, 123.00, 117.15, 105.91, 83.24, 56.92, 49.50, 47.03, 39.80, 37.26, 19.23, 18.47. MS (ESI(+)) calcd for C_32_H_31_N_3_O^+^ [M + H]^+^: 474.2; found: 474.2.

(3a,8-bis(2-methylbenzyl)-3,3a,8,8a-tetrahydropyrrolo[2,3-b]indol-1(2H)-yl)(pyridin-4-yl) methanone(**a17**): Yield: 90%. White solid. Melting point: 144–146 °C. R_f_ = 0.24 (PE/EA 3:1). ^1^H NMR (400 MHz, DMSO-*d*_6_) δ 8.63 (d, *J* = 4.4 Hz, 2H), 7.22 (d, *J* = 5.9 Hz, 2H), 7.17–7.03 (m, 9H), 6.64–6.55 (m, 2H), 6.27 (d, *J* = 7.9 Hz, 1H), 5.97 (s, 1H), 4.80 (d, *J* = 16.9 Hz, 1H), 4.63 (d, *J* = 16.9 Hz, 1H), 3.45–3.29 (m, 2H), 3.22 (d, *J* = 13.7 Hz, 1H), 3.00 (d, *J* = 13.7 Hz, 1H), 2.33 (s, 3H), 2.25–2.14 (m, 2H), 1.97 (s, 3H). ^13^C NMR (100 MHz, DMSO-*d*_6_) δ 168.16, 150.35, 149.95, 143.80, 137.57, 136.79, 135.66, 135.59, 130.80, 130.66, 130.40, 128.93, 126.95, 126.66, 126.23, 125.85, 125.77, 123.54, 121.56, 117.53, 106.42, 83.84, 57.13, 49.62, 47.53, 40.16, 37.10, 20.02, 19.51. MS (ESI(+)) calcd for C_32_H_31_N_3_O^+^ [M + H]^+^: 474.2; found: 474.2.

(3a,8-bis(2-methylbenzyl)-3,3a,8,8a-tetrahydropyrrolo[2,3-b]indol-1(2H)-yl)(3-chloropyridin-4-yl) methanone(**a18**): Yield: 91%. Yellow oily liquid. R_f_ = 0.24 (PE/EA 3:1). ^1^H NMR (400 MHz, Acetone-*d*_6_) δ 8.65 (s, 1H), 8.55 (d, *J* = 4.5 Hz, 1H), 7.30–6.95 (m, 11H), 6.84 (d, *J* = 7.3 Hz, 1H), 6.63 (t, *J* = 7.4 Hz, 1H), 6.26 (d, *J* = 7.8 Hz, 1H), 5.97 (s, 1H), 4.88–4.78 (m, 1H), 4.64 (d, *J* = 16.9 Hz, 1H), 3.36–3.26 (m, 2H), 3.20–3.10 (m, 2H), 2.39–2.29 (m, 6H), 2.19–2.10 (m, 1H). ^13^C NMR (100 MHz, Acetone-*d*_6_) δ 164.61, 150.71, 149.51, 143.58, 137.20, 137.01, 135.99, 135.34, 131.44, 130.38, 130.02, 129.46, 128.56, 127.07, 126.68, 126.36, 126.19, 125.75, 125.63, 123.43, 121.80, 120.85, 117.37, 105.77, 82.80, 57.42, 47.59, 47.08, 39.73, 37.62, 19.36, 18.49. MS (ESI(+)) calcd for C_32_H_30_ClN_3_O^+^ [M + H]^+^: 508.2; found: 508.2.

(3a,8-bis(2-methylbenzyl)-3,3a,8,8a-tetrahydropyrrolo[2,3-b]indol-1(2H)-yl)(2-chloropyridin-4-yl) methanone(**a19**): Yield: 73%. White solid. Melting point: 147–149 °C. R_f_ = 0.26 (PE/EA 6:1). ^1^H NMR (400 MHz, DMSO-*d*_6_) δ 8.43 (d, *J* = 5.0 Hz, 1H), 7.41 (s, 1H), 7.36 (d, *J* = 4.8 Hz, 1H), 7.14–6.92 (m, 10H), 6.72 (d, *J* = 7.2 Hz, 1H), 6.56 (t, *J* = 7.4 Hz, 1H), 6.19 (d, *J* = 7.8 Hz, 1H), 5.81 (s, 1H), 4.57 (d, *J* = 4.9 Hz, 2H), 3.25 (dd, *J* = 11.0, 5.8 Hz, 1H), 3.14 (d, *J* = 13.6 Hz, 1H), 3.03 (d, *J* = 13.7 Hz, 1H), 2.29–2.17 (m, 5H), 1.94 (s, 3H). ^13^C NMR (100 MHz, DMSO-*d*_6_) δ 166.46, 151.03, 150.90, 150.55, 147.25, 137.42, 137.21, 136.30, 135.56, 131.56, 130.73, 130.68, 130.46, 128.95, 127.11, 126.77, 126.27, 126.15, 126.05, 123.85, 122.46, 121.34, 117.53, 106.14, 83.22, 57.13, 49.33, 47.17, 37.57, 20.09, 19.39. MS (ESI(+)) calcd for C_32_H_30_ClN_3_O^+^ [M + H]^+^: 508.2; found: 508.2.

(3a,8-bis(2-methylbenzyl)-1,2,3,3a,8,8a-hexahydropyrrolo[2,3-b]indole-1-carbonyl) benzaldehyde(**a20**): Yield: 87%. White solid. Melting point: 158–160 °C. R_f_ = 0.24 (PE/EA 5:1). ^1^ H NMR (400 MHz, Acetone-*d*_6_) δ 7.72–7.65 (m, 3H), 7.59–7.54 (m, 1H), 7.28–7.20 (m, 4H), 7.10 (td, *J* = 7.3, 1.3 Hz, 2H), 7.02–6.93 (m, 3H), 6.45 (dd, *J* = 16.3, 7.7 Hz, 2H), 6.11 (d, *J* = 7.1 Hz, 1H), 6.06 (s, 1H), 5.19 (s, 1H), 4.52 (d, *J* = 9.7 Hz, 1H), 3.01 (s, 1H), 2.87 (d, *J* = 13.3 Hz, 1H), 2.39 (s, 3H), 2.09 (s, 5H), 1.51 (s, 3H). ^13^C NMR (100 MHz, Acetone-*d*_6_) δ 169.23, 150.83, 137.32, 136.16, 135.38, 135.25, 133.46, 131.92, 130.47, 130.05, 129.34, 128.97, 128.47, 128.31, 126.89, 126.68, 126.37, 126.27, 126.24, 125.77, 125.65, 125.11, 124.79, 123.85, 123.37, 117.19, 105.67, 57.29, 48.22, 39.76, 37.45, 19.39, 18.51. MS(ESI(+)) calcd for C_33_H_32_N_3_O_2_^+^ [M + H]^+^: 502.2; found: 502.2.

(3a,8-bis(3-fluorobenzyl)-3,3a,8,8a-tetrahydropyrrolo[2,3-b]indol-1(2H)-yl)(2-fluoropyridin-3-yl) methanone(**b1**): White solid. Yield: 89%. Melting point: 92–94 °C. R_f_ = 0.24 (PE/EA 4:1). ^1^H NMR (400 MHz, Acetone-*d*_6_) δ 8.31 (ddd, *J* = 4.8, 1.8, 1.1 Hz, 1H), 7.93 (ddd, *J* = 9.3, 7.4, 2.0 Hz, 1H), 7.41 (ddd, *J* = 7.2, 4.9, 2.1 Hz, 1H), 7.28–7.16 (m, 3H), 7.05–6.92 (m, 3H), 6.88–6.80 (m, 3H), 6.76–6.66 (m, 2H), 6.14 (d, *J* = 7.9 Hz, 1H), 6.00 (s, 1H), 4.55 (s, 2H), 3.48 (ddd, *J* = 5.7, 5.2, 4.1 Hz, 1H), 3.35–3.26 (m, 2H), 3.10 (d, *J* = 13.3 Hz, 1H), 2.36–2.28 (m, 2H). ^13^C NMR (100 MHz, Acetone-*d*_6_) δ 163.97 (d, *J* = 4.6 Hz), 162.98 (d, *J* = 243.5 Hz), 162.38 (d, *J* = 243.1 Hz), 158.85 (d, *J* = 236.8 Hz), 150.74, 149.13 (d, *J* = 15.1 Hz), 142.53 (d, *J* = 6.6 Hz), 140.52 (d, *J* = 10.4 Hz), 131.26, 130.06 (d, *J* = 8.1 Hz), 129.58 (d, *J* = 8.1 Hz), 128.70, 125.94, 123.54, 122.46, 122.09 (d, *J* = 4.1 Hz), 119.52 (d, *J* = 33.4 Hz), 117.85, 116.55 (d, *J* = 21.2 Hz), 113.37 (d, *J* = 21.3 Hz), 113.25 (d, *J* = 21.2 Hz), 106.20, 82.56, 57.08, 49.29, 47.82, 44.21 (d, *J* = 12.2 Hz), 38.02. MS (ESI(+)) calcd for C_30_H_24_F_3_N_3_O^+^ [M + H]^+^: 500.2; found: 500.2.

(3a,8-bis(3-fluorobenzyl)-3,3a,8,8a-tetrahydropyrrolo[2,3-b]indol-1(2H)-yl)(6-chloropyridin-2-yl) methanone(**b2**): Yield: 83%. Yellow oily liquid. R_f_ = 0.23 (PE/EA 1:1). ^1^H NMR (400 MHz, Acetone-*d*_6_) δ 7.99–7.91 (m, 1H), 7.81–7.67 (m, 1H), 7.61–7.50 (m, 1H), 7.24–7.15 (m, 3H), 6.98 (ddd, *J* = 23.4, 14.7, 8.8 Hz, 3H), 6.87–6.80 (m, 3H), 6.71 (dd, *J* = 9.8, 6.8 Hz, 2H), 6.13 (dd, *J* = 7.4, 3.0 Hz, 1H), 6.06–5.98 (m, 1H), 4.63–4.46 (m, 2H), 3.88 (t, *J* = 7.9 Hz, 1H), 3.47–3.40 (m, 1H), 3.26 (dd, *J* = 13.2, 3.4 Hz, 1H), 3.12–3.07 (m, 1H), 2.37–2.26 (m, 2H). ^13^C NMR (100 MHz, Acetone-*d*_6_) δ 165.85, 163.59, 161.17, 154.41, 150.60, 149.33, 142.60, 140.39, 131.53, 130.09, 129.61, 128.62, 126.01, 125.82, 123.48, 122.84, 122.55, 117.78, 116.71, 116.50, 113.47, 113.33, 113.11, 106.31, 82.99, 56.44, 49.29, 48.82, 44.05, 38.02. MS (ESI(+)) calcd for C_30_H_24_ClF_2_N_3_O^+^ [M + H]^+^: 516.2; found: 516.2.

(3a,8-bis(3-fluorobenzyl)-3,3a,8,8a-tetrahydropyrrolo[2,3-b]indol-1(2H)-yl)(5-methylpyridin-3-yl) methanone(**b3**): Yield: 89%. White solid. Melting point: 163–165 °C. R_f_ = 0.23 (PE/EA 4:1). ^1^H NMR (400 MHz, DMSO-*d*_6_) δ 8.51 (d, *J* = 1.3 Hz, 1H), 8.43 (d, *J* = 1.5 Hz, 1H), 7.67 (s, 1H), 7.27–6.67 (m, 12H), 6.12 (d, *J* = 7.8 Hz, 1H), 5.98 (s, 1H), 4.48 (s, 2H), 3.48 (d, *J* = 6.1 Hz, 1H), 3.24 (d, *J* = 13.1 Hz, 1H), 3.06 (d, *J* = 13.2 Hz, 1H), 2.33 (s, 3H), 2.26–2.19 (m, 2H). ^13^C NMR (100 MHz, DMSO-*d*_6_) δ 167.99, 162.72 (d, *J* = 243.1 Hz), 162.17 (d, *J* = 242.5 Hz), 151.83, 150.69, 145.72, 142.72 (d, *J* = 6.6 Hz), 140.92 (d, *J* = 7.4 Hz), 135.70, 133.24, 131.63 (d, *J* = 5.8 Hz), 130.63 (d, *J* = 8.2 Hz), 130.05 (d, *J* = 8.3 Hz), 129.03, 126.35, 124.00, 122.72, 117.89, 116.88, 113.81 (d, *J* = 21.7 Hz), 113.79 (d, *J* = 19.1 Hz), 113.56, 113.35, 106.15, 82.34, 56.76, 49.38, 48.91, 44.01, 38.63, 18.18. MS (ESI(+)) calcd for C_31_H_27_F_2_N_3_O^+^ [M + H]^+^: 496.2; found: 496.2.

3a,8-bis(3-fluorobenzyl)-3,3a,8,8a-tetrahydropyrrolo[2,3-b]indol-1(2H)-yl)(5-chloropyrazin-2-yl) methanone(**b4**): Yield: 84%. Yellow solid. Melting point: 168–170 °C. R_f_ = 0.23 (PE/EA 8:1). ^1^H NMR (400 MHz, Acetone-*d*_6_) δ 8.73–8.71 (m, 1H), 8.69–8.67 (m, 1H), 7.22 (ddd, *J* = 8.1, 5.0, 2.1 Hz, 2H), 7.17 (dd, *J* = 7.3, 0.8 Hz, 1H), 7.02–6.97 (m, 2H), 6.90–6.78 (m, 4H), 6.75–6.67 (m, 2H), 6.34–6.24 (m, 1H), 6.16 (d, *J* = 7.8 Hz, 1H), 6.04 (s, 1H), 4.58 (d, *J* = 17.2 Hz, 2H), 3.48–3.41 (m, 1H), 3.26 (d, *J* = 13.3 Hz, 1H), 3.09 (d, *J* = 13.3 Hz, 1H), 2.35–2.27 (m, 2H). ^13^C NMR (100 MHz, Acetone-*d*_6_) δ 164.70, 162.94 (d, *J* = 243.7 Hz), 150.54, 149.83, 147.64, 146.04, 142.47, 141.34, 140.52 (d, *J* = 7.5 Hz), 131.49, 130.06 (d, *J* = 8.1 Hz), 129.59 (d, *J* = 8.2 Hz), 128.65, 126.02, 123.49, 122.56, 118.04 (d, *J* = 34.2 Hz), 116.60 (d, *J* = 21.3 Hz), 113.41 (d, *J* = 27.9 Hz), 113.31 (d, *J* = 33.7 Hz), 113.14, 106.34, 83.08, 56.46, 49.36, 48.66, 43.99, 38.67, 37.96. MS (ESI(+)) calcd for C_29_H_23_ClF_2_N_4_O^+^ [M + H]^+^: 518.2; found: 518.2.

(3a,8-bis(3-fluorobenzyl)-3,3a,8,8a-tetrahydropyrrolo[2,3-b]indol-1(2H)-yl)(6-methylpyridin-3-yl) methanone(**b5**): Yield: 86%. White solid. Melting point: 180–182 °C. R_f_ = 0.32 (PE/EA 8:1). ^1^H NMR (400 MHz, Acetone-*d*_6_) δ 8.54 (s, 1H), 7.73 (d, *J* = 6.3 Hz, 1H), 7.31–7.12 (m, 4H), 7.11–6.77 (m, 6H), 6.77–6.64 (m, 2H), 6.21–6.03 (m, 2H), 4.63–4.40 (m, 2H), 3.70–3.51 (m, 1H), 3.50–3.36 (m, 1H), 3.28 (dd, *J* = 13.2, 3.3 Hz, 1H), 3.09 (d, *J* = 11.3 Hz, 1H), 2.55–2.43 (m, 3H), 2.37–2.24 (m, 2H). ^13^C NMR (100 MHz, Acetone-*d*_6_) δ 168.01, 163.58, 161.16, 160.31, 150.78, 148.06, 142.72, 140.69, 135.40, 131.36, 130.02, 129.59, 129.26, 128.66, 126.00, 123.55, 122.31, 117.65, 116.67, 116.46, 113.45, 113.24, 113.09, 106.10, 82.50, 56.67, 49.22, 48.96, 44.18, 38.25, 23.67. MS (ESI(+)) calcd for C_31_H_27_F_2_N_3_O^+^ [M + H]^+^: 496.2; found: 496.2.

(3a,8-bis(3-fluorobenzyl)-3,3a,8,8a-tetrahydropyrrolo[2,3-b]indol-1(2H)-yl)(2-hydroxypyridin-3-yl) methanone(**b6**): Yield: 84%. Yellow oily liquid. R_f_ = 0.25 (PE/EA 4:1). ^1^H NMR (400 MHz, Acetone-d_6_) δ 7.61–7.52 (m, 2H), 7.26–7.18 (m, 2H), 7.15 (d, J = 7.3 Hz, 1H), 7.00–6.78 (m, 7H), 6.71–6.64 (m, 2H), 6.31 (t, J = 6.6 Hz, 1H), 6.06 (d, J = 7.9 Hz, 1H), 5.94 (s, 1H), 4.53 (q, J = 16.7 Hz, 2H), 3.58–3.51 (m, 2H), 3.31–3.26 (m, 1H), 3.06 (d, J = 13.2 Hz, 1H), 2.31–2.20 (m, 2H). ^13^C NMR (100 MHz, Acetone-d_6_) δ 171.51, 166.53, 163.55, 161.13, 159.50, 150.88, 142.74, 141.09, 140.73, 137.05, 131.45, 130.02, 129.54, 128.48, 125.98, 123.41, 122.61, 117.46, 116.64, 113.56, 113.34, 112.98, 105.98, 105.09, 82.33, 56.97, 49.24, 47.31, 44.21, 38.49. MS (ESI (+)) calcd for C_30_H_25_F_2_N_3_O_2_^+^ [M + H]^+^: 498.2; found: 498.

(3a,8-bis(3-fluorobenzyl)-3,3a,8,8a-tetrahydropyrrolo[2,3-b]indol-1(2H)-yl)(5,6-dichloropyridin-3-yl) methanone(**b7**): Yield: 89%. White solid. Melting point: 126–128 °C. R_f_ = 0.34 (PE/EA 8:1). ^1^H NMR (400 MHz, DMSO-*d*_6_) δ 8.40 (d, *J* = 2.1 Hz, 1H), 8.15 (d, *J* = 1.9 Hz, 1H), 7.22–6.88 (m, 7H), 6.75–6.59 (m, 5H), 6.07 (d, *J* = 7.8 Hz, 1H), 5.85 (s, 1H), 4.40 (s, 2H), 3.49 (dt, *J* = 15.1, 7.5 Hz, 1H), 3.17 (d, *J* = 13.2 Hz, 1H), 2.97 (d, *J* = 13.2 Hz, 1H), 2.19 (d, *J* = 4.6 Hz, 2H). ^13^C NMR (100 MHz, DMSO-*d*_6_) δ 165.45, 163.93, 161.50, 150.71, 149.36, 146.88, 142.61, 140.80, 138.75, 132.92, 131.49, 130.68, 130.12, 129.68, 129.05, 126.34, 124.01, 122.74, 117.96, 116.85, 116.64, 113.90, 113.69, 113.39, 106.24, 82.39, 56.87, 49.15, 48.86, 44.01. MS (ESI(+)) calcd for C_30_H_23_Cl_2_F_2_N_3_O^+^ [M + H]^+^: 550.1; found: 550.1.

(2-aminopyridin-3-yl)(3a,8-bis(3-fluorobenzyl)-3,3a,8,8a-tetrahydropyrrolo[2,3-b]indol-1(2H)-yl) methanone(**b8**): Yield: 85%. White solid. Melting point: 181–183 °C. R_f_ = 0.24 (PE/EA 3:1). ^1^H NMR (400 MHz, DMSO-*d*_6_) δ 7.95 (d, *J* = 3.5 Hz, 1H), 7.37–6.83 (m, 8H), 6.79–6.42 (m, 6H), 6.22–5.82 (m, 4H), 4.37 (s, 2H), 3.45 (s, 1H), 3.18 (d, *J* = 13.0 Hz, 1H), 3.00 (d, *J* = 13.1 Hz, 1H), 2.16 (s, 2H). ^13^C NMR (100 MHz, DMSO-*d*_6_) δ 162.15 (d, *J* = 242.7 Hz), 161.48, 157.30, 150.83, 150.30, 141.04 (d, *J* = 6.9 Hz), 137.04, 131.51, 130.59 (d, *J* = 8.4 Hz), 130.02 (d, *J* = 8.3 Hz), 128.99, 126.30, 124.07, 122.60, 117.84, 116.75 (d, *J* = 20.8 Hz), 113.88, 113.67, 113.40 (d, *J* = 21.4 Hz), 111.95, 105.98, 82.04, 56.89, 48.78, 44.09, 38.99. MS (ESI(+)) calcd for C_30_H_26_F_2_N_4_O^+^ [M + H]^+^: 497.2; found: 497.2.

(3a,8-bis(3-fluorobenzyl)-3,3a,8,8a-tetrahydropyrrolo[2,3-b]indol-1(2H)-yl)(naphthalen-2-yl) methanone(**b9**): Yield: 87%. White solid. Melting point: 96–98 °C. R_f_ = 0.21 (PE/EA 10:1). ^1^H NMR (400 MHz, Acetone-*d*_6_) δ 7.99–7.88 (m, 2H), 7.69 (d, *J* = 8.2 Hz, 1H), 7.49 (tt, *J* = 11.0, 7.1 Hz, 3H), 7.36–7.21 (m, 3H), 7.15 (d, *J* = 7.3 Hz, 1H), 7.09–6.89 (m, 6H), 6.80 (d, *J* = 10.3 Hz, 1H), 6.74–6.67 (m, 1H), 6.28–6.18 (m, 2H), 4.81–4.66 (m, 2H), 3.28 (t, *J* = 11.2 Hz, 1H), 3.23–3.12 (m, 2H), 3.12–3.00 (m, 1H), 2.43–2.14 (m, 2H). ^13^C NMR (100 MHz, DMSO-*d*_6_),) δ 169.20, 163.03 (d, *J* = 243.7 Hz), 162.47 (d, *J* = 243.5 Hz), 150.83, 142.88 (d, *J* = 6.8 Hz), 140.64 (d, *J* = 7.0 Hz), 135.19, 133.57, 131.69, 130.16 (d, *J* = 8.2 Hz), 129.68 (d, *J* = 8.2 Hz), 129.43, 129.16, 128.66, 128.44, 126.95, 126.40, 126.17, 125.19, 124.86, 123.98, 123.53, 122.65, 117.73, 116.87, 113.46 (d, *J* = 31.3 Hz), 113.40, 113.36 (d, *J* = 29.8 Hz), 106.01, 82.30, 57.12, 49.41, 48.05, 44.07, 38.19. MS (ESI(+)) calcd for C_35_H_28_F_2_N_2_O^+^ [M + H]^+^: 531.3; found: 531.3.

(3a,8-bis(3-fluorobenzyl)-3,3a,8,8a-tetrahydropyrrolo[2,3-b]indol-1(2H)-yl)(2-chloropyridin-3-yl) methanone(**b10**): Yield: 91%. White solid. Melting point: 180–182 °C. R_f_ = 0.24 (PE/EA 2:1). ^1^H NMR (400 MHz, Acetone-*d*_6_) δ 8.45 (dd, *J* = 4.1, 1.9 Hz, 1H), 7.70 (s, 1H), 7.52–7.42 (m, 1H), 7.30–7.17 (m, 3H), 7.02–6.85 (m, 5H), 6.72 (ddd, *J* = 7.3, 3.7, 0.8 Hz, 2H), 6.17 (d, *J* = 7.5 Hz, 1H), 5.99 (s, 1H), 4.61 (dd, *J* = 38.0, 16.7 Hz, 2H), 3.38–3.28 (m, 2H), 3.15 (t, *J* = 21.4 Hz, 2H), 2.37–2.26 (m, 2H), 2.10–2.08 (m, 1H). ^13^C NMR (100 MHz, Acetone-*d*_6_) δ 165.42, 164.18, 161.76, 150.76, 150.24, 146.36, 142.56, 140.53, 137.14, 133.03, 131.36, 130.06, 129.61, 128.69, 126.04, 123.52, 123.20, 122.57, 117.85, 116.53, 113.59, 113.40, 113.19, 106.12, 82.29, 57.28, 49.21, 47.45, 44.00, 38.22. MS (ESI(+)) calcd for C_30_H_24_ClF_2_N_3_O^+^ [M + H]^+^: 516.2; found: 516.2.

(3a,8-bis(3-fluorobenzyl)-3,3a,8,8a-tetrahydropyrrolo[2,3-b]indol-1(2H)-yl)(pyrazin-2-yl) methanone(**b11**): Yield: 82%. Light yellow solid. Melting point: 142–144 °C. R_f_ = 0.23 (PE/EA 4:1). ^1^H NMR (400 MHz, DMSO-*d*_6_) δ 8.87 (d, *J* = 1.5 Hz, 1H), 8.75 (d, *J* = 2.6 Hz, 1H), 8.64 (dd, *J* = 2.5, 1.5 Hz, 1H), 7.22–7.17 (m, 3H), 7.02–6.96 (m, 2H), 6.81–6.65 (m, 6H), 6.08 (d, *J* = 7.8 Hz, 1H), 5.94 (s, 1H), 4.46 (s, 2H), 3.76–3.67 (m, 1H), 3.33–3.25 (m, 1H), 3.18 (s, 1H), 3.04 (s, 1H), 2.23–2.14 (m, 2H). ^13^C NMR (100 MHz, DMSO-*d*_6_) δ 166.00, 163.94, 160.98, 150.47, 149.25, 146.56, 145.29, 143.54, 142.47, 140.87, 131.71, 130.59, 130.14, 129.02, 126.40, 123.97, 122.84, 118.08, 116.92, 116.71, 113.95, 113.75, 113.49, 106.34, 82.68, 56.55, 49.14, 48.96, 43.82. MS (ESI(+)) calcd for C_29_H_24_F_2_N_4_O ^+^ [M + H]^+^: 482.2; found: 482.2.

(3a,8-bis(3-fluorobenzyl)-3,3a,8,8a-tetrahydropyrrolo[2,3-b]indol-1(2H)-yl)(pyridin-2-yl) methanone(**b12**): Yield: 84%. White solid. Melting point: 137–139 °C. R_f_ = 0.23 (PE/EA 6:1). ^1^H NMR (400 MHz, DMSO-*d*_6_) δ 8.53 (d, *J* = 4.7 Hz, 1H), 7.88 (td, *J* = 7.8, 1.3 Hz, 1H), 7.62 (d, *J* = 7.8 Hz, 1H), 7.49–7.42 (m, 1H), 7.17 (dd, *J* = 14.8, 7.9 Hz, 3H), 7.01–6.88 (m, 4H), 6.76–6.63 (m, 5H), 6.02 (d, *J* = 7.9 Hz, 1H), 5.91 (s, 1H), 4.43 (s, 2H), 3.65 (dd, *J* = 11.4, 6.9 Hz, 1H), 3.16 (d, *J* = 13.2 Hz, 1H), 3.02–2.96 (m, 1H), 2.14 (ddd, *J* = 20.1, 10.6, 5.2 Hz, 2H). ^13^C NMR (100 MHz, DMSO-*d*_6_) δ 167.79, 162.75 (d, *J* = 243.4 Hz), 162.19 (d, *J* = 242.7 Hz), 154.05, 150.58, 148.70, 142.60 (d, *J* = 6.7 Hz), 140.91 (d, *J* = 7.3 Hz), 137.79, 131.79, 130.64, 130.60 (d, *J* = 8.0 Hz), 130.06 (d, *J* = 8.8 Hz), 128.96, 126.38, 125.80, 123.99 (d, *J* = 11.0 Hz), 122.84, 121.98, 117.97, 116.81 (d, *J* = 20.9 Hz), 113.82 (d, *J* = 19.1 Hz), 113.62 (d, *J* = 21.0 Hz), 112.99 (d, *J* = 21.4 Hz), 106.27, 82.60, 56.52, 49.09 (d, J = 8.6 Hz), 43.93, 38.50. MS (ESI(+)) calcd for C_30_H_25_F_2_N_3_O^+^ [M + H]^+^: 482.2; found: 482.2.

(3a,8-bis(3-fluorobenzyl)-3,3a,8,8a-tetrahydropyrrolo[2,3-b]indol-1(2H)-yl)(6-chloropyridin-3-yl) methanone(**b13**): Yield: 87%. White solid. Melting point: 119–121 °C. R_f_ = 0.31 (PE/EA 6:1). ^1^H NMR (400 MHz, DMSO-*d*_6_) δ 8.46 (d, *J* = 2.2 Hz, 1H), 7.92 (dd, *J* = 8.3, 2.4 Hz, 1H), 7.56 (d, *J* = 8.3 Hz, 1H), 7.25–6.61 (m, 12H), 6.09 (d, *J* = 7.8 Hz, 1H), 5.92 (s, 1H), 4.43 (s, 2H), 3.53–3.45 (m, 1H), 3.20 (d, *J* = 13.1 Hz, 1H), 3.01 (d, *J* = 13.2 Hz, 1H), 2.21 (dt, *J* = 13.5, 6.8 Hz, 2H). ^13^C NMR (100 MHz, DMSO-*d*_6_) δ 166.79, 162.71 (d, *J* = 243.5 Hz), 162.16 (d, J = 242.7 Hz), 152.11, 150.68, 149.13, 142.65 (d, *J* = 6.6 Hz), 140.87 (d, *J* = 7.6 Hz), 139.34, 131.53, 130.63 (d, *J* = 8.2 Hz), 130.06 (d, *J* = 8.5 Hz), 129.05, 126.33, 124.55, 124.01, 122.69, 117.95, 116.75 (d, *J* = 20.7 Hz), 113.74 (d, *J* = 31.5 Hz), 113.54 (d, *J* = 33.6 Hz), 106.22, 82.43, 56.80, 49.30, 48.92, 44.02, 38.60. MS (ESI(+)) calcd for C_30_H_24_ClF_2_N_3_O^+^ [M + H]^+^: 516.2; found: 516.2.

(3a,8-bis(3-fluorobenzyl)-3,3a,8,8a-tetrahydropyrrolo[2,3-b]indol-1(2H)-yl)(4-methoxyphenyl) methanone(**b14**): Yield: 89%. White solid. Melting point: 204–206 °C. R_f_ = 0.26 (PE/EA 6:1). ^1^H NMR (400 MHz, Acetone-*d*_6_) δ 7.44 (d, *J* = 7.4 Hz, 2H), 7.18 (ddd, *J* = 18.5, 10.8, 9.3 Hz, 3H), 7.01–6.79 (m, 8H), 6.71–6.64 (m, 2H), 6.12–6.02 (m, 2H), 4.54 (d, *J* = 16.2 Hz, 1H), 4.41 (d, *J* = 16.2 Hz, 1H), 3.80 (s, 3H), 3.72–3.56 (m, 1H), 3.36 (s, 1H), 3.23 (d, *J* = 13.2 Hz, 1H), 3.05 (d, *J* = 13.3 Hz, 1H), 2.30–2.17 (m, 2H). ^13^C NMR (100 MHz, Acetone-*d*_6_) δ 169.59, 162.92 (d, *J* = 244.0 Hz), 162.38 (d, *J* = 243.2 Hz), 161.38, 150.86, 140.76, 140.69, 130.01 (d, *J* = 8.3 Hz), 129.68, 129.52 (d, *J* = 8.3 Hz), 128.58, 128.45, 126.01, 123.52, 122.42, 117.56, 116.58 (d, *J* = 21.2 Hz), 113.32 (d, *J* = 21.6 Hz), 113.31, 113.05, 106.06, 82.42, 54.91, 48.83, 44.24, 38.29. MS (ESI(+)) calcd for C_32_H_28_F_2_N_2_O_2_^+^ [M + H]^+^: 511.2; found: 511.2.

(3a,8-bis(3-fluorobenzyl)-3,3a,8,8a-tetrahydropyrrolo[2,3-b]indol-1(2H)-yl)(5-methylpyrazin-2-yl) methanone(**b15**): Yield: 82%. Light yellow solid. Melting point: 147–149 °C. R_f_ = 0.21 (PE/EA 5:1). ^1^H NMR (400 MHz, DMSO-*d*_6_) δ 8.74 (d, *J* = 1.2 Hz, 1H), 8.54–8.50 (m, 1H), 7.19 (dd, *J* = 14.6, 7.0 Hz, 3H), 7.04–6.93 (m, 4H), 6.79 (d, *J* = 7.7 Hz, 1H), 6.75–6.65 (m, 4H), 6.07 (t, *J* = 6.1 Hz, 1H), 5.95 (s, 1H), 4.46 (s, 2H), 3.78–3.70 (m, 1H), 3.18 (d, *J* = 13.2 Hz, 1H), 3.02 (d, *J* = 13.1 Hz, 1H), 2.54 (s, 3H), 2.26–2.16 (m, 2H). ^13^C NMR (100 MHz, DMSO-*d*_6_) δ 166.18, 163.39, 160.98, 155.80, 150.48, 146.34, 145.17, 144.28, 142.92, 142.57, 141.60, 140.88, 131.74, 130.57, 130.12, 129.00, 126.39, 123.95, 122.84, 118.04, 116.92, 113.93, 113.71, 113.49, 106.33, 82.69, 56.49, 48.99, 43.85, 21.80. MS (ESI(+)) calcd for C_30_H_26_F_2_N_4_O^+^ [M + H]^+^: 497.2; found: 497.2.

(3a,8-bis(3-fluorobenzyl)-3,3a,8,8a-tetrahydropyrrolo[2,3-b]indol-1(2H)-yl)(pyridin-3-yl) methanone(**b16**): Yield: 90%. White solid. Melting point: 146–148 °C. R_f_ = 0.23 (PE/EA 3:1). ^1^H NMR (400 MHz, Acetone-*d*_6_) δ 8.48 (d, *J* = 2.0 Hz, 1H), 7.91 (dd, *J* = 8.2, 2.3 Hz, 1H), 7.50 (d, *J* = 8.2 Hz, 1H), 7.27–7.15 (m, 3H), 7.07–6.65 (m, 9H), 6.16 (d, *J* = 7.8 Hz, 1H), 6.06 (s, 1H), 4.57 (d, *J* = 16.8 Hz, 1H), 4.49 (d, *J* = 16.8 Hz, 1H), 3.72–3.61 (m, 1H), 3.46 (td, *J* = 10.9, 6.4 Hz, 1H), 3.32–3.23 (m, 1H), 3.12–3.05 (m, 1H), 2.38–2.27 (m, 2H). ^13^C NMR (100 MHz, Acetone-*d*_6_) δ 166.68, 164.12, 161.16, 152.28, 150.73, 148.82, 142.60, 140.61, 138.51, 131.44, 131.28, 130.12, 129.53, 128.69, 125.98, 123.91, 123.55, 122.42, 117.74, 116.66, 116.45, 113.47, 113.26, 113.12, 106.15, 82.62, 56.74, 49.13, 44.13, 38.19. MS (ESI(+)) calcd for C_30_H_25_F_2_N_3_O^+^ [M + H]^+^: 482.2; found: 482.2.

(3a,8-bis(3-fluorobenzyl)-3,3a,8,8a-tetrahydropyrrolo[2,3-b]indol-1(2H)-yl)(pyridin-4-yl) methanone(**b17**): Yield: 87%. White solid. Melting point: 176–178 °C. R_f_ = 0.24 (PE/EA 3:1). ^1^H NMR (400 MHz, DMSO-*d*_6_) δ 8.64 (dd, *J* = 4.4, 1.5 Hz, 2H), 7.37 (dd, *J* = 4.4, 1.6 Hz, 2H), 7.28–6.91 (m, 7H), 6.72 (ddd, *J* = 26.3, 18.9, 9.7 Hz, 5H), 6.09 (d, *J* = 7.8 Hz, 1H), 5.91 (s, 1H), 4.45 (s, 2H), 3.45–3.37 (m, 1H), 3.20 (d, *J* = 13.1 Hz, 1H), 3.03 (d, *J* = 13.2 Hz, 1H), 2.20 (dd, *J* = 10.5, 6.2 Hz, 2H). ^13^C NMR (100 MHz, DMSO-*d*_6_) δ 167.93, 162.75 (d, *J* = 243.4 Hz, 15C), 150.68, 150.51, 143.66, 142.71, 142.65, 140.86, 131.56, 130.70, 130.62, 130.11, 130.03, 129.07, 126.36, 124.02, 122.73, 121.89, 118.00, 116.81 (d, *J* = 20.9 Hz, 14C), 113.94, 113.70, 113.60, 113.38, 106.22, 82.47, 56.84, 49.07, 43.95. MS (ESI (+)) calcd for C_30_H_25_F_2_N_3_O^+^ [M + H]^+^: 482.2; found: 482.2.

(3a,8-bis(3-fluorobenzyl)-3,3a,8,8a-tetrahydropyrrolo[2,3-b]indol-1(2H)-yl)(3-chloropyridin-4-yl) methanone(**b18**): Yield: 91%. White solid. Melting point: 154–156 °C. R_f_ = 0.24 (PE/EA 3:1). ^1^H NMR (400 MHz, Acetone-*d*_6_) δ 8.65 (s, 1H), 8.57 (d, *J* = 4.8 Hz, 1H), 7.29–7.18 (m, 4H), 7.04–6.93 (m, 3H), 6.86 (dd, *J* = 15.6, 9.7 Hz, 3H), 6.75–6.70 (m, 2H), 6.18 (d, *J* = 7.9 Hz, 1H), 5.99 (s, 1H), 4.60 (q, *J* = 16.7 Hz, 2H), 3.34–3.28 (m, 2H), 3.21–3.09 (m, 2H), 2.38–2.28 (m, 2H). ^13^C NMR (100 MHz, Acetone-*d*_6_) δ 164.64, 162.98 (d, *J* = 244.3 Hz), 162.43 (d, *J* = 243.6 Hz), 150.69, 149.62, 148.58, 143.54, 142.51 (d, *J* = 6.8 Hz), 140.45 (d, *J* = 7.6 Hz), 131.32, 130.11 (d, *J* = 8.4 Hz), 129.66 (d, *J* = 8.5 Hz), 128.71, 127.16, 126.05, 123.52, 122.57, 121.83, 117.92, 116.64 (d, *J* = 21.3 Hz), 113.48 (d, *J* = 21.9 Hz), 113.42 (d, *J* = 21.2 Hz), 113.32 (d, *J* = 21.3 Hz), 106.18, 82.35, 57.26, 49.28, 47.31, 43.94, 38.20. MS (ESI(+)) calcd for C_30_H_24_ClF_2_N_3_O^+^ [M + H]^+^: 516.2; found: 516.2.

(3a,8-bis(3-fluorobenzyl)-3,3a,8,8a-tetrahydropyrrolo[2,3-b]indol-1(2H)-yl)(2-chloropyridin-4-yl) methanone(**b19**): Yield: 79%. White solid. Melting point: 134–136 °C. R_f_ = 0.26 (PE/EA 6:1). ^1^H NMR (400 MHz, Acetone-*d*_6_) δ 8.69–8.60 (m, 2H), 7.84 (d, *J* = 7.9 Hz, 1H), 7.41 (dd, *J* = 7.7, 4.9 Hz, 1H), 7.21 (ddd, *J* = 21.1, 11.4, 4.7 Hz, 3H), 7.05–6.97 (m, 2H), 6.86 (dd, *J* = 12.1, 4.9 Hz, 2H), 6.80 (d, *J* = 10.1 Hz, 1H), 6.75–6.67 (m, 2H), 6.15 (d, *J* = 7.9 Hz, 1H), 6.08 (s, 1H), 4.59 (d, *J* = 16.8 Hz, 1H), 4.50 (d, *J* = 16.8 Hz, 1H), 3.66–3.57 (m, 1H), 3.44 (td, *J* = 10.8, 6.5 Hz, 1H), 3.28 (d, *J* = 13.2 Hz, 1H), 3.14–3.04 (m, 1H), 2.35–2.27 (m, 2H). ^13^C NMR (100 MHz, Acetone-*d*_6_) δ 166.33, 163.58, 161.16, 151.31, 150.69, 150.30, 147.24, 142.60, 140.56, 131.23, 130.13, 129.55, 128.70, 125.95, 123.54, 122.44, 122.09, 120.67, 117.79, 116.65, 116.44, 113.41, 113.28, 113.15, 106.16, 82.69, 56.85, 48.77, 44.07, 38.16. MS (ESI (+)) calcd for C_30_H_24_ClF_2_N_3_O^+^ [M + H]^+^: 516.2; found: 516.2.

### 3.3. Biological Activity

The antifungal activity of calycanthaceous alkaloid analogues was determined by the method previously reported [20]. Six kinds of plant phytopathogenic fungi (*Verticillium dahliae-ACCC 36211; Colletotrichum orbiculare-SAUM 0321; Cytospora juglandis-ACCC36357; Curvularia lunata-SAUM 1373I; Sclerotinia sclerotiorum-ACCC34236; Altenaria solani-SAUM 1275)* were provided by the School of Environmental and Chemical Engineering, Jiangsu University of Science and Technology.

The mics of 96-well plate compounds were used for testing. The antibacterial compounds were dissolved in 5% dimethyl sulfoxide (DMSO) at a concentration of 1.02 mg/mL. We added 100 μL of the solution to the first well, absorbed 100 μL from the first well and added it to the second well, and then absorbed 100 μL and added it to the third well and so on until the 10th column. We added 100 µL of prepared bacterial solution to each well. The 11th column of the plate was reserved for the negative control well (without inocula) and the 12th column for the positive control well (without antibacterial agents). Solutions of 500, 250, 125, 62.5, 31.3, 15.6, 7.8, 3.9 and 1.9 μg/mL were obtained by the microdilution method. The antibacterial test plates were incubated aerobically at 37 °C for 24 h, and the minimum sterilized concentration was recorded. The reference strains used were *polygonum* and *amphotericin*. Detection of MICs, MBC and MFC was determined by 10 μL plating on LB Agar and Sabouraud Dextrose Agar with negative and positive control wells, respectively. The above tests were repeated three times and were further repeated if the results were different.

## 4. Conclusions

A total of 39 novel cyclotryptamine alkaloid derivatives were prepared from 2-(1*H*-indol-3-yl) acetonitrile, and their antibacterial activities against six plant pathogenic fungi were tested. The results revealed that most of the target compounds had good antibacterial activity against plant pathogenic fungi, and a few compounds showed significant antibacterial activity. Among them, b2 displayed the broadest and most potent activity, which provides directions for further development of potential lead compounds for antifungal drugs.

## Figures and Tables

**Table 1:** MIC values of compounds against plant pathogenic fungi.

Compd.	*MIC* (µg mL^−1^)					
	*S. s.*	*A. s.*	*V. d.*	*F. o.*	*W. p.*	*C. l.*
**a1**	125.00	62.50	250.0	125.00	125.00	125.00
**a2**	125.00	62.50	62.50	125.00	-	31.30
**a3**	31.30	31.30	62.50	62.50	62.50	62.50
**a4**	31.30	62.50	31.30	125.00	31.30	125.00
**a5**	62.50	62.50	15.60	31.30	125.00	62.50
**a6**	250.00	250.00	62.50	-	62.50	62.50
**a7**	125.00	250.00	-	-	62.50	125.00
**a8**	62.50	125.00	125.00	250.00	250.00	-
**a9**	125.00	62.50	125.00	62.50	62.50	31.30
**a10**	31.30	62.50	125.00	-	62.50	125.00
**a11**	62.50	125.00	125.00	62.50	31.30	31.30
**a12**	250.00	125.00	62.50	125.00	31.30	31.30
**a13**	62.50	62.50	31.30	31.30	15.60	125.00
**a14**	250.00	-	-	-	62.50	-
**a15**	31.30	31.30	62.50	31.30	62.50	125.00
**a16**	62.50	62.50	31.30	250.00	125.00	62.50
**a17**	62.50	62.50	31.30	-	-	125.00
**a18**	31.30	125.00	15.60	250.00	125.00	62.50
**a19**	31.30	62.50	125.00	-	62.50	125.00
**a20**	250.00	250.00	125.00	-	-	62.50
**b1**	31.30	15.60	31.30	31.30	62.50	62.50
**b2**	1.90	1.90	3.90	-	31.30	250.00
**b3**	62.50	125.00	31.30	15.60	31.30	31.3
**b4**	3.90	1.90	62.50	3.90	31.30	7.80
**b5**	15.60	7.80	31.30	3.90	31.30	62.50
**b6**	1.90	1.90	-	-	-	500.00
**b7**	125.00	62.50	62.50	250.00	31.30	62.50
**b8**	62.50	125.00	125.00	62.50	31.30	31.30
**b9**	125.00	125.00	62.50	-	-	31.30
**b10**	7.80	1.90	1.90	-	-	125.00
**b11**	31.30	62.50	31.30	15.60	-	31.30
**b12**	62.50	62.50	125.00	250.00	125.00	-
**b13**	3.90	15.60	15.60	31.30	7.80	31.30
**b14**	125.00	62.50	62.50	250.00	-	125.00
**b15**	7.80	1.90	62.50	3.90	62.50	15.60
**b16**	125.00	62.50	62.50	15.60	31.30	31.30
**b17**	250.00	1.90	1.90	-	-	250.00
**b18**	62.50	62.50	-	-	125.00	31.30
**b19**	62.50	31.30	125.00	125.00	62.50	250.00
**C**	7.80	62.50	15.16	62.50	31.30	125.00
**A**	3.90	15.60	125.00	125.00	125.00	62.50

Note: Carbendazim and amphotericin B were used as the positive controls; “-” means no inhibition effect. MIC: Minimal Inhibitory Concentration; *S.s: Sclerotinia sclerotiorum; A.s: Altenaria solani; V.d: Verticillium dahliae; F. o.: Fusarium oxysporum; W. p.: Walnut pythium; C. l.: Curvularia lunata;* C: Carbendazim; A: Amphotericin B.

## Data Availability

Data is contained within the article or the Supplementary Materials file.

## Supplementary Materials

The following supporting information can be downloaded at: https://www.mdpi.com/article/10.3390/molecules28062617/s1, ^1^H and ^13^C NMR Spectra.

## Sample Availability

Samples of the compounds are available from the authors.
