# Supplementary material for: Synthesis and Structure–Activity Analysis of Novel Potential Antifungal Cyclotryptamine Alkaloid Derivatives
Source: Molecules. 2023 Mar 13;28(6):2617. doi: 10.3390/molecules28062617 (PMC10053776; doi:10.3390/molecules28062617)
Supplement: Supplementary file 1 [file molecules-28-02617-s001.zip › molecules-2234654-supplementary.pdf]

SUPPLEMENTARY MATERIAL

Synthesis and Structure–Activity Analysis of Novel Potential Antifungal Cyclotryptamine Alkaloid Derivatives

Yazhou Yang †, Yujie Zhou †, Qiaoju Jiang, Yi Tan, Wenbin Wu, Ke Han, Rui Zhu and Shaojun Zheng *

School of Environmental and Chemical Engineering, Jiangsu University of Science and Technology, Zhenjiang 212003, China; ads4cn@gmail.com (Y.Y.); zyj33662023@163.com (Y.Z.); 15136801650@163.com (Q.J.); anyiris2020@163.com (Y.T.); b752526149@163.com (W.W.); h15289369325@163.com (K.H.); rzhu508@163.com (R.Z.)

* Correspondence: sz281cam@just.edu.cn; Tel.: +86-0511-8561-6983

† These authors contributed equally to this work.

^{1}H and ^{13}C NMR Spectra

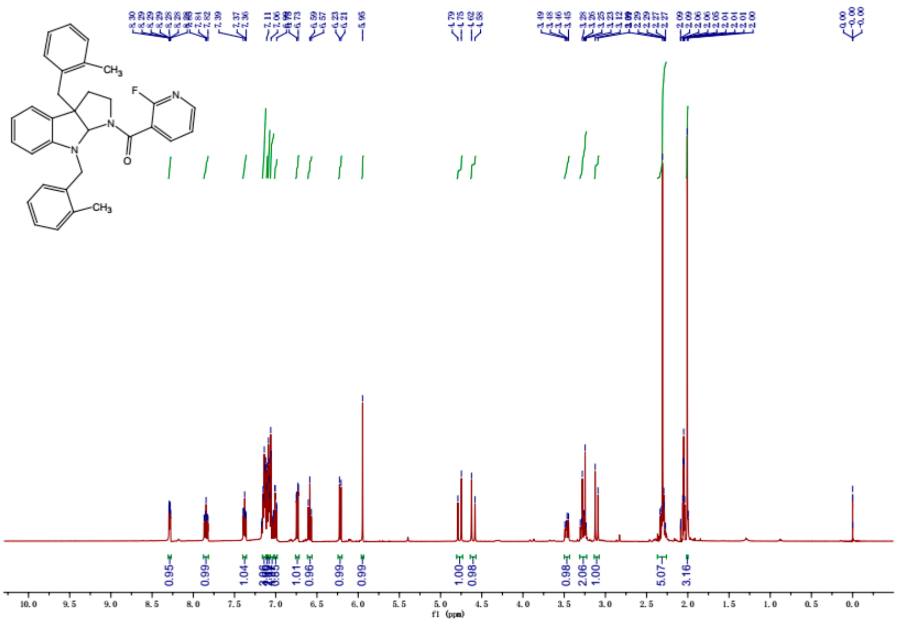

Figure S1. ^{1}H-NMR spectroscopic data for compound **a1**.

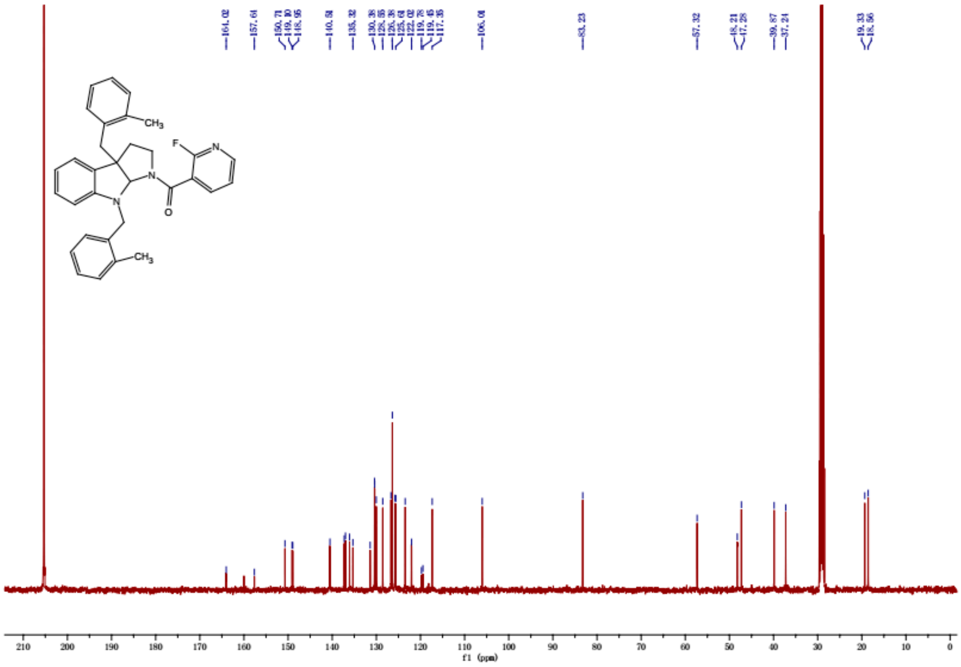

Figure S2. ^{13}C-NMR spectroscopic data for compound **a1**

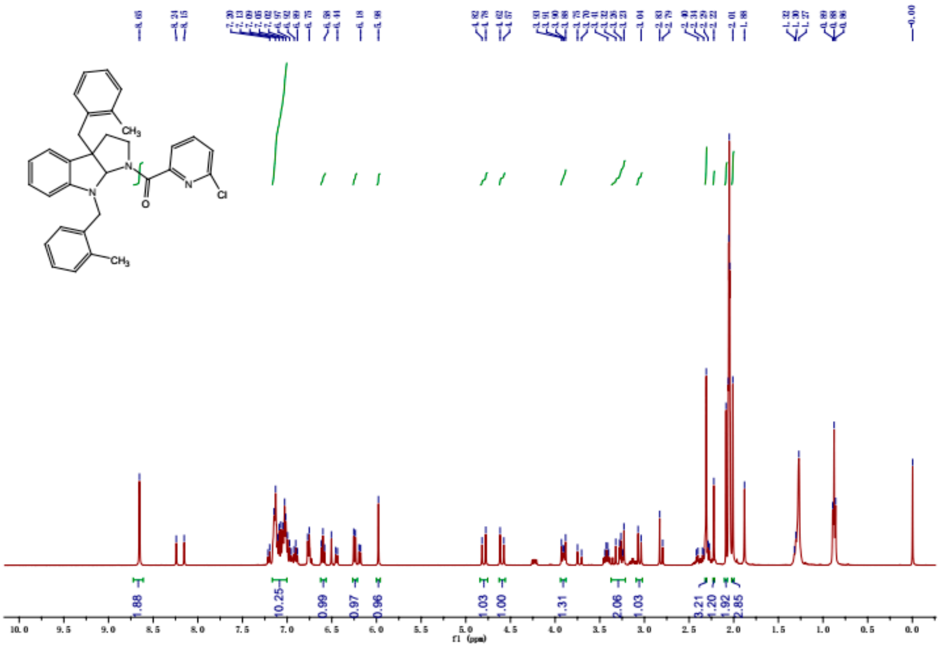

Figure S3. ^{1}H-NMR spectroscopic data for compound **a2**.

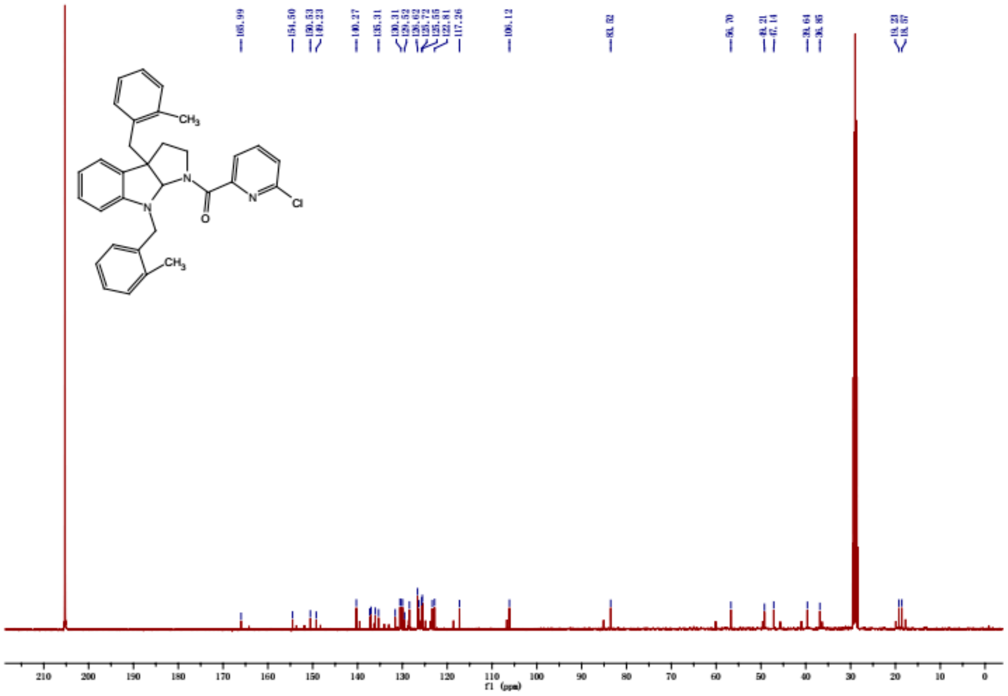

Figure S4. ^{13}C-NMR spectroscopic data for compound **a2**

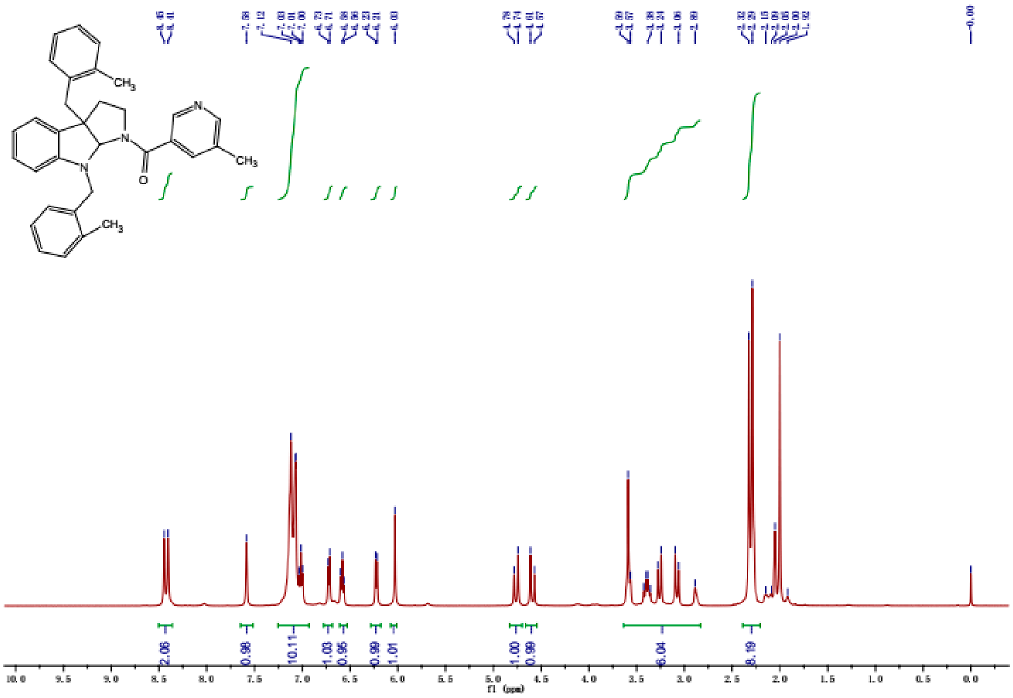

Figure S5. ^{1}H-NMR spectroscopic data for compound **a3**.

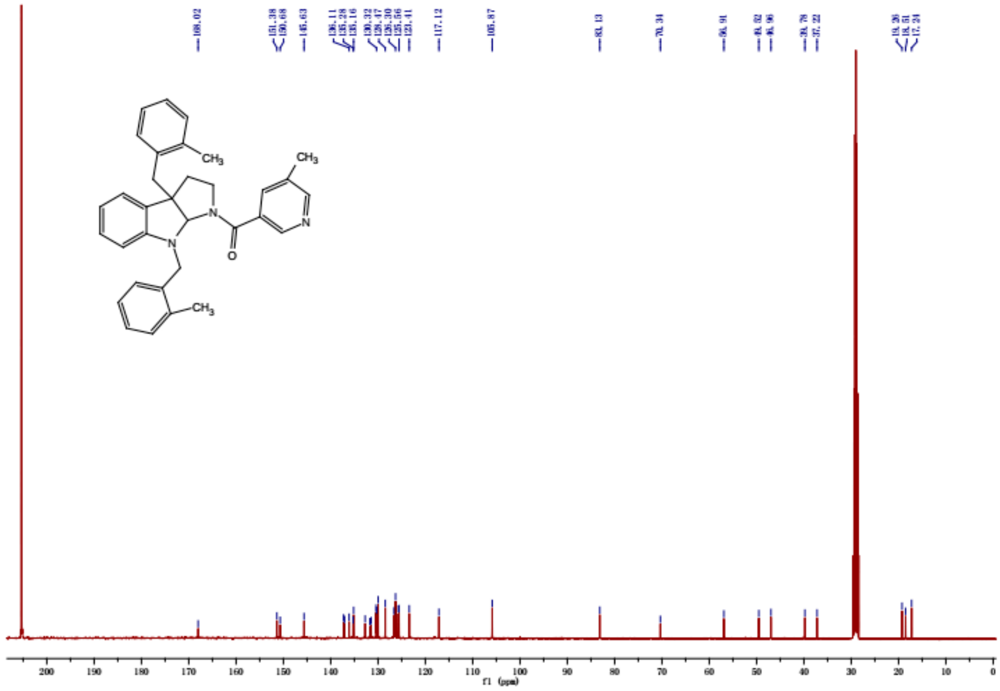

Figure S6. ^{13}C-NMR spectroscopic data for compound **a3**

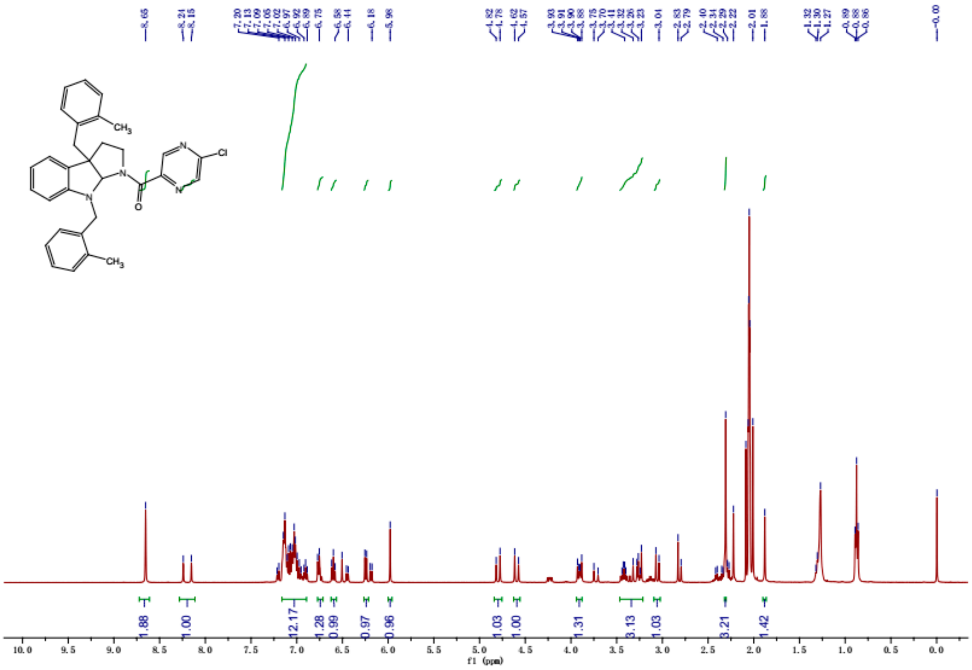

Figure S7. ^{1}H-NMR spectroscopic data for compound **a4**.

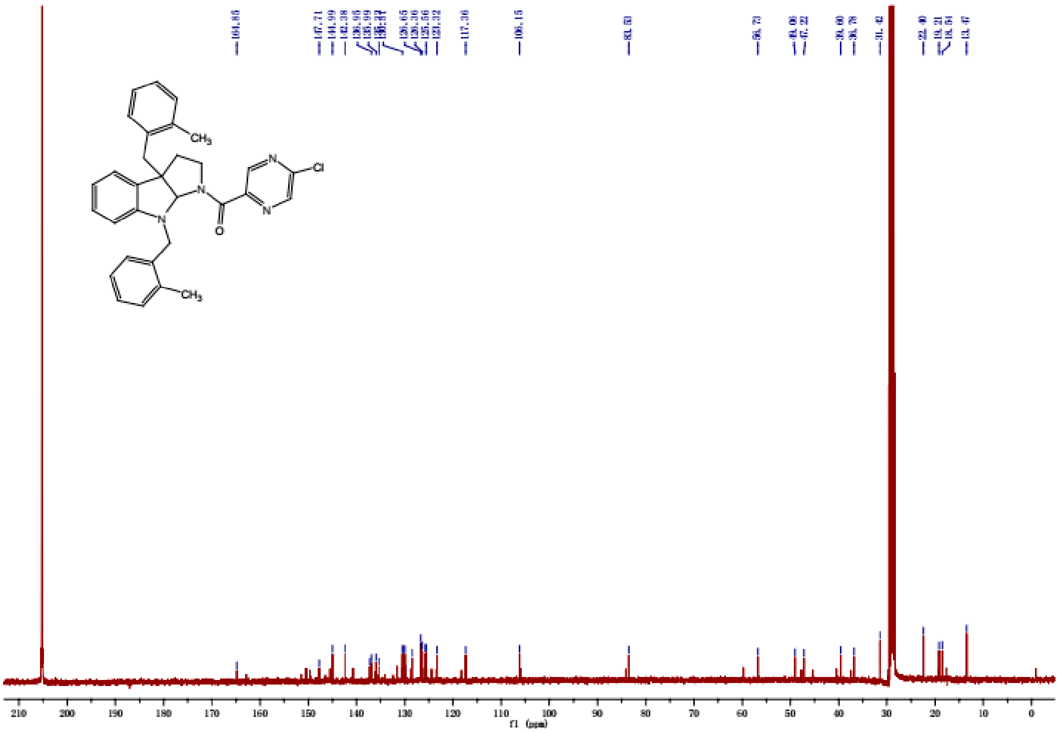

Figure S8. ^{13}C-NMR spectroscopic data for compound **a4**

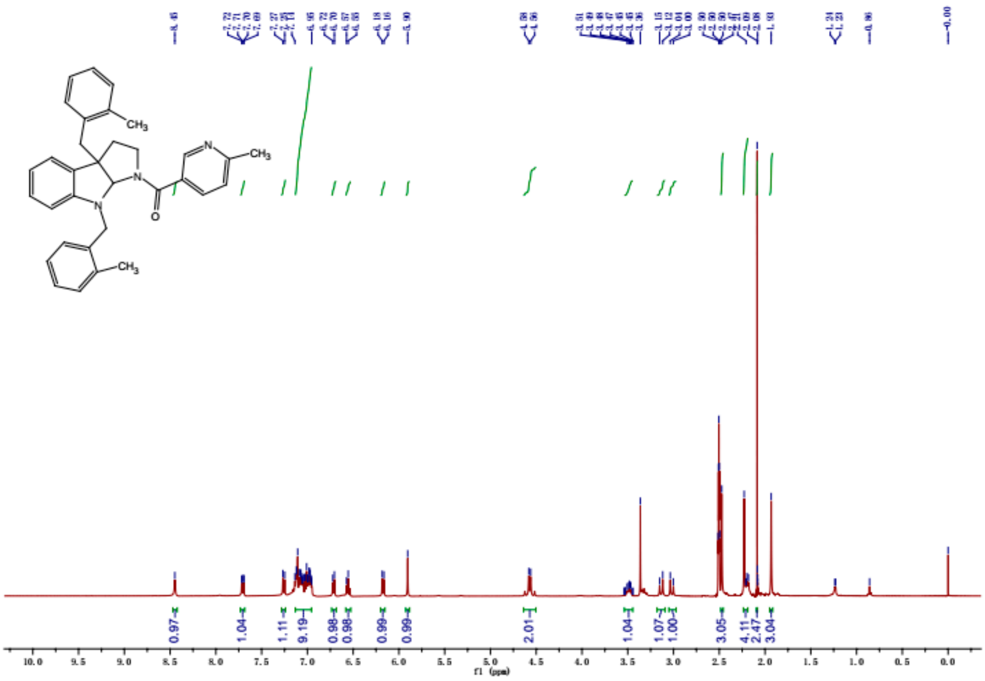

Figure S9. ^{1}H-NMR spectroscopic data for compound **a5**.

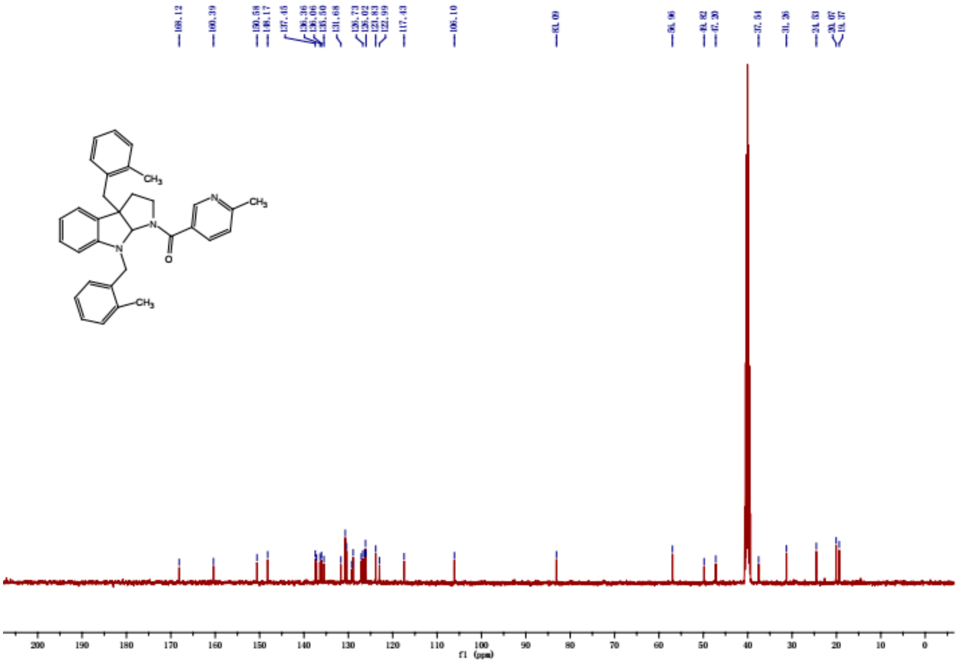

Figure S10. ^{13}C-NMR spectroscopic data for compound **a5**

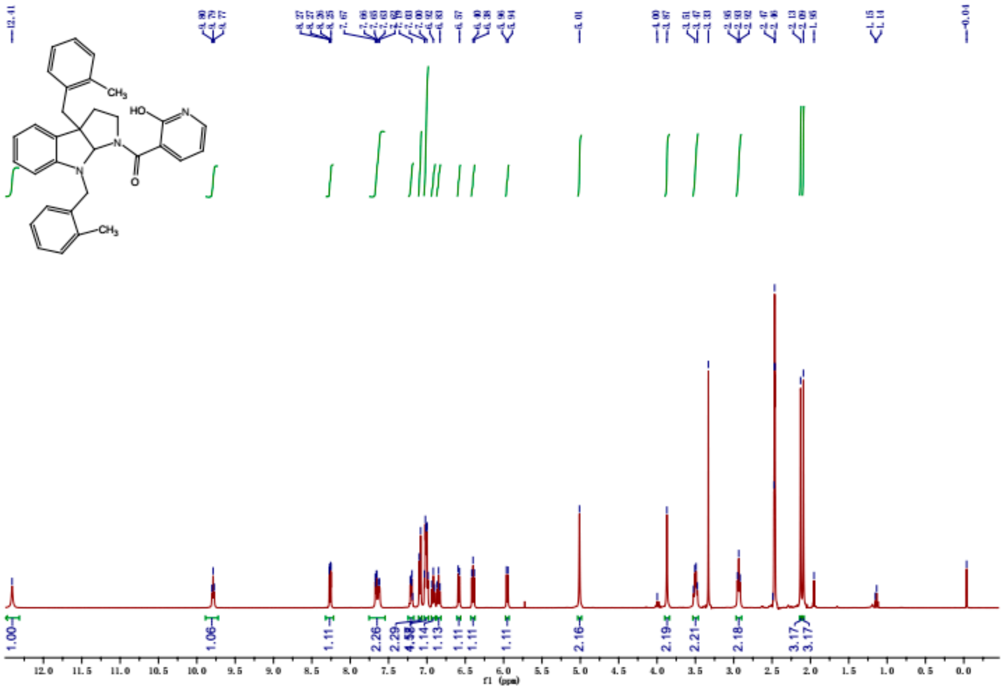

Figure S11. ^{1}H-NMR spectroscopic data for compound **a6**.

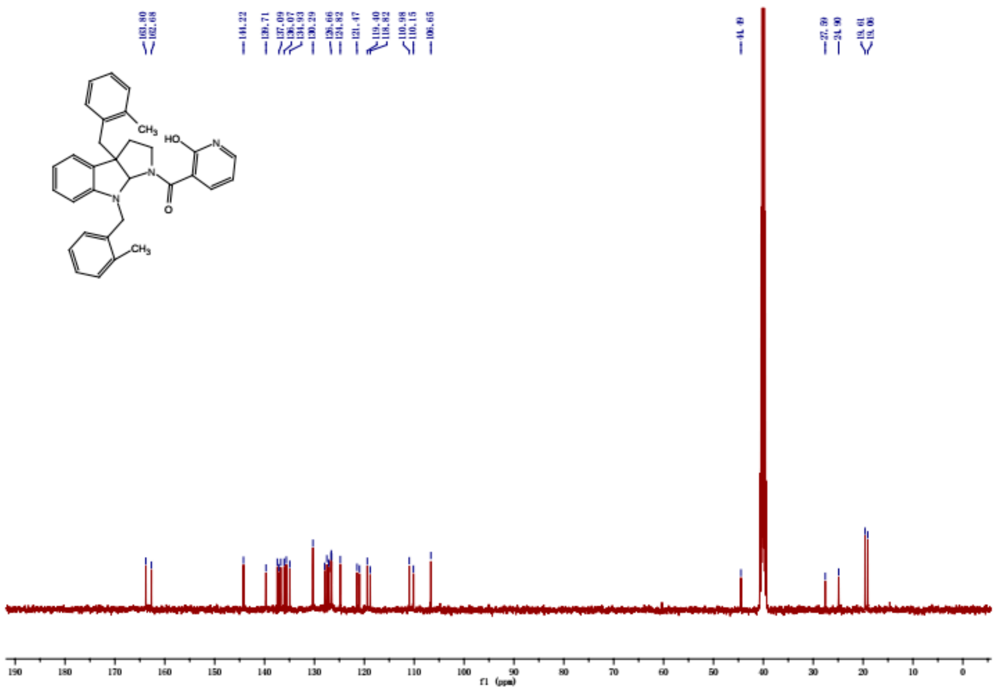

Figure S12. ^{13}C-NMR spectroscopic data for compound **a6**

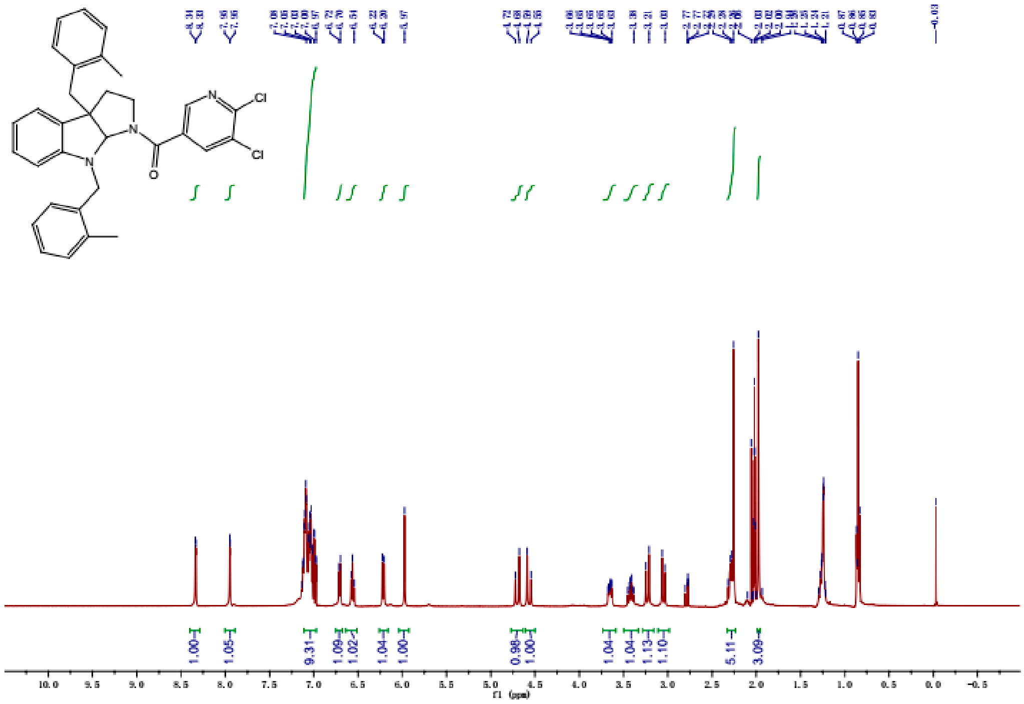

Figure S13. ^{1}H-NMR spectroscopic data for compound **a7**.

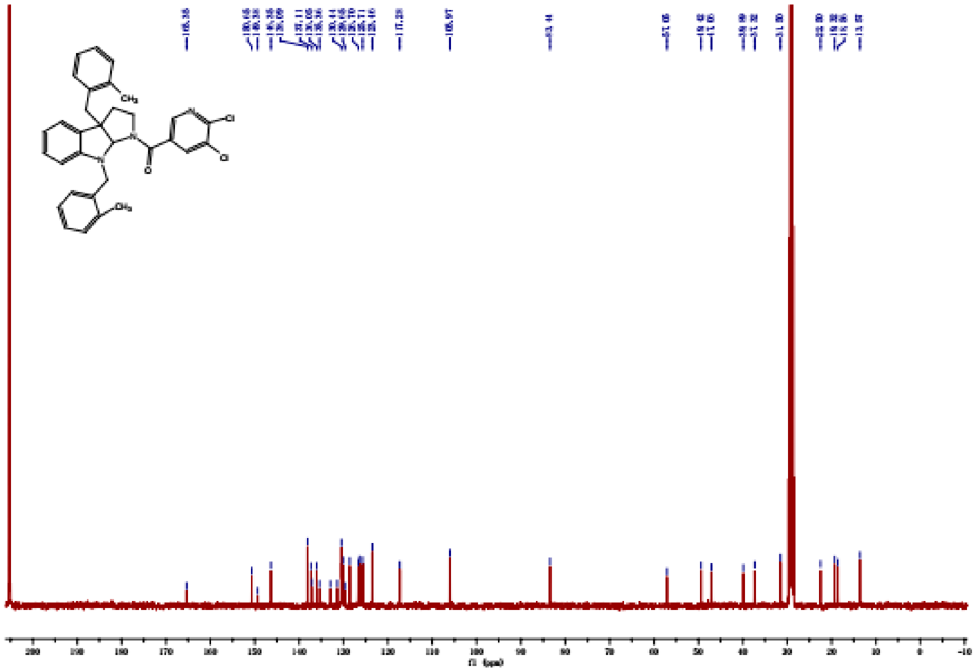

Figure S14. ^{13}C-NMR spectroscopic data for compound **a7**

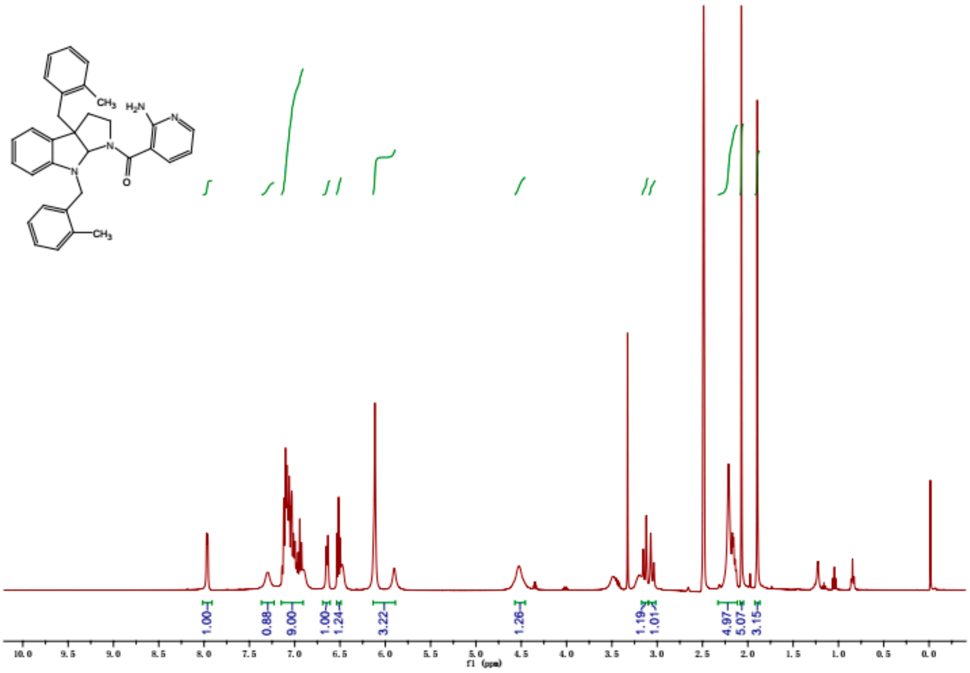

Figure S15. ^{1}H-NMR spectroscopic data for compound **a8**.

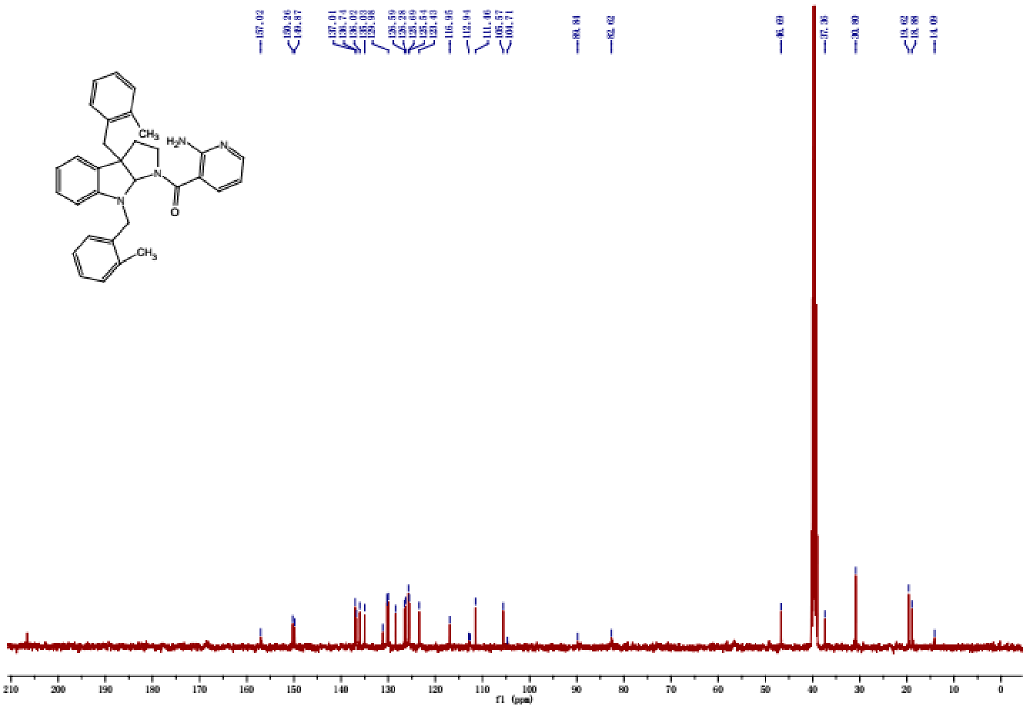

Figure S16. ^{13}C-NMR spectroscopic data for compound **a8**

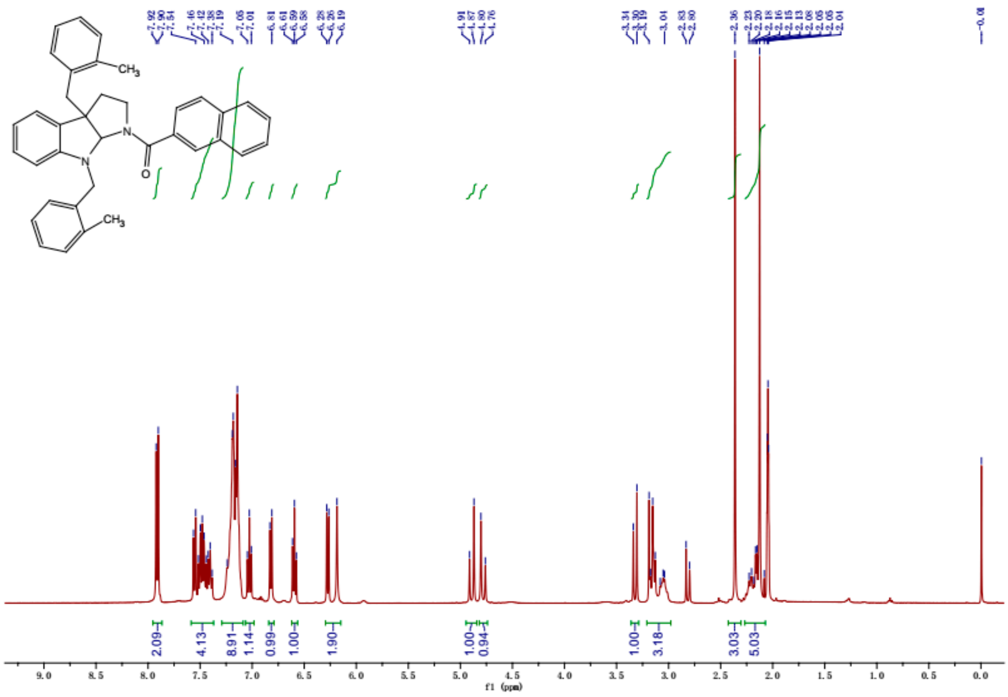

Figure S17. ^{1}H-NMR spectroscopic data for compound **a9**.

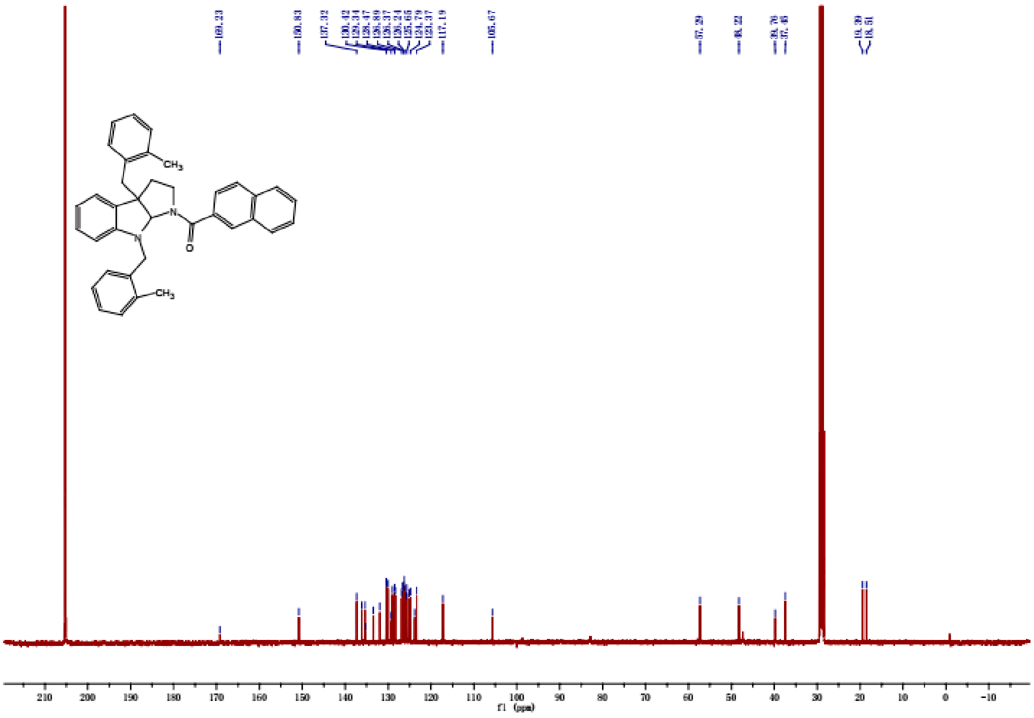

Figure S18. ^{13}C-NMR spectroscopic data for compound **a9**

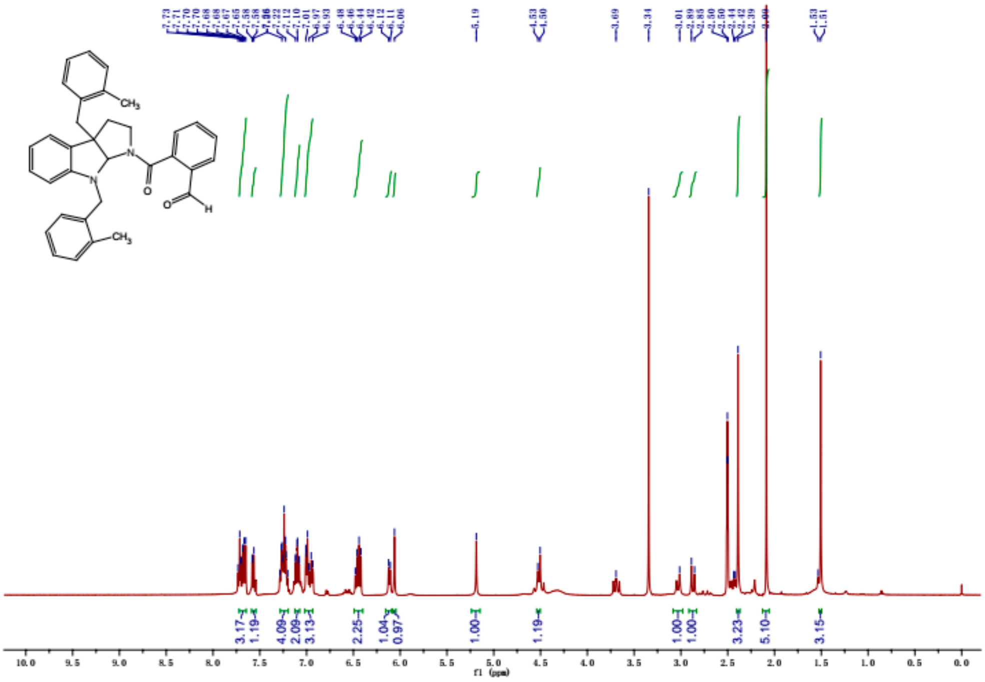

Figure S19. ^{1}H-NMR spectroscopic data for compound **a10**.

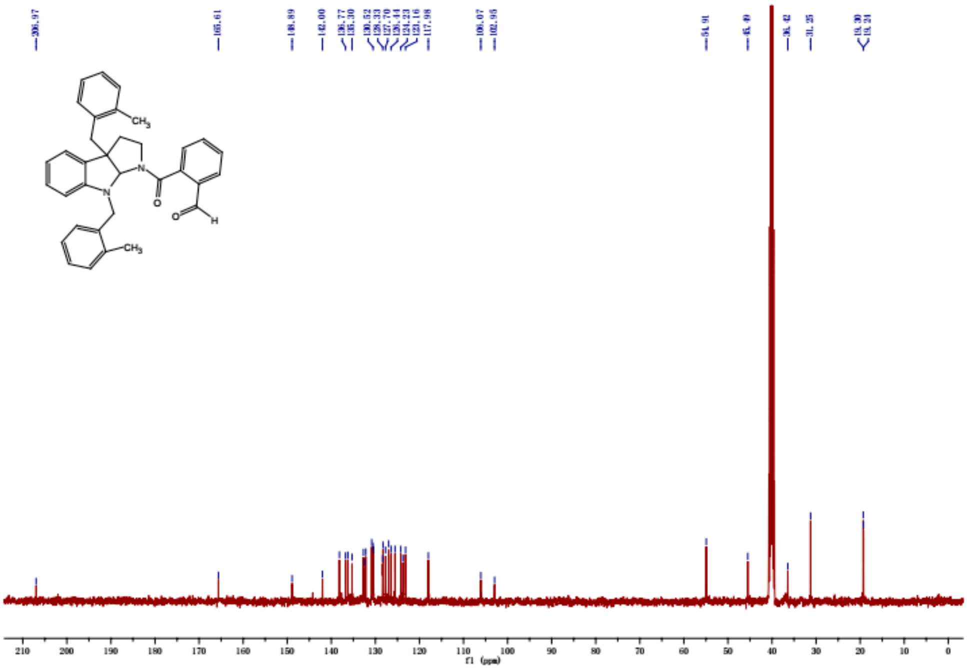

Figure S20. ^{13}C-NMR spectroscopic data for compound **a10**

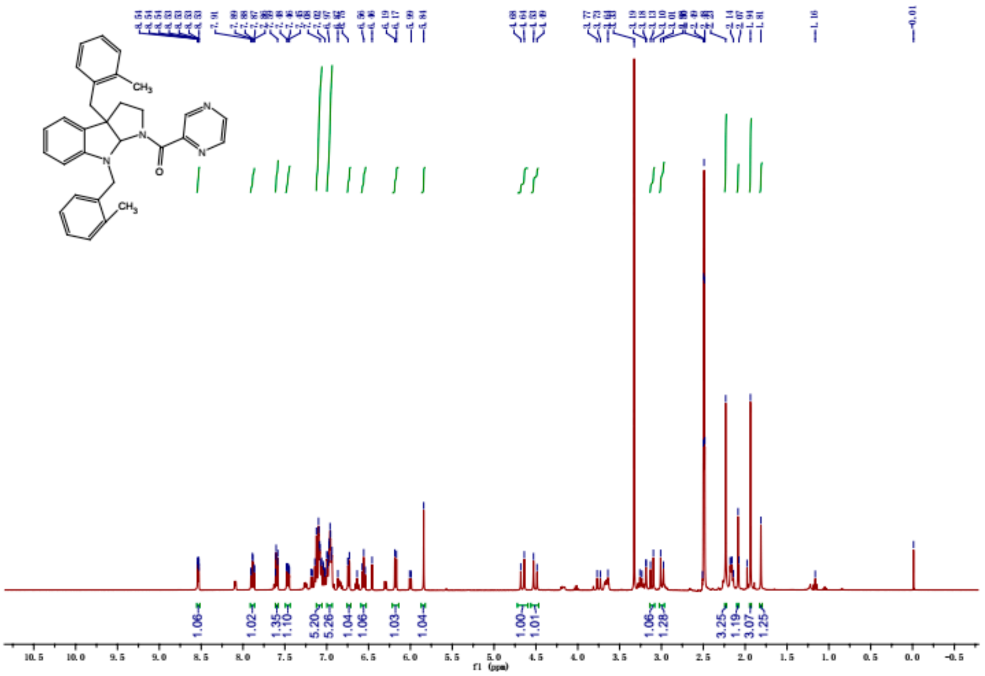

Figure S21. ^{1}H-NMR spectroscopic data for compound **a11**.

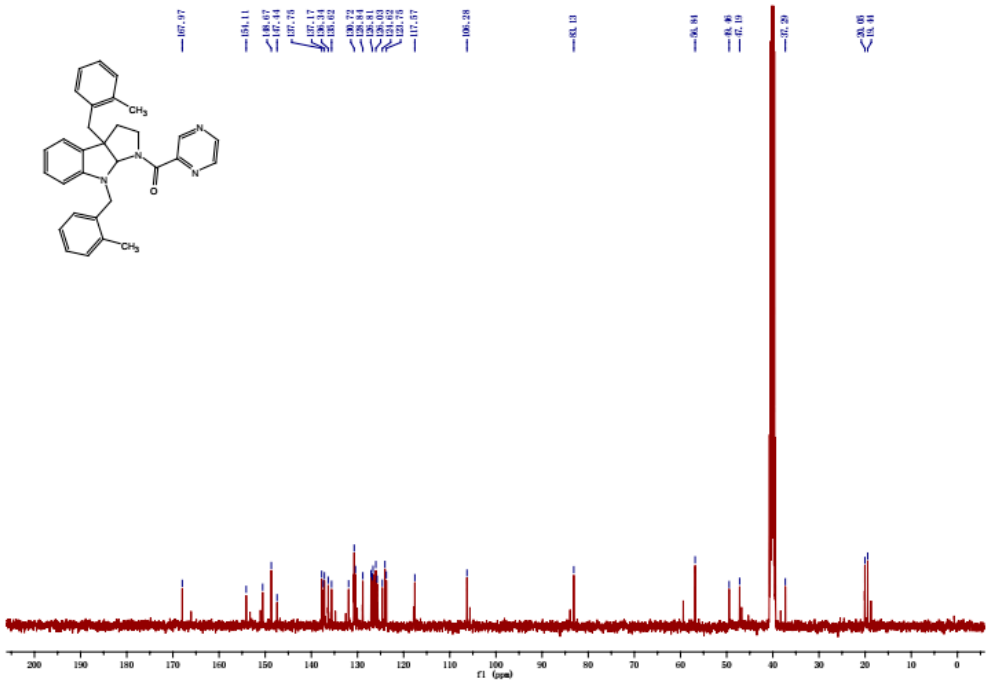

Figure S22. ^{13}C-NMR spectroscopic data for compound **a11**

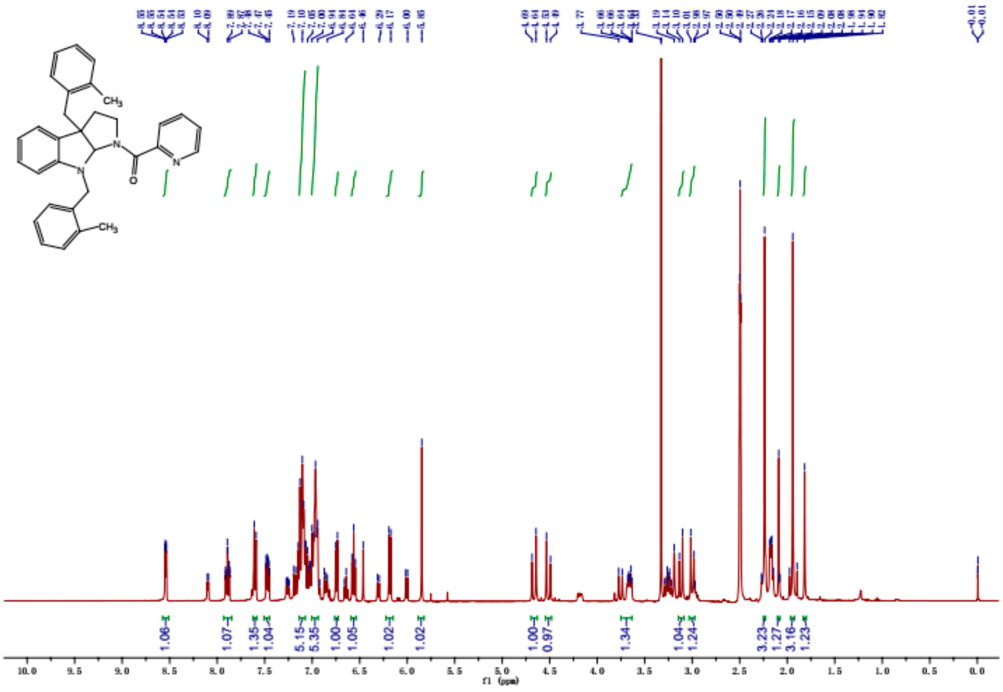

Figure S23. ^{1}H-NMR spectroscopic data for compound **a12**.

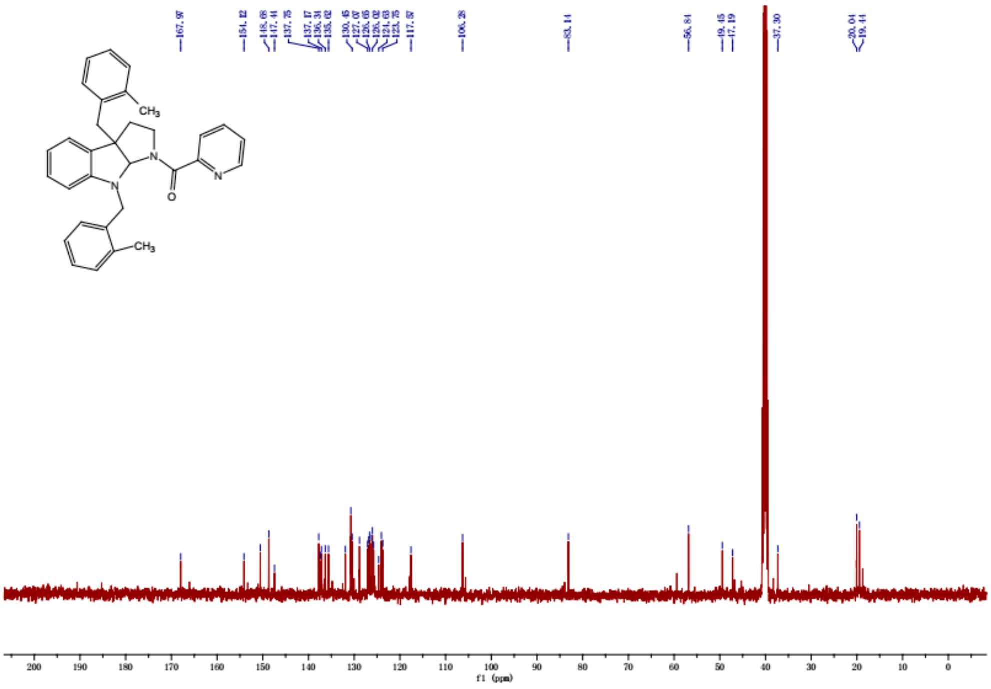

Figure S24. ^{13}C-NMR spectroscopic data for compound **a12**

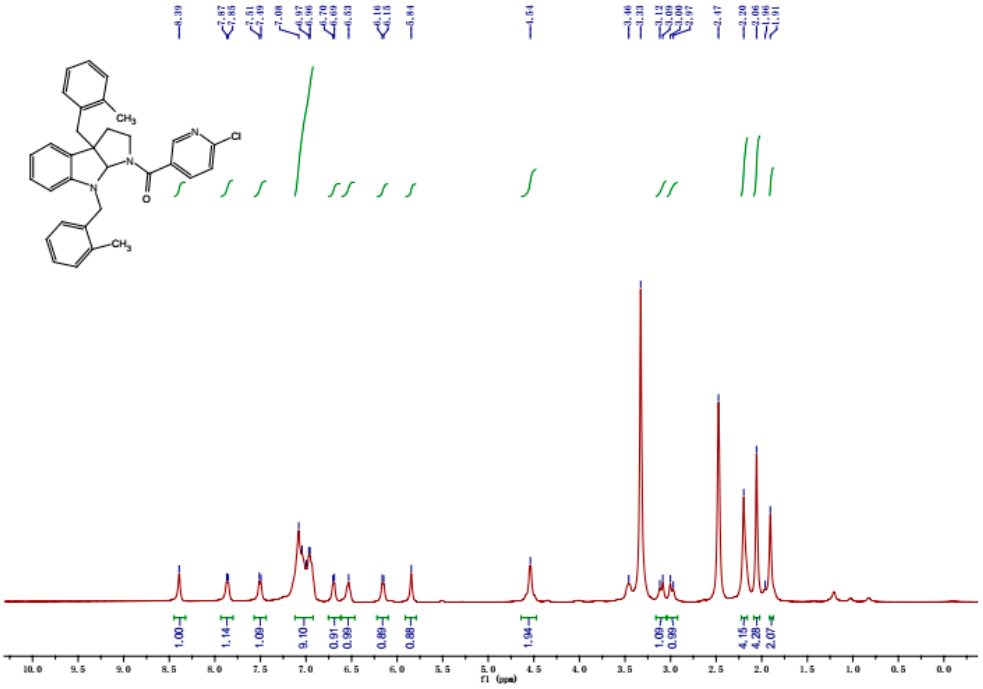

Figure S25. ^{1}H-NMR spectroscopic data for compound **a13**.

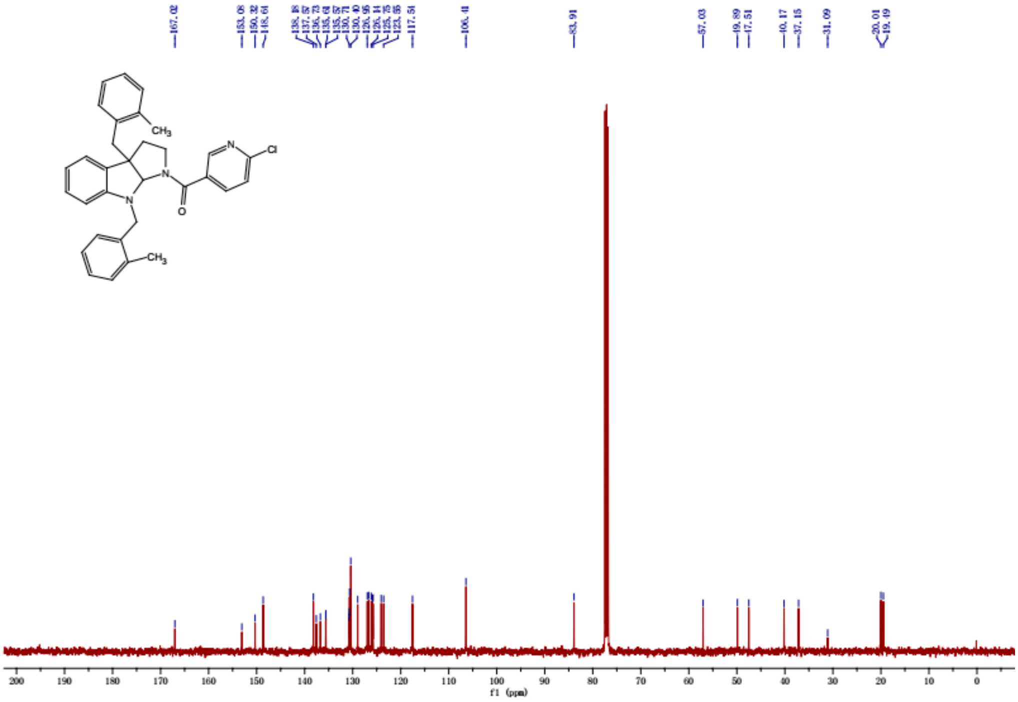

Figure S26. ^{13}C-NMR spectroscopic data for compound **a13**

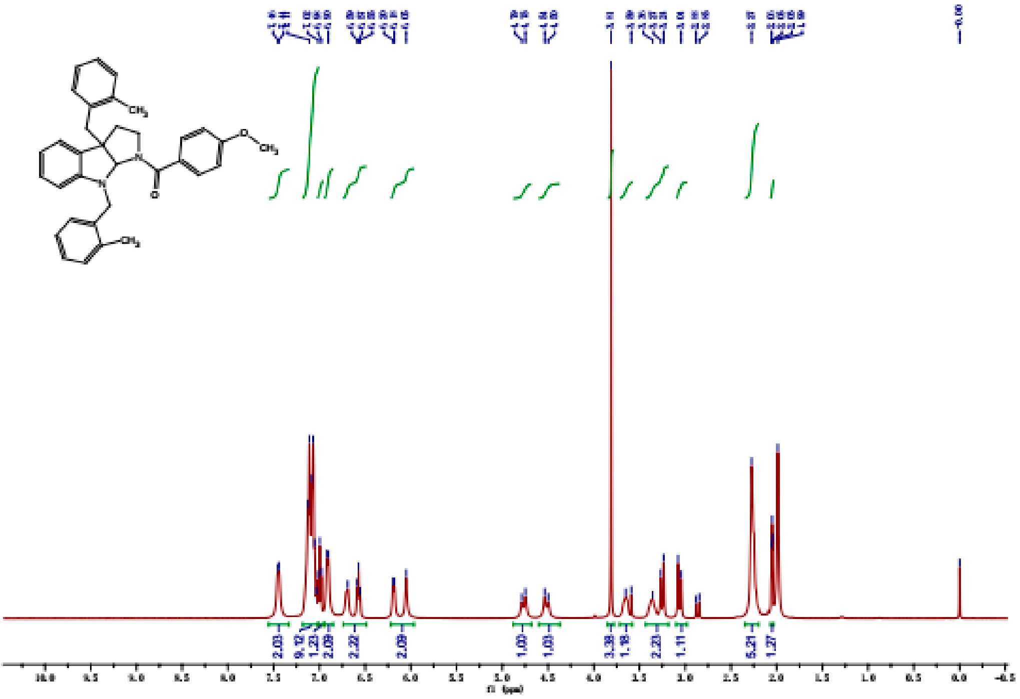

Figure S27. ^{1}H-NMR spectroscopic data for compound **a14**.

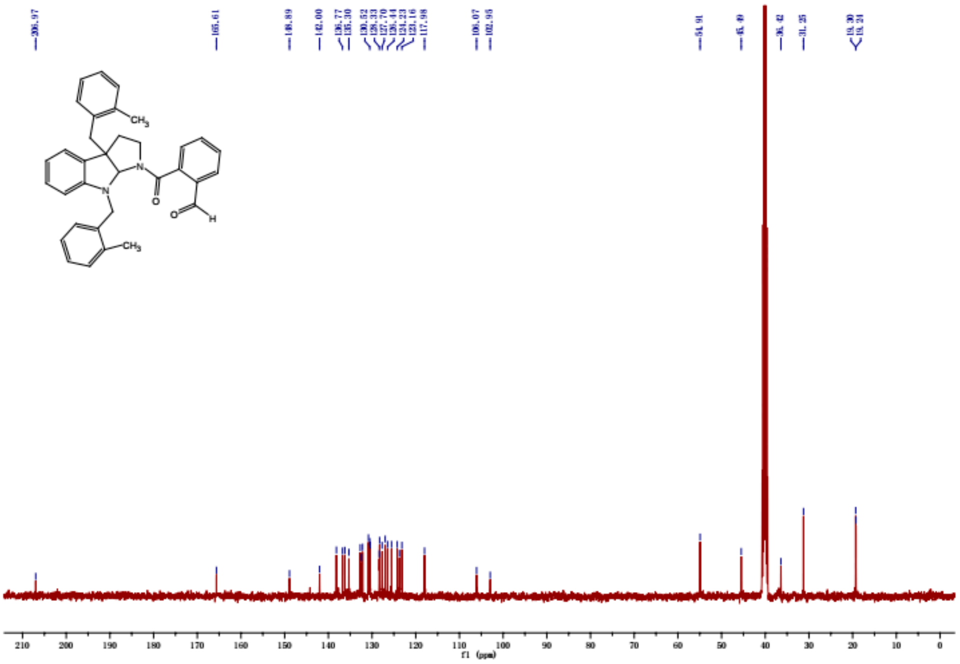

Figure S28. ^{13}C-NMR spectroscopic data for compound **a14**

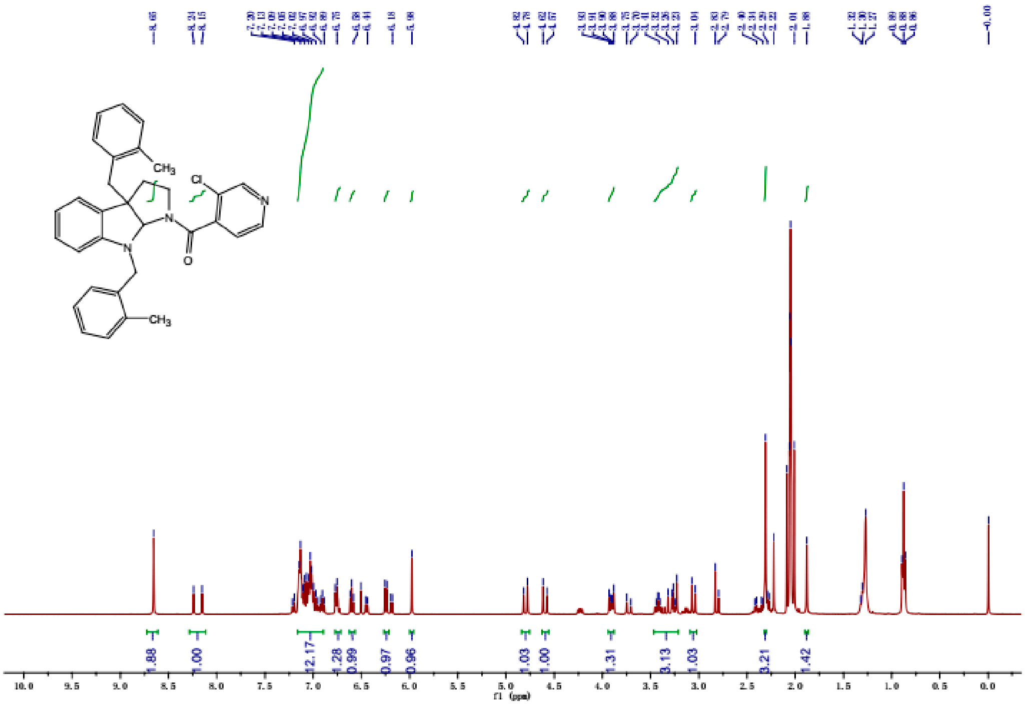

Figure S29. ^{1}H-NMR spectroscopic data for compound **a15**.

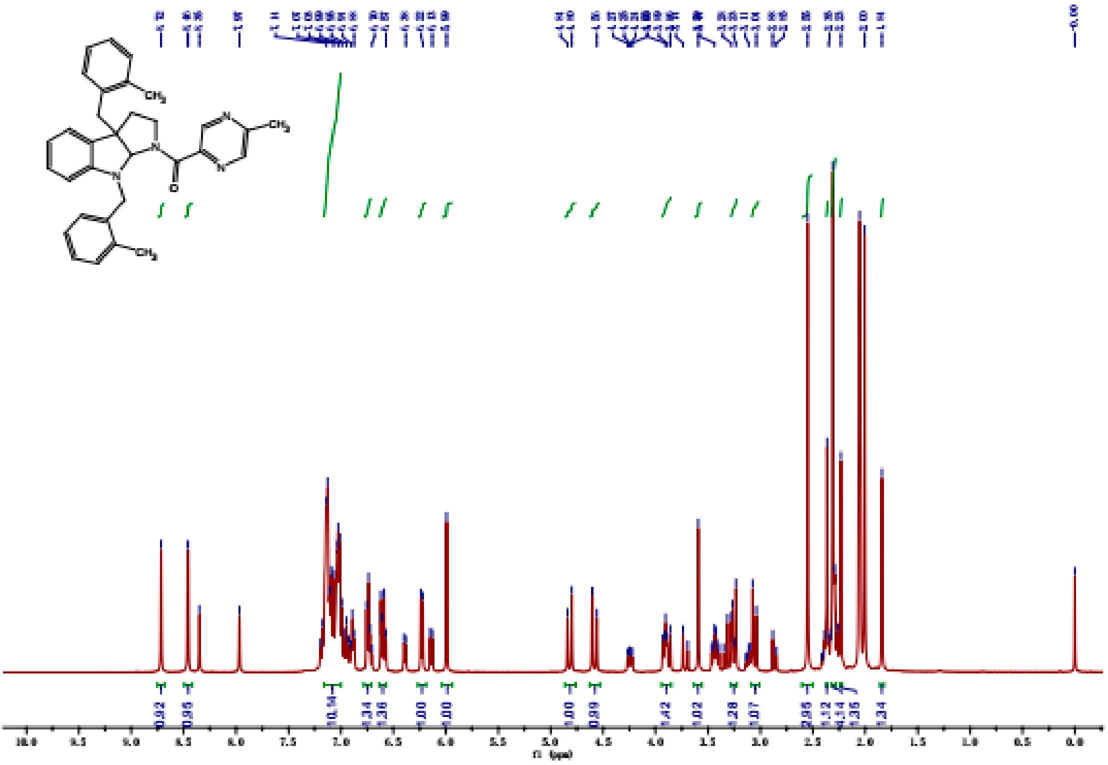

Figure S30. ^{13}C-NMR spectroscopic data for compound **a15**

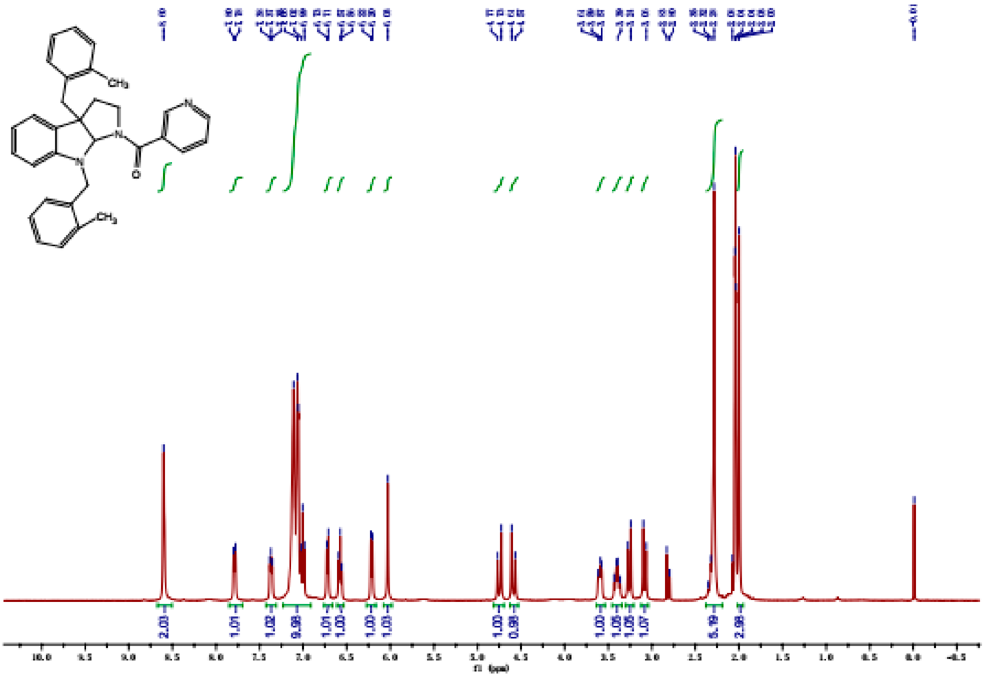

Figure S31. ^{1}H-NMR spectroscopic data for compound **a16**.

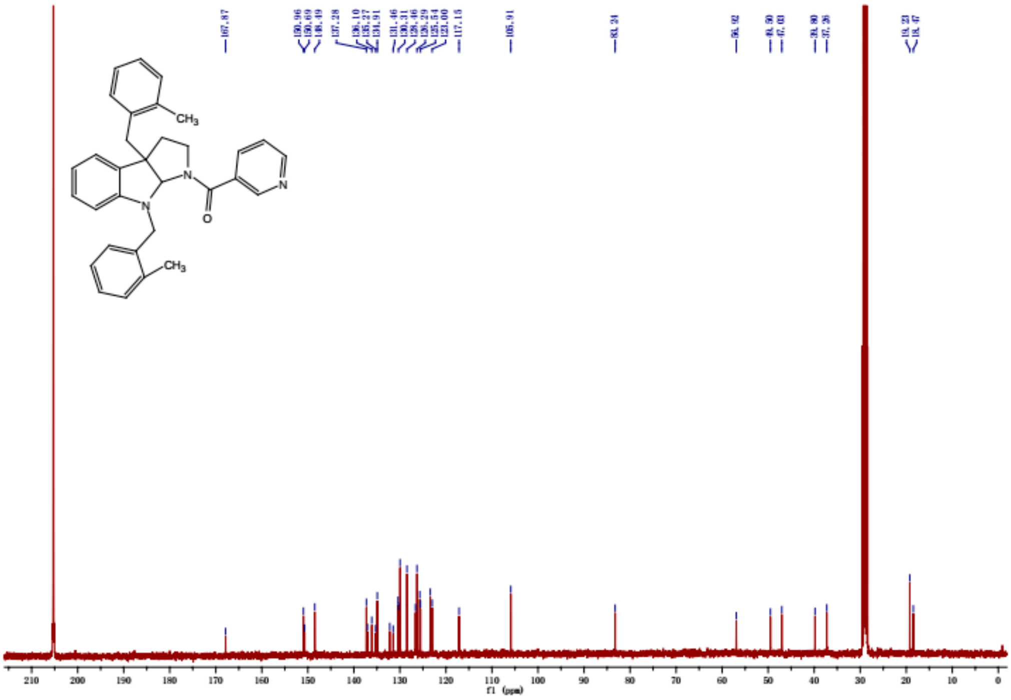

Figure S32. ^{13}C-NMR spectroscopic data for compound **a16**

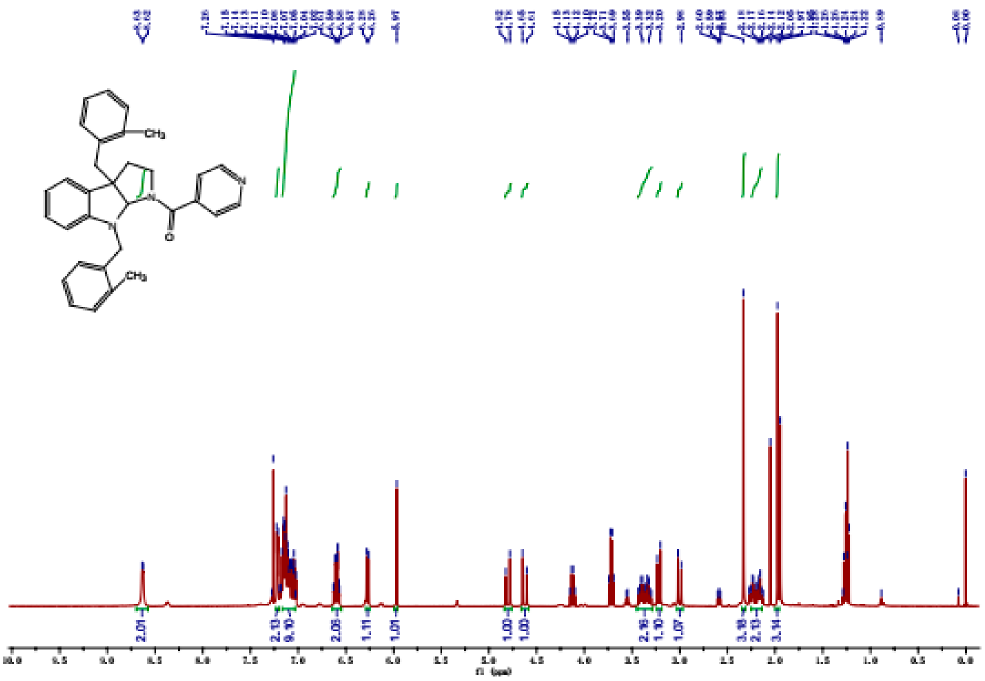

Figure S33. ^{1}H-NMR spectroscopic data for compound **a17**.

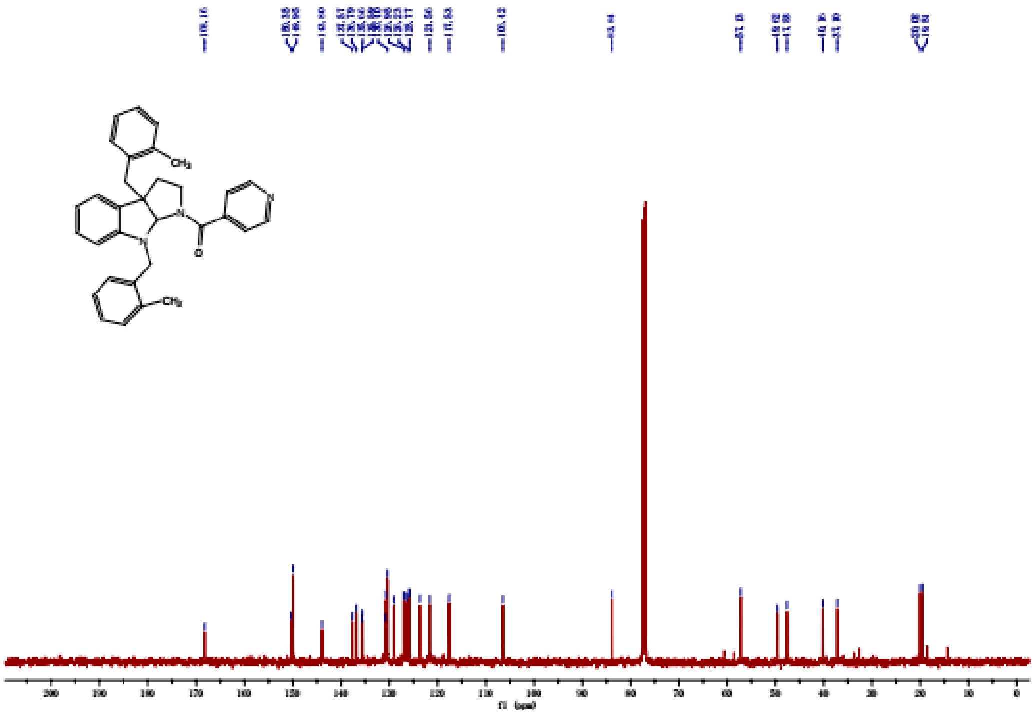

Figure S34. ^{13}C-NMR spectroscopic data for compound **a17**

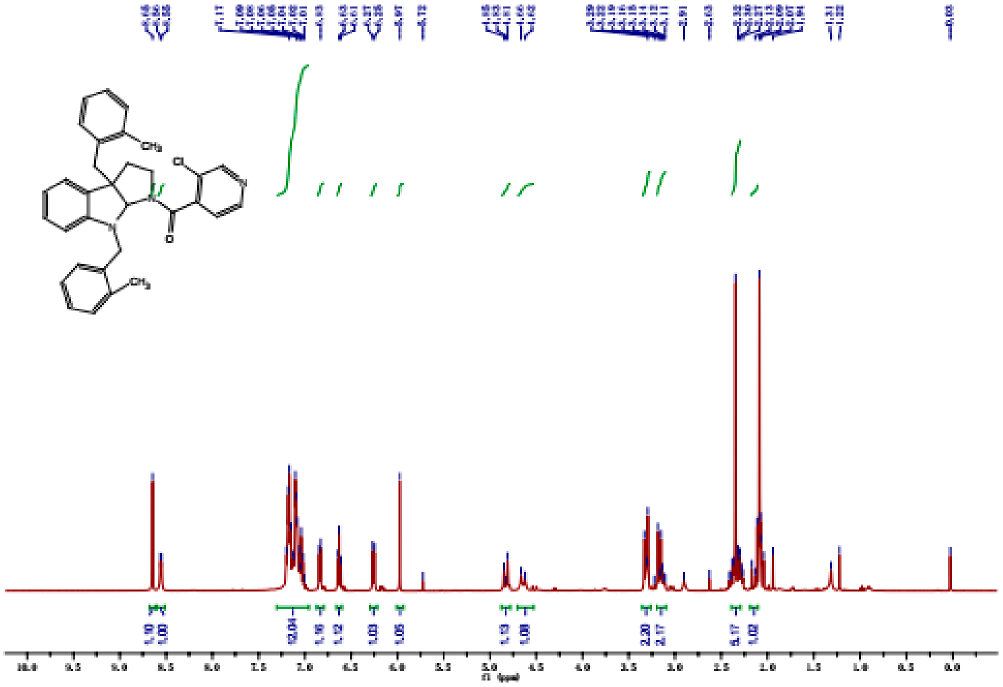

Figure S35. ^{1}H-NMR spectroscopic data for compound **a18**.

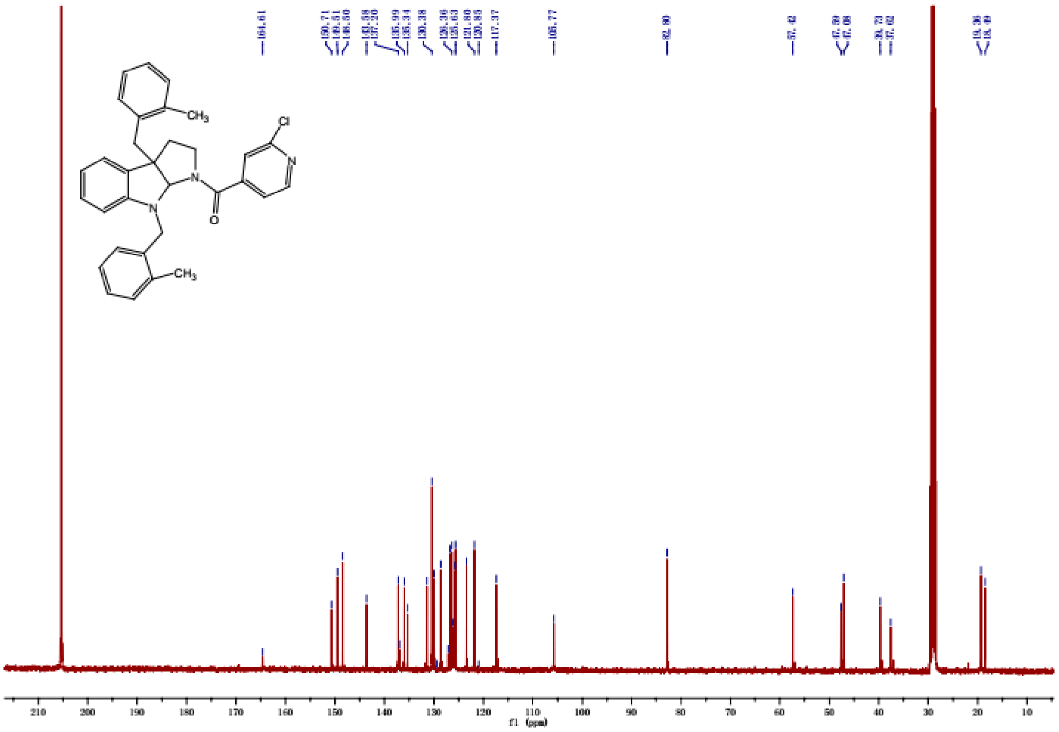

Figure S36. ^{13}C-NMR spectroscopic data for compound **a18**

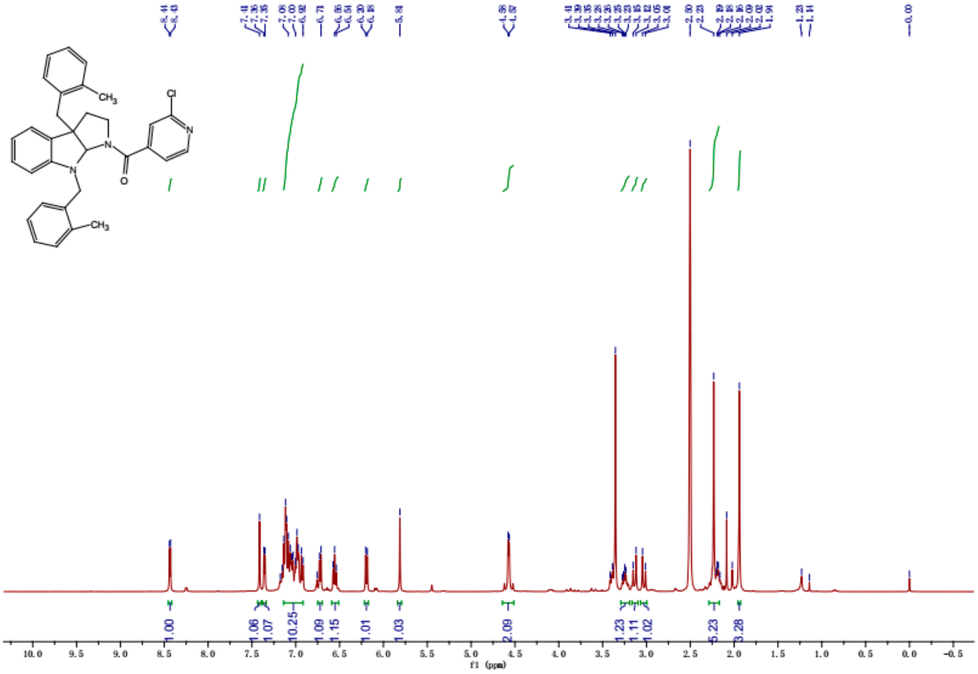

Figure S37. ^{1}H-NMR spectroscopic data for compound **a19**.

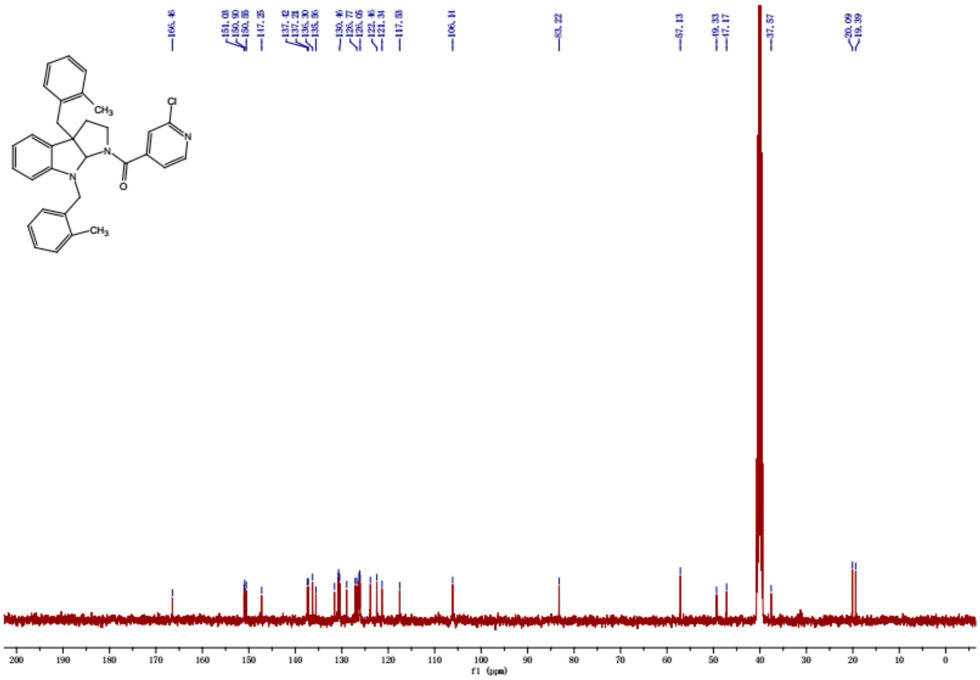

Figure S38. ^{13}C-NMR spectroscopic data for compound **a19**

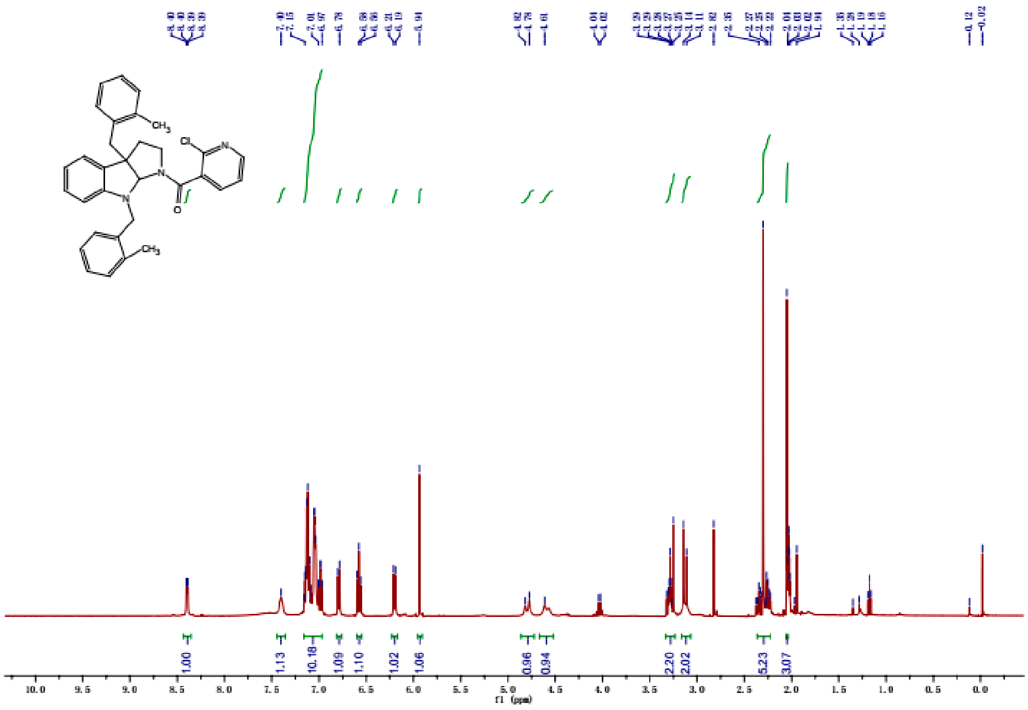

Figure S39. ^{1}H-NMR spectroscopic data for compound **a20**.

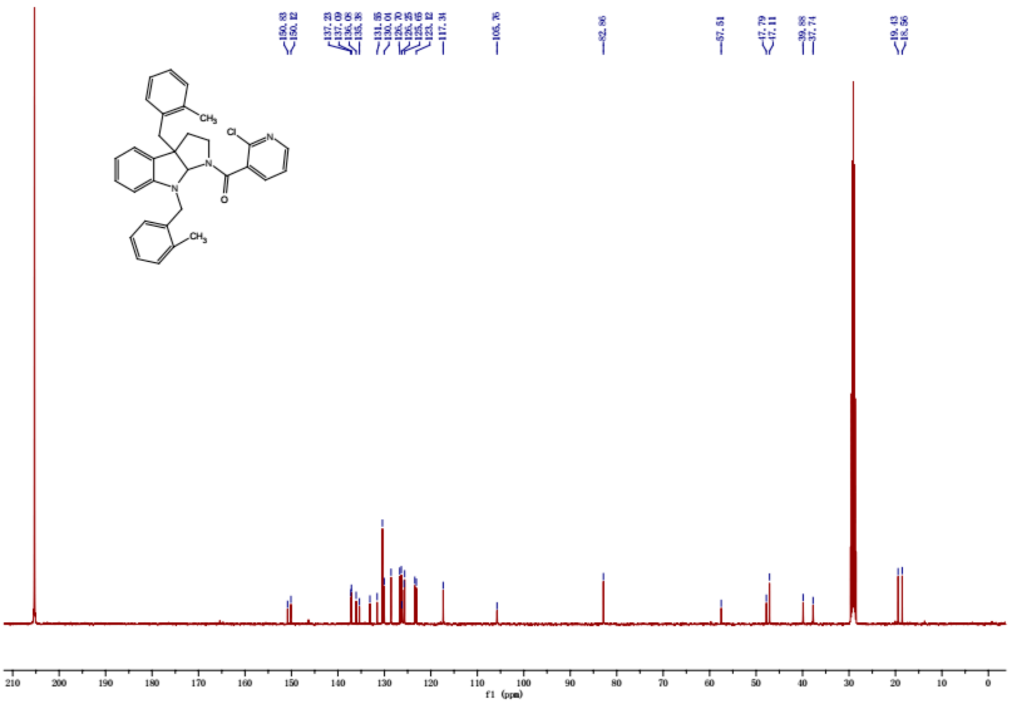

Figure S40. ^{13}C-NMR spectroscopic data for compound **a20**

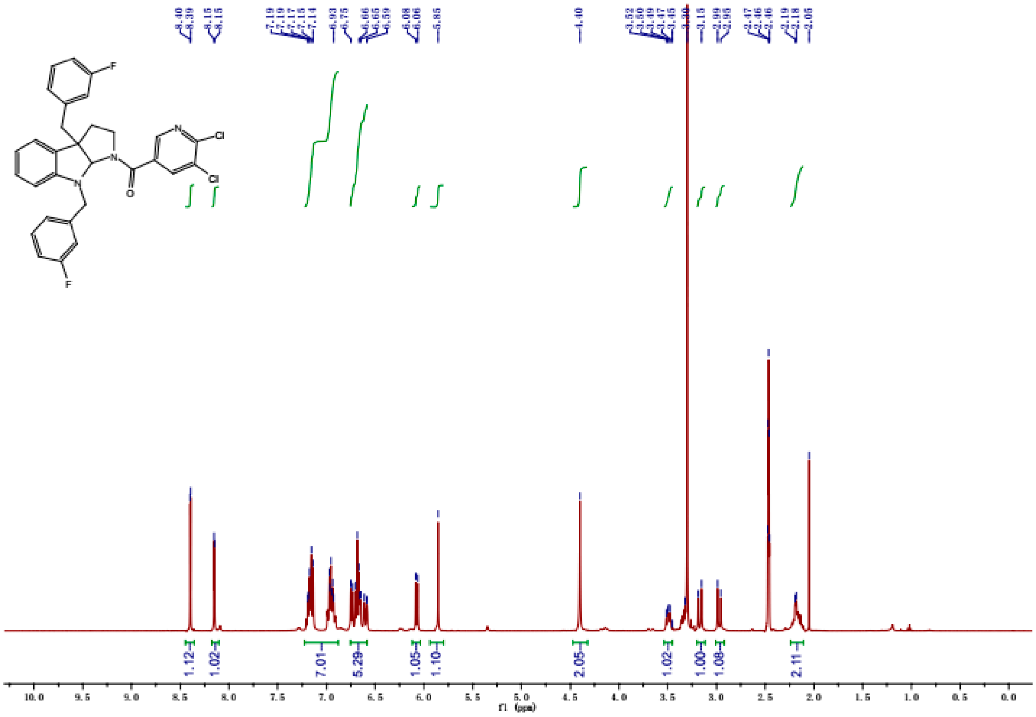

Figure S41. ^{1}H-NMR spectroscopic data for compound **b1**.

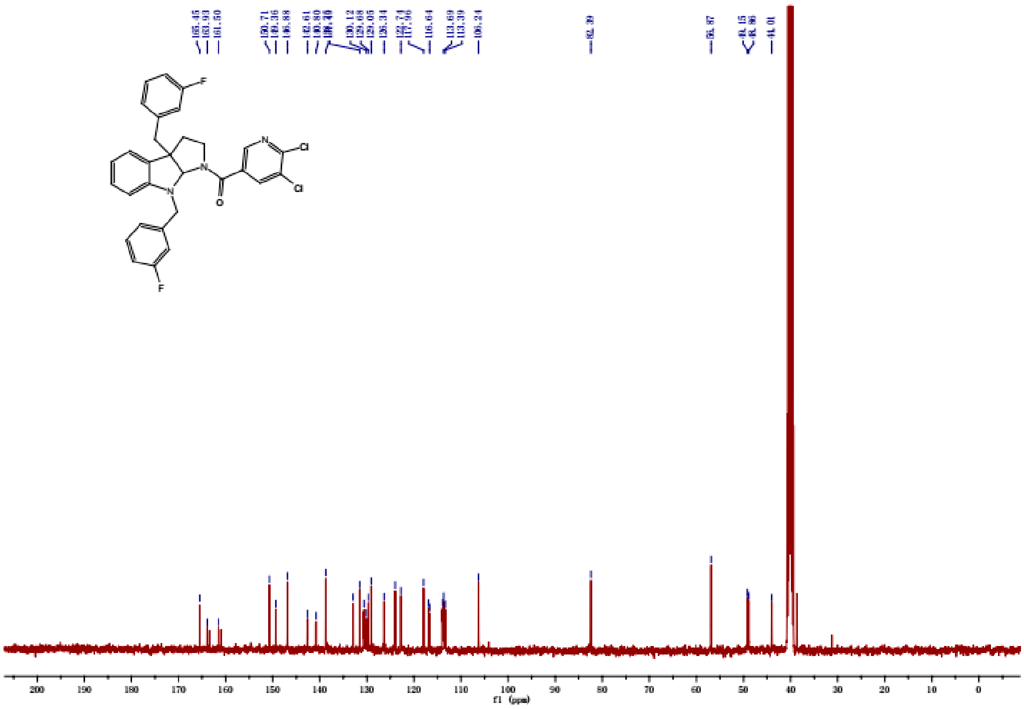

Figure S42. ^{13}C-NMR spectroscopic data for compound **b1**

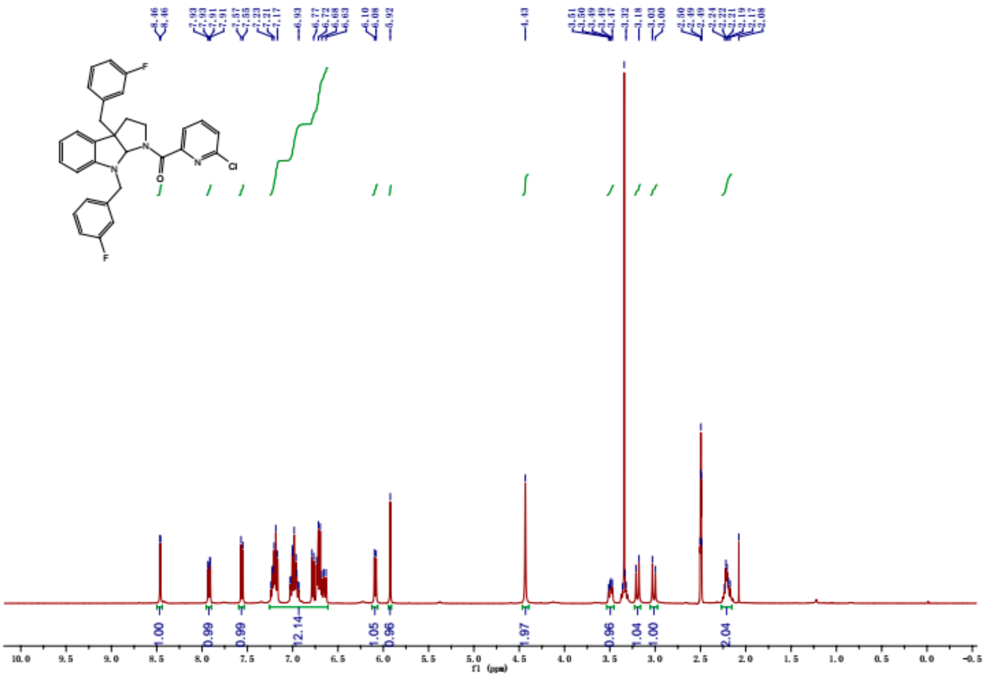

Figure S43. ^{1}H-NMR spectroscopic data for compound **b2**.

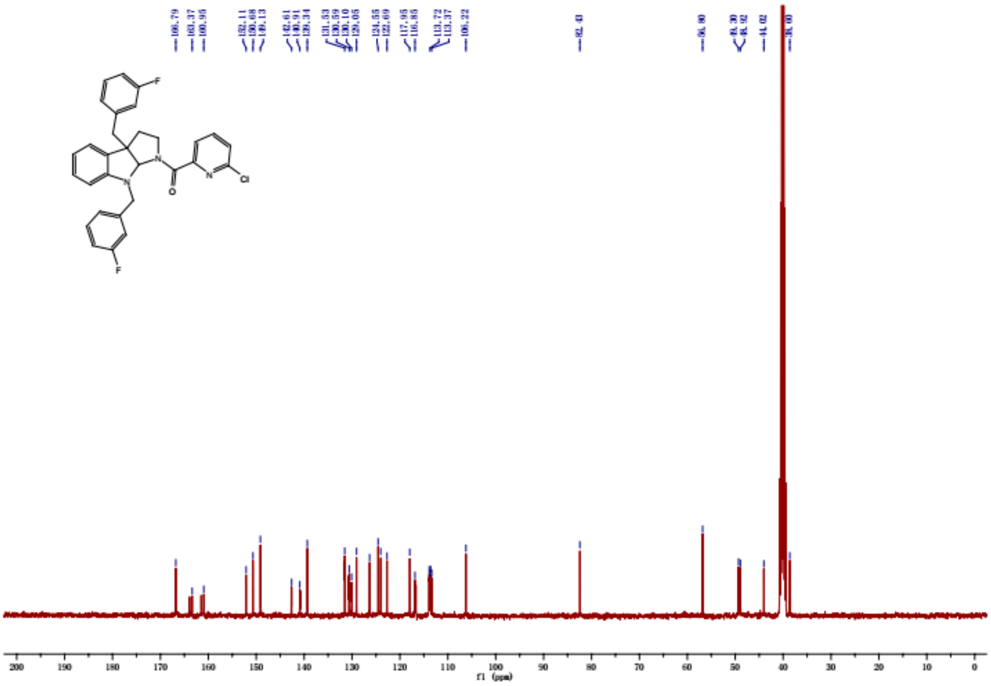

Figure S44. ^{13}C-NMR spectroscopic data for compound **b2**

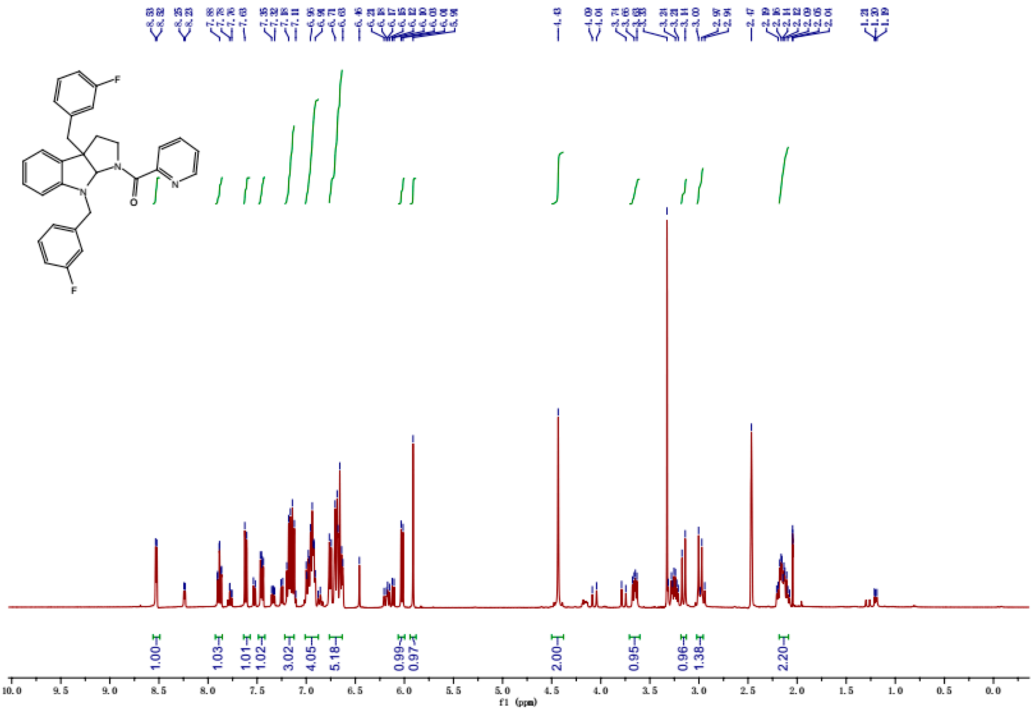

Figure S45. ^{1}H-NMR spectroscopic data for compound **b3**.

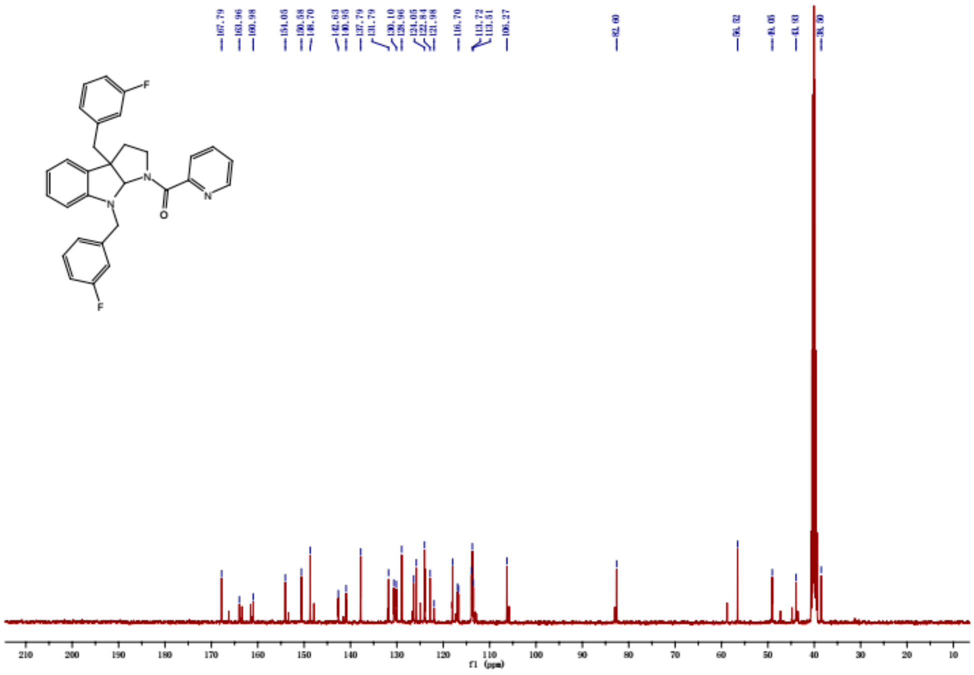

Figure S46. ^{13}C-NMR spectroscopic data for compound **b3**

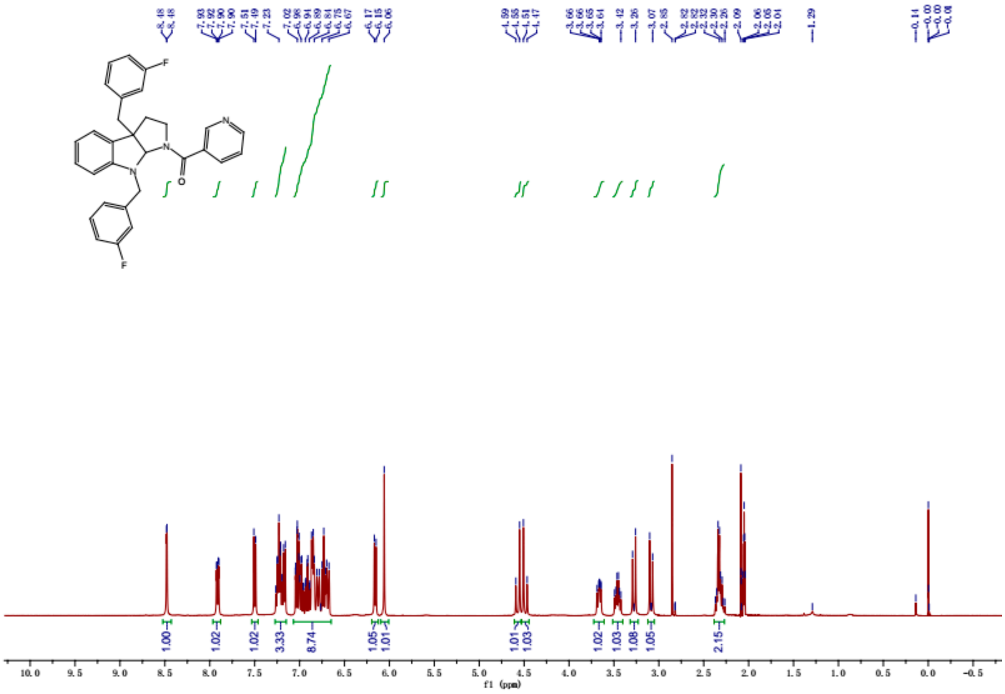

Figure S47. ^{1}H-NMR spectroscopic data for compound **b4**.

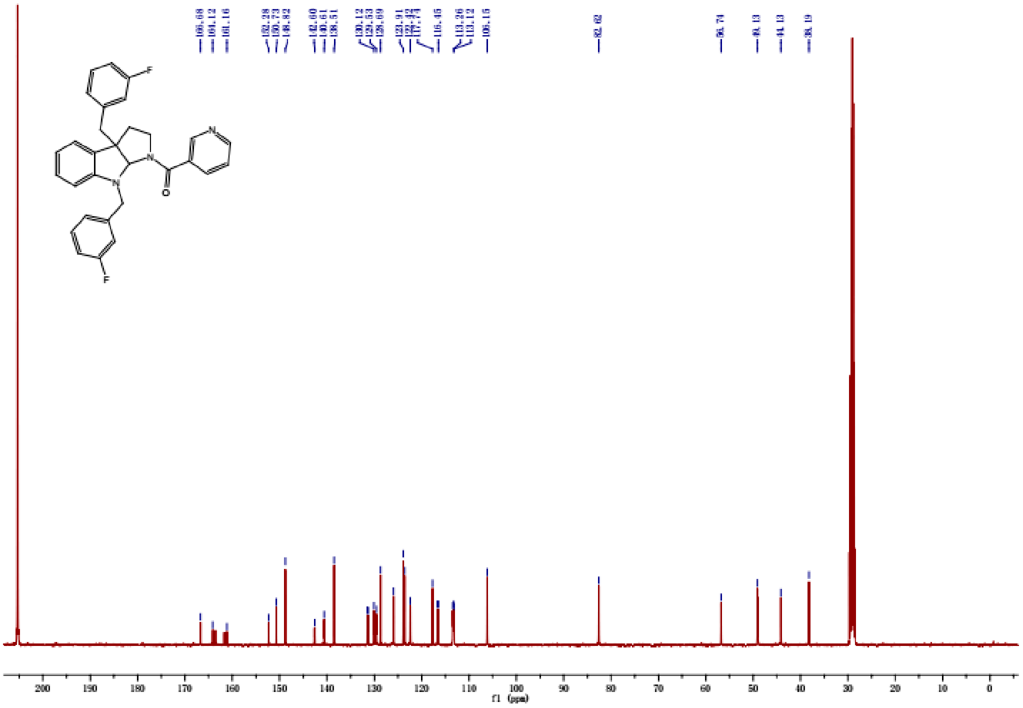

Figure S48. ^{13}C-NMR spectroscopic data for compound **b4**

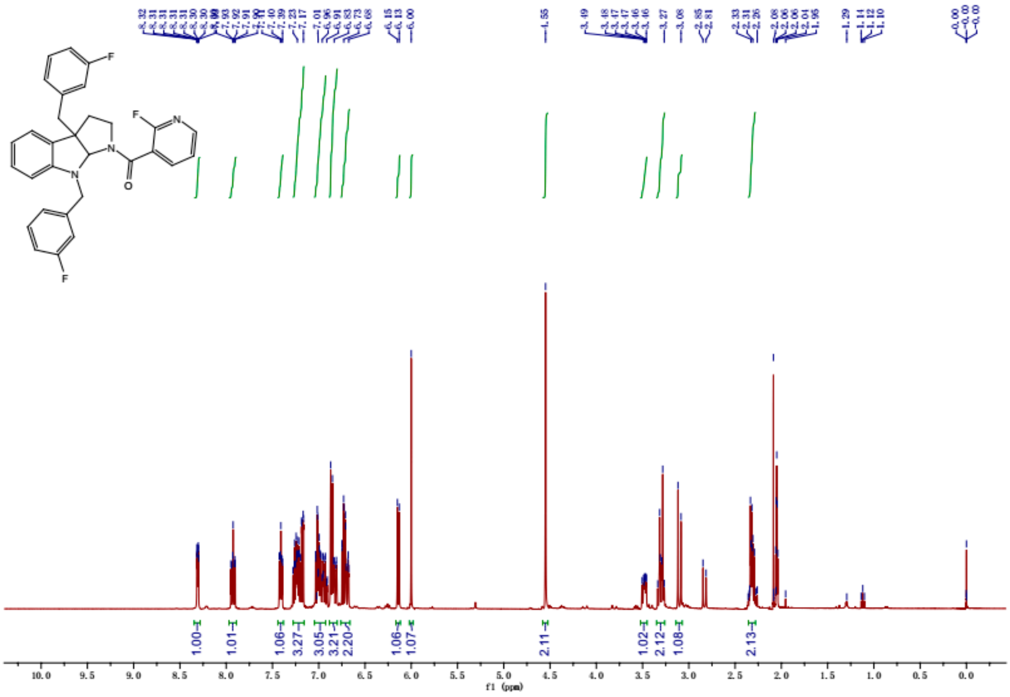

Figure S49. ^{1}H-NMR spectroscopic data for compound **b5**.

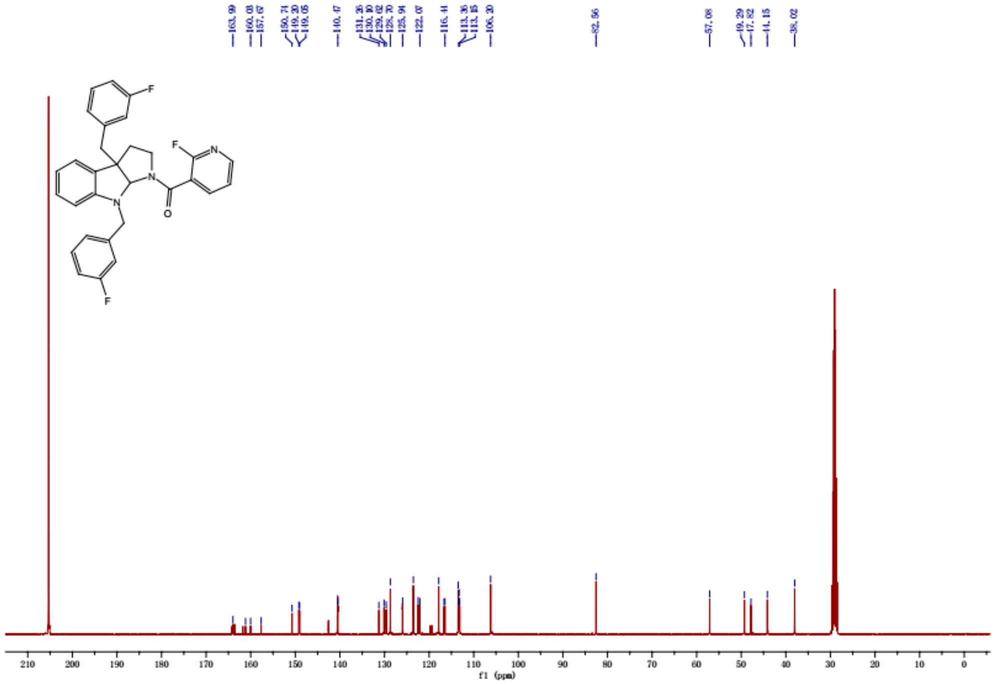

Figure S50. ^{13}C-NMR spectroscopic data for compound **b5**

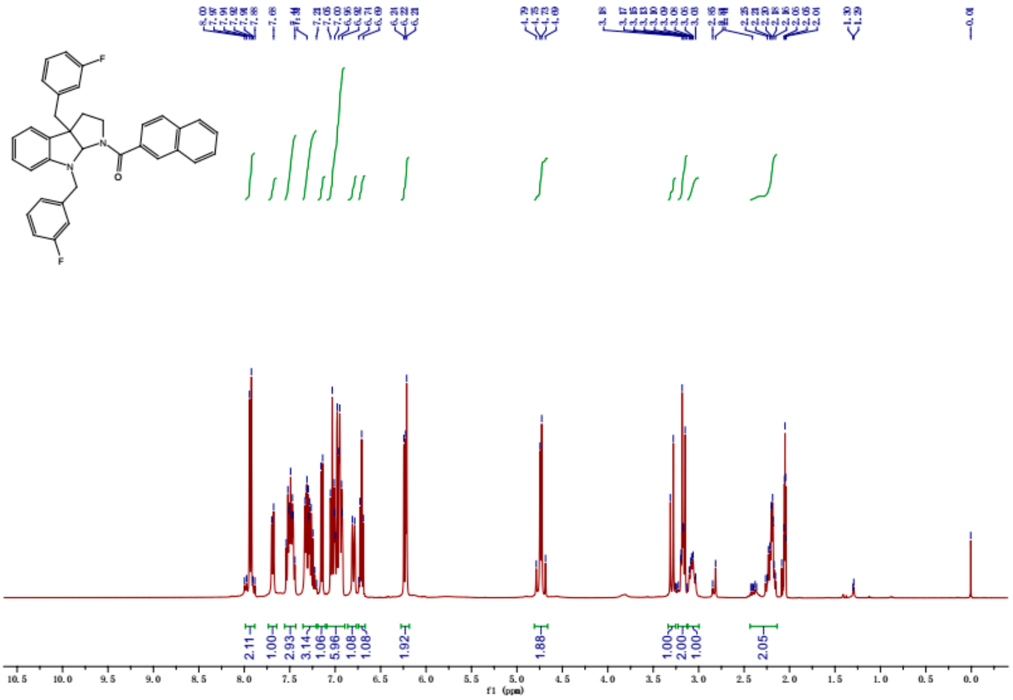

Figure S51. ^{1}H-NMR spectroscopic data for compound **b6**.

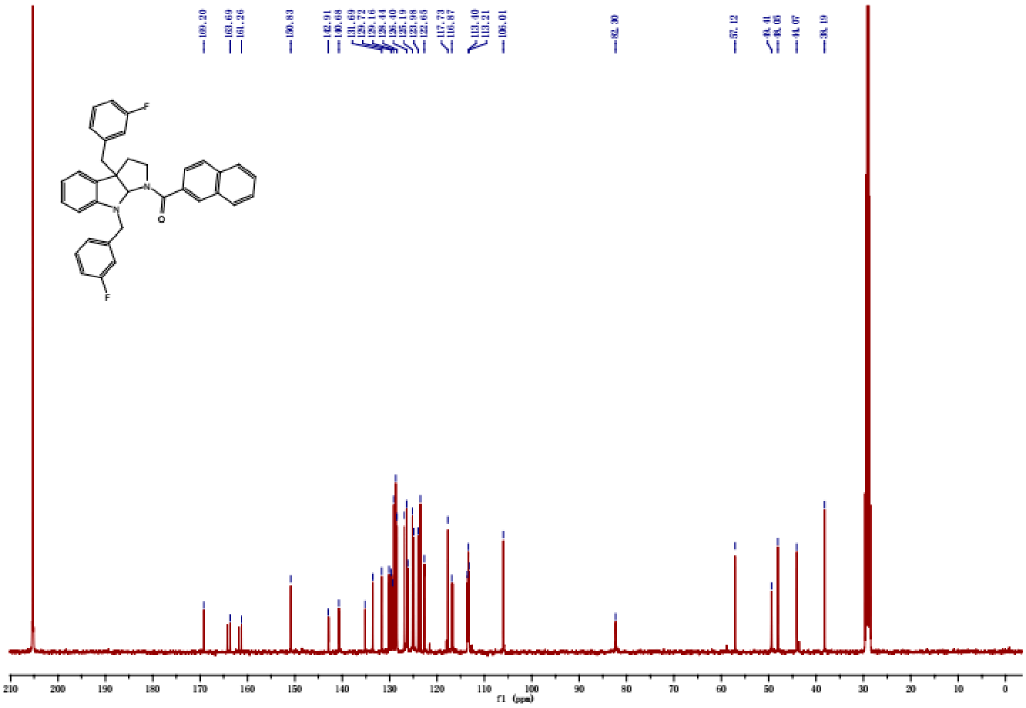

Figure S52. ^{13}C-NMR spectroscopic data for compound **b6**

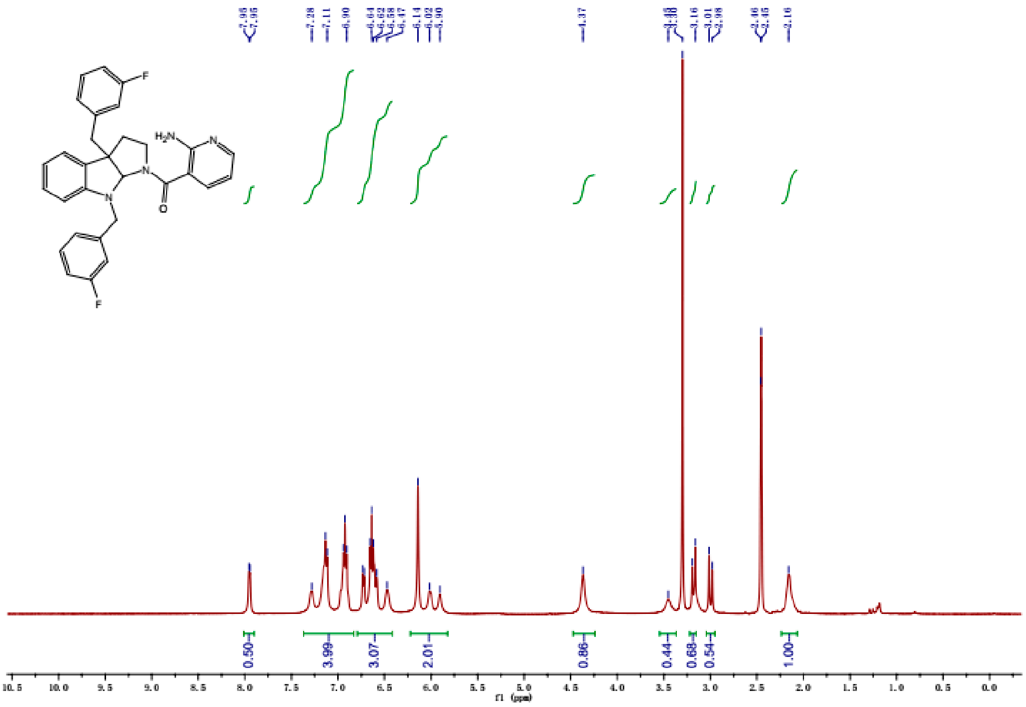

Figure S53. ^{1}H-NMR spectroscopic data for compound **b7**.

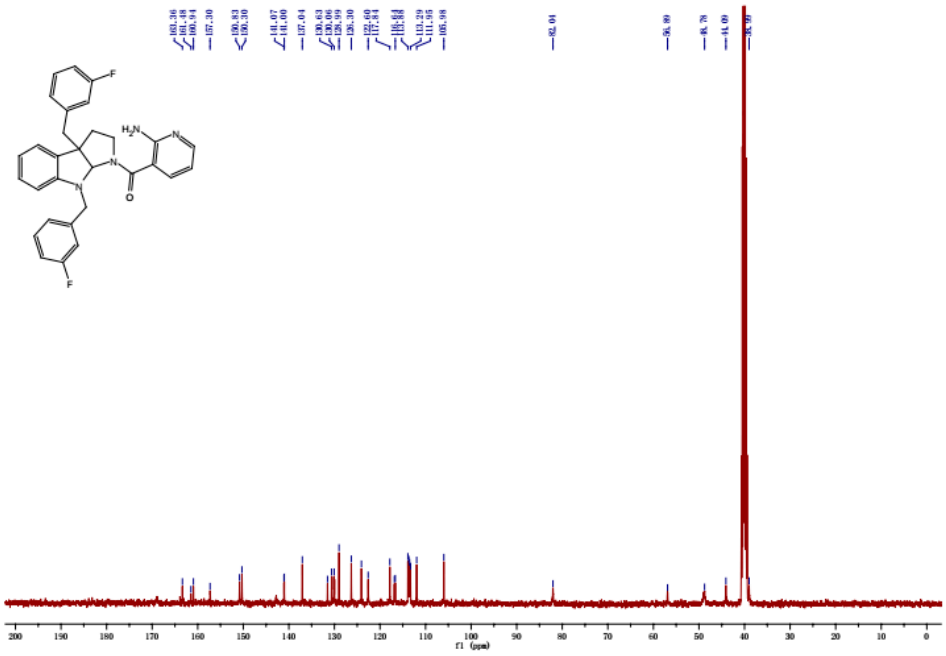

Figure S54. ^{13}C-NMR spectroscopic data for compound **b7**

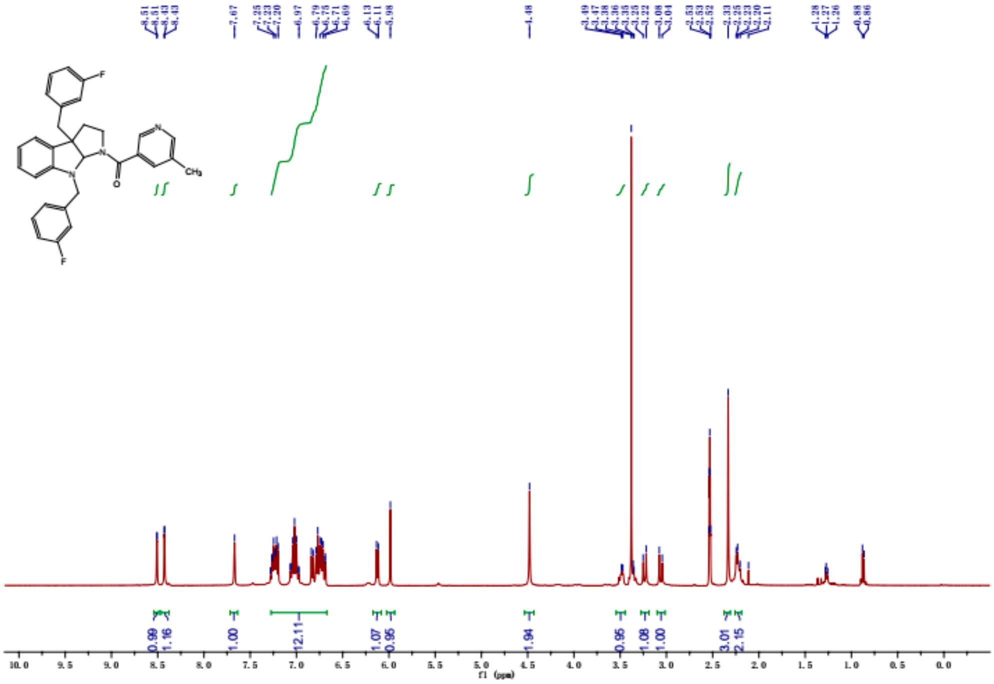

Figure S55. ^{1}H-NMR spectroscopic data for compound **b8**.

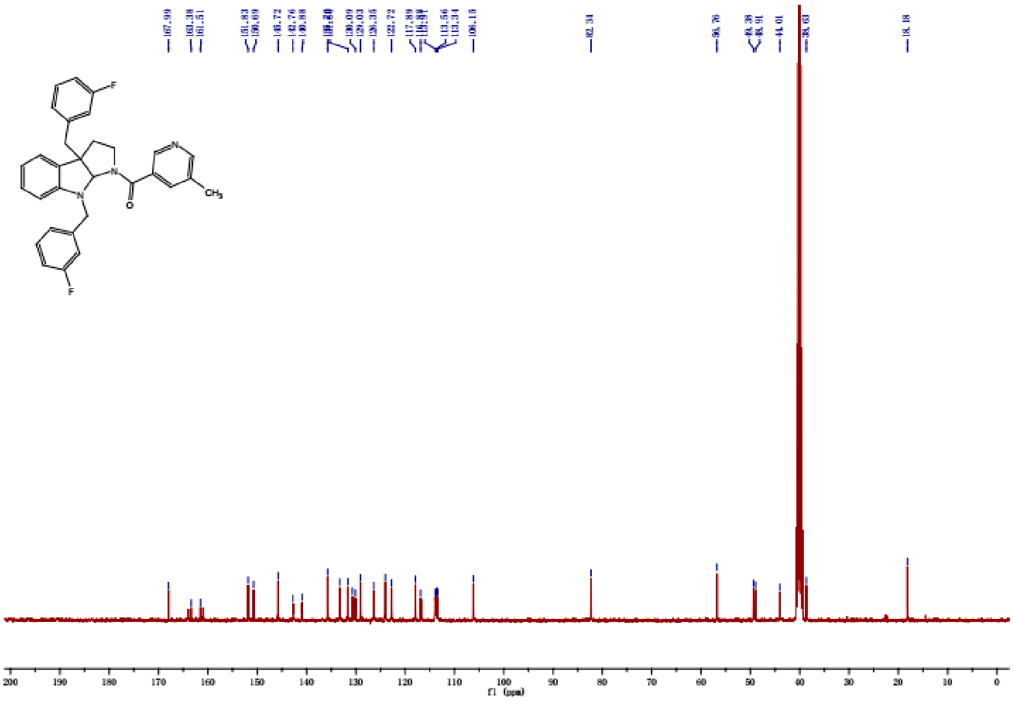

Figure S56. ^{13}C-NMR spectroscopic data for compound **b8**

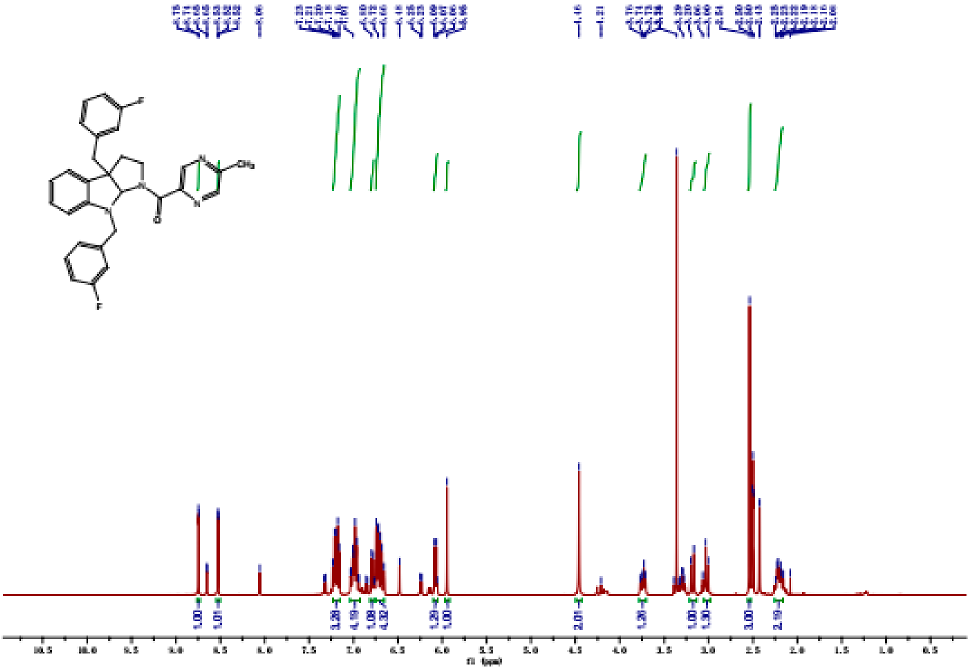

Figure S57. ^{1}H-NMR spectroscopic data for compound **b9**.

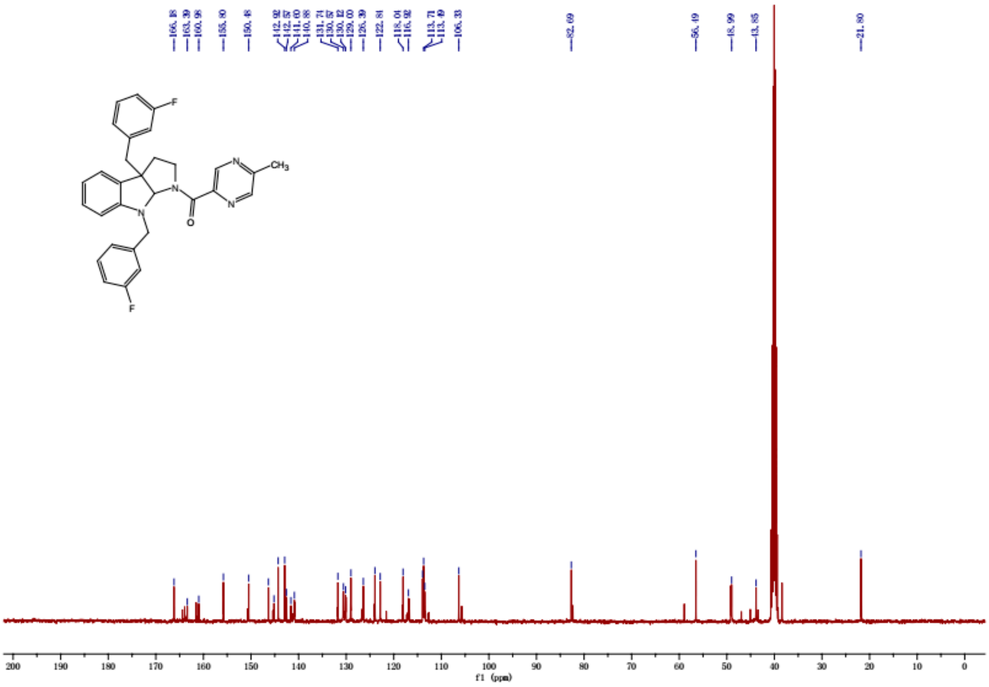

Figure S58. ^{13}C-NMR spectroscopic data for compound **b9**

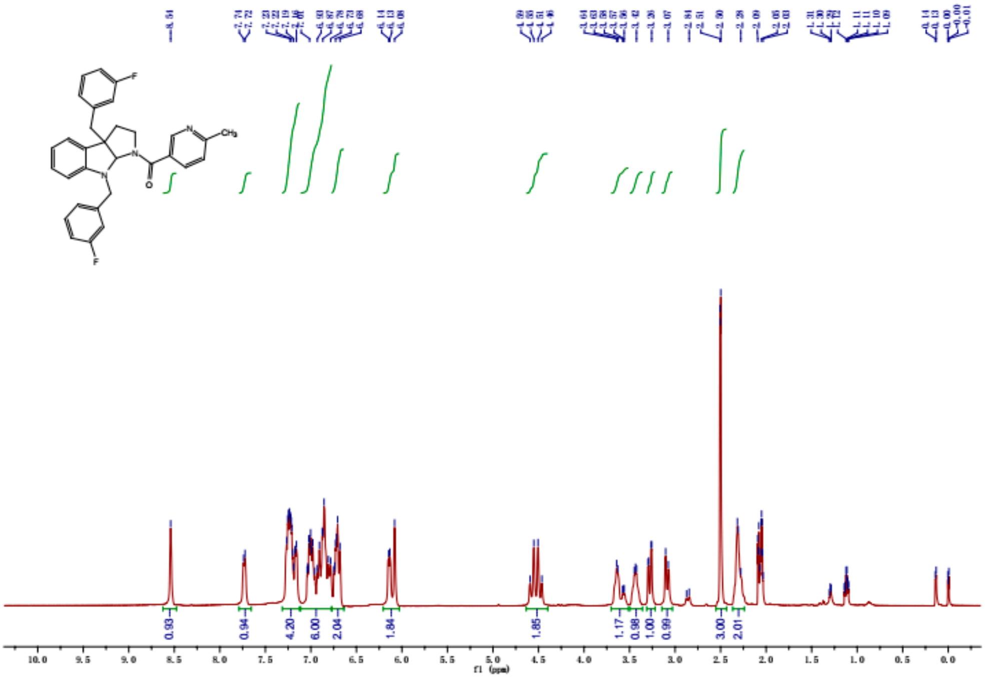

Figure S59. ^{1}H-NMR spectroscopic data for compound **b10**.

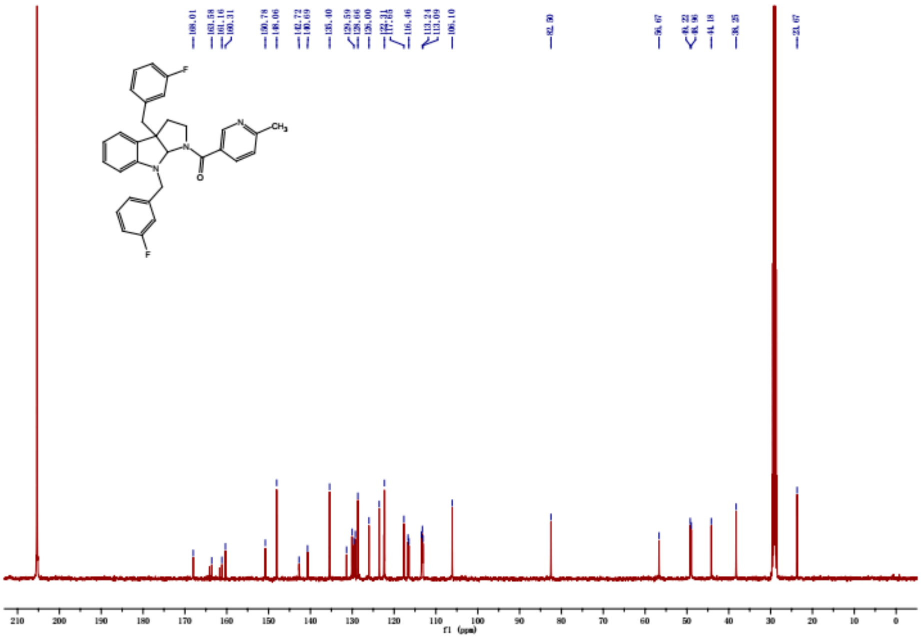

Figure S60. ^{13}C-NMR spectroscopic data for compound **b10**

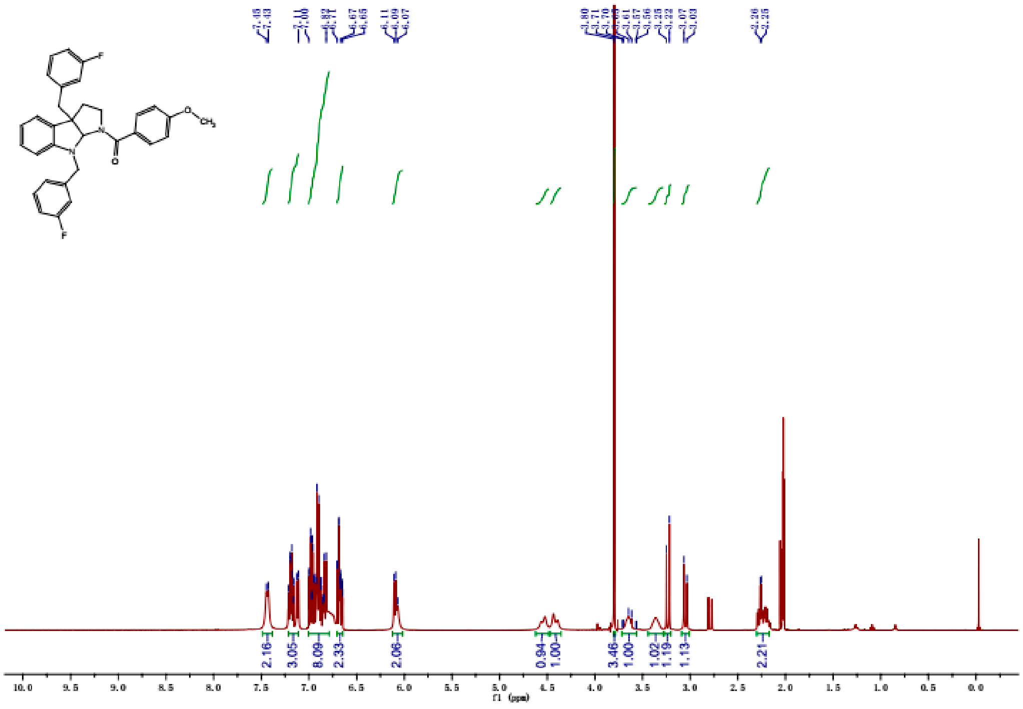

Figure S61. ^{1}H-NMR spectroscopic data for compound **b11**.

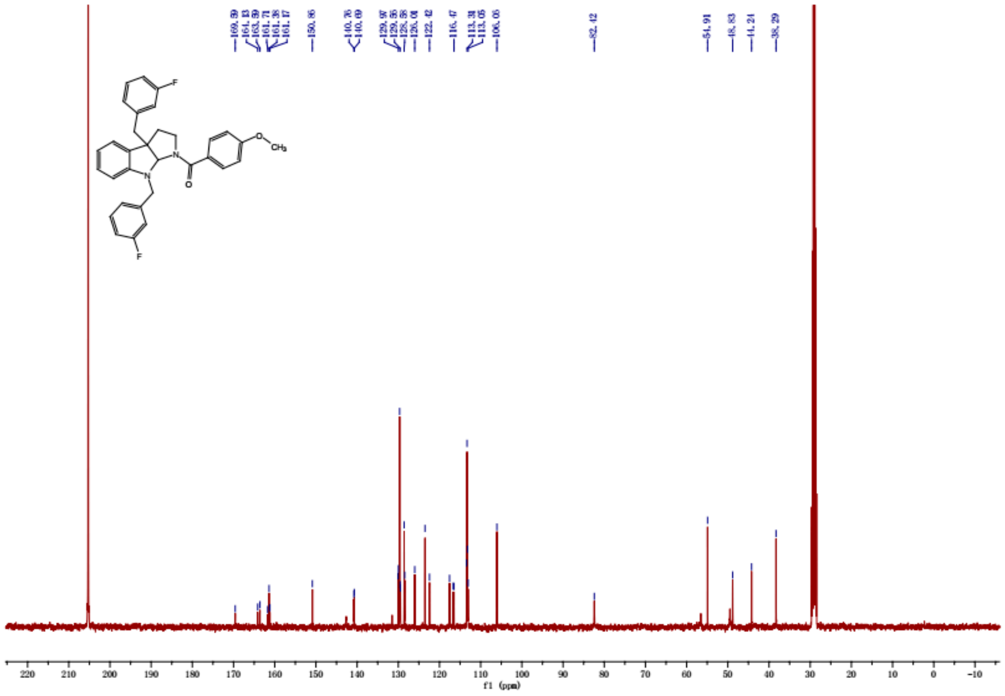

Figure S62. ^{13}C-NMR spectroscopic data for compound **b11**

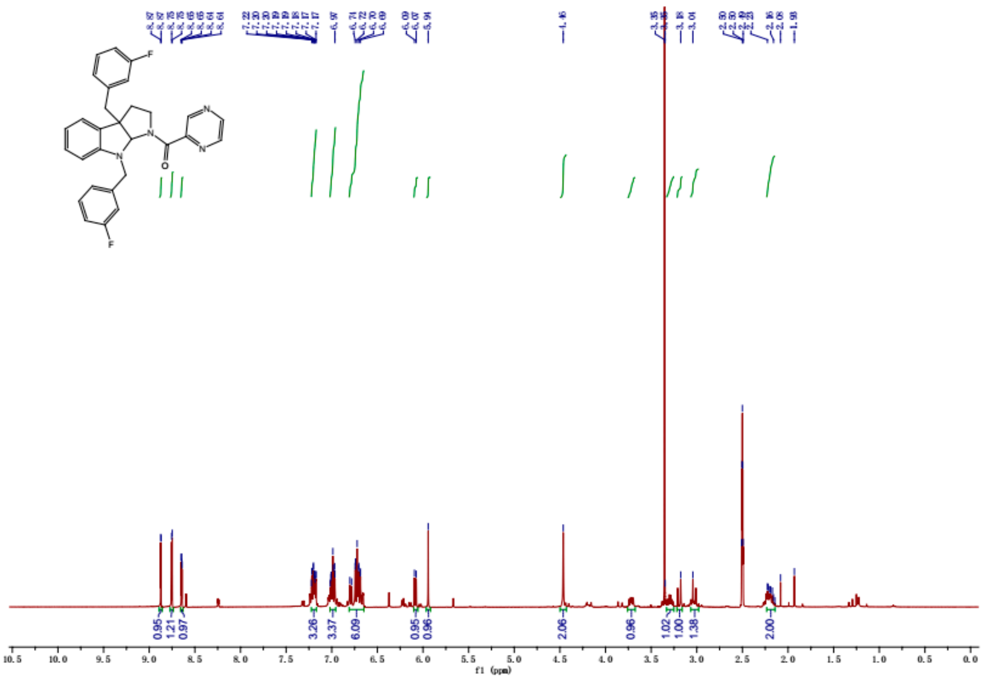

Figure S63. ^{1}H-NMR spectroscopic data for compound **b12**.

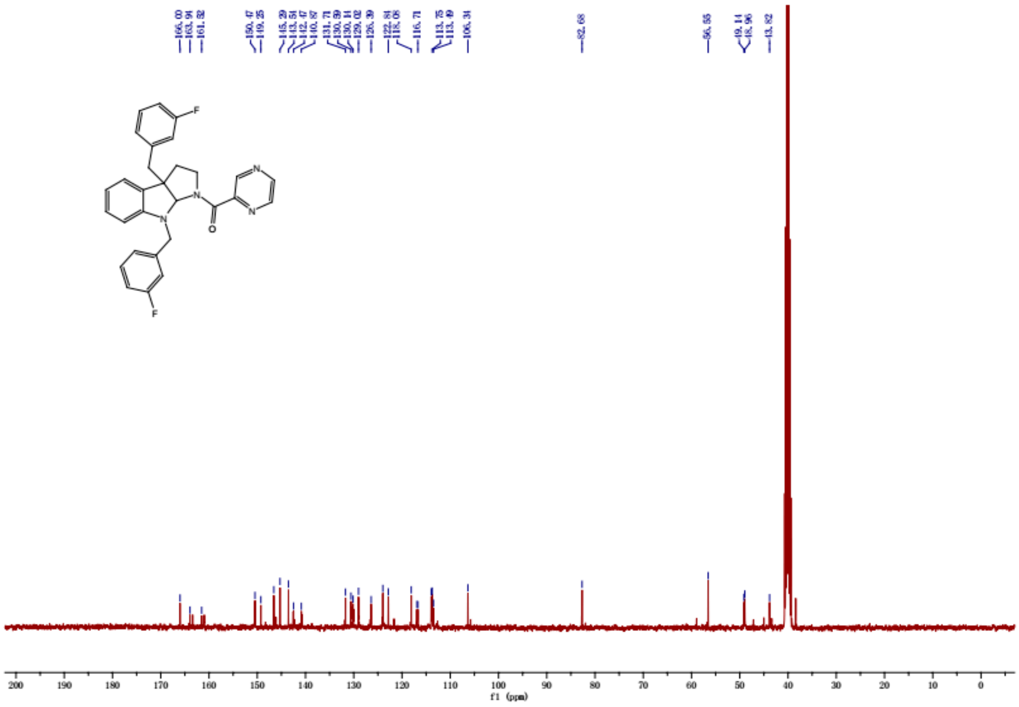

Figure S64. ^{13}C-NMR spectroscopic data for compound **b12**

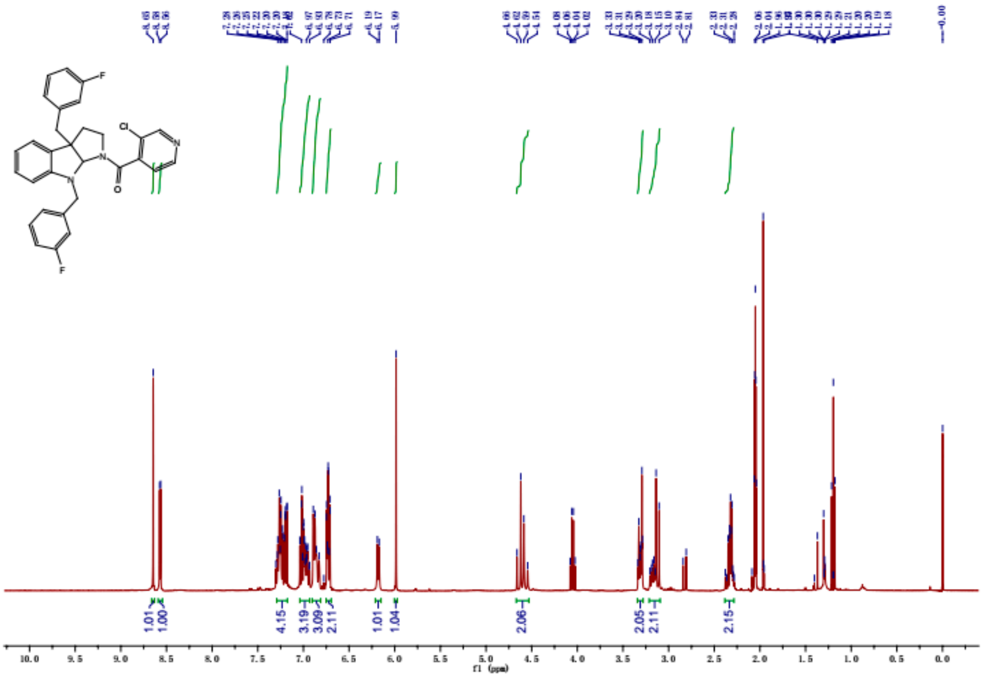

Figure S65. ^{1}H-NMR spectroscopic data for compound **b13**.

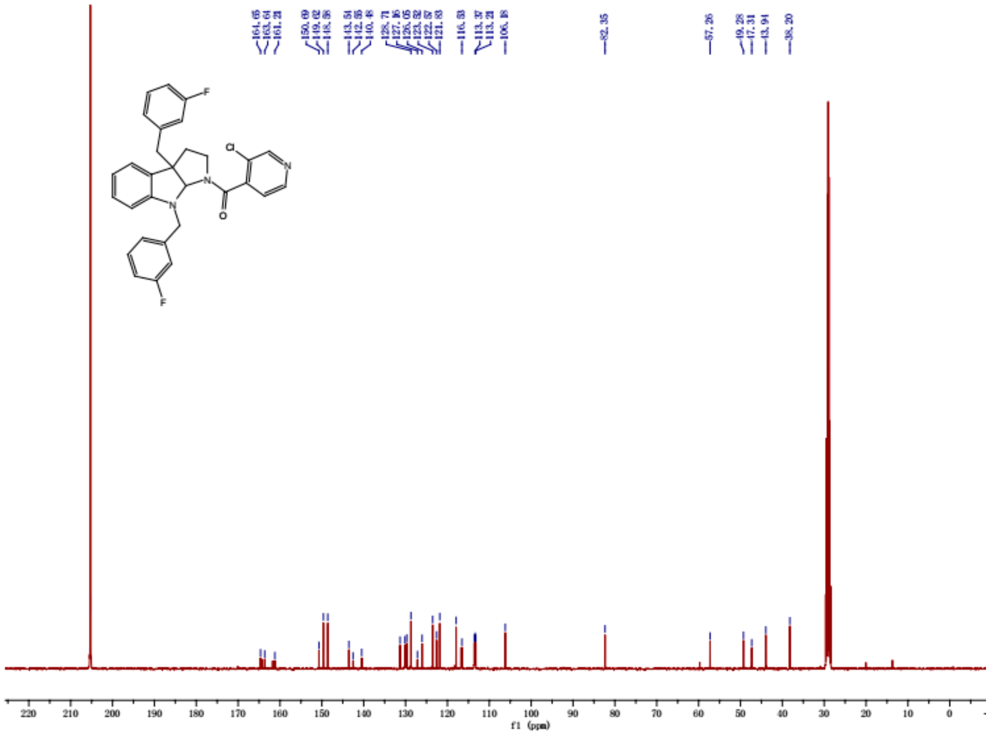

Figure S66. ^{13}C-NMR spectroscopic data for compound **b13**

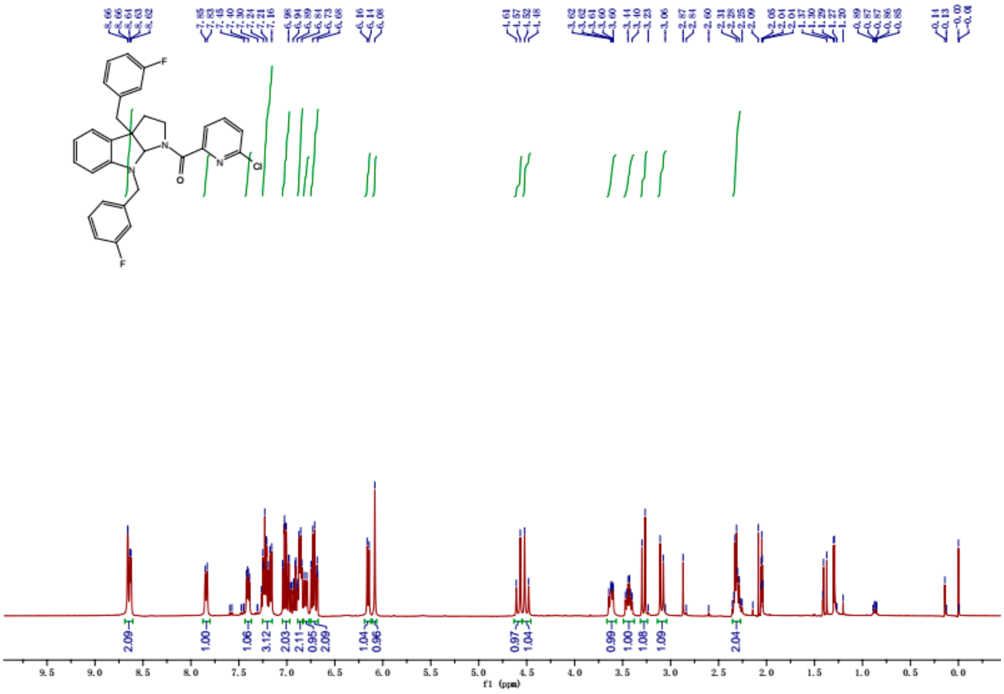

Figure S67. ^{1}H-NMR spectroscopic data for compound **b14**.

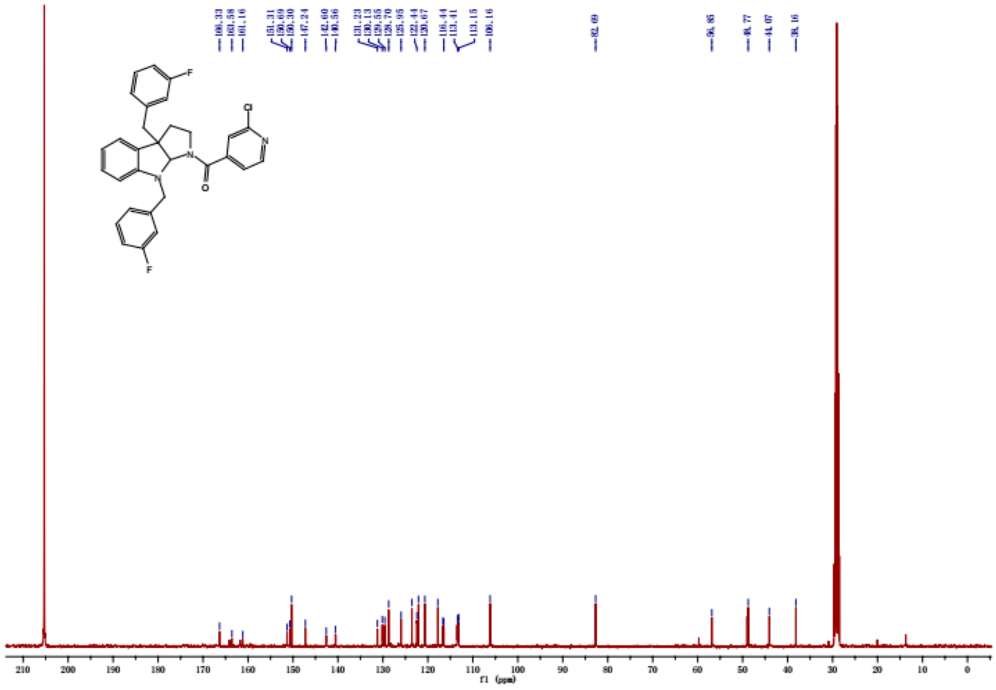

Figure S68. ^{13}C-NMR spectroscopic data for compound **b14**

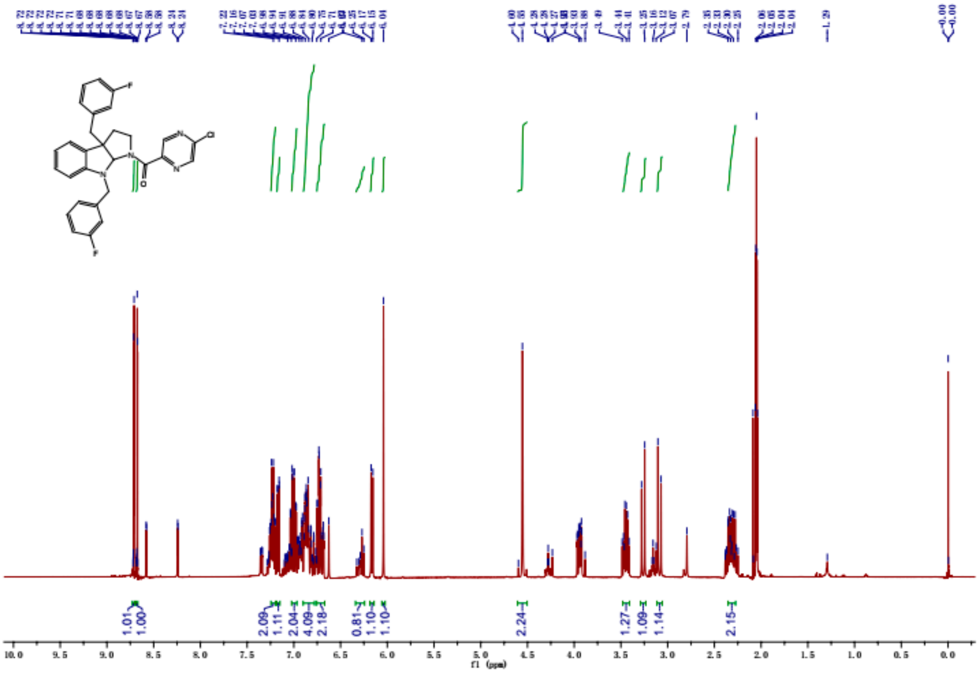

Figure S69. ^{1}H-NMR spectroscopic data for compound **b15**.

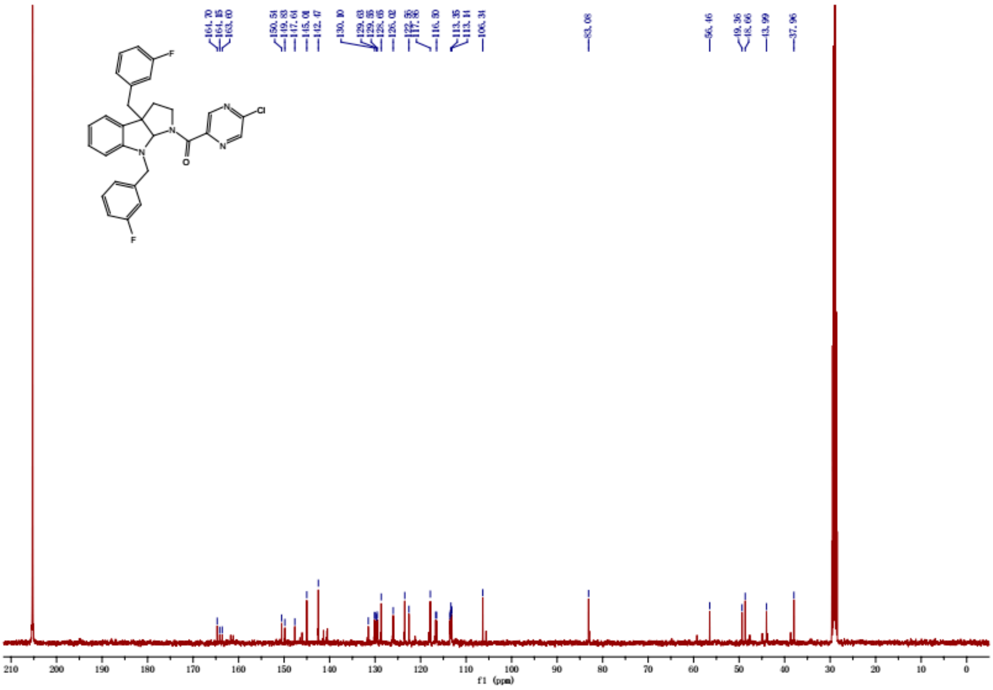

Figure S70. ^{13}C-NMR spectroscopic data for compound **b15**

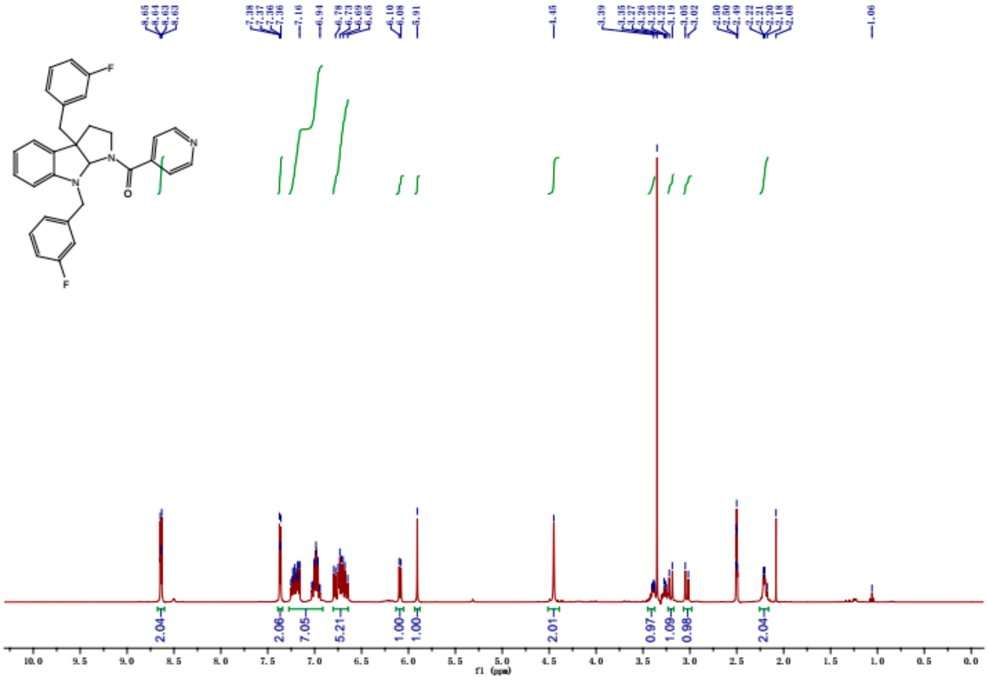

Figure S71. ^{1}H-NMR spectroscopic data for compound **b16**.

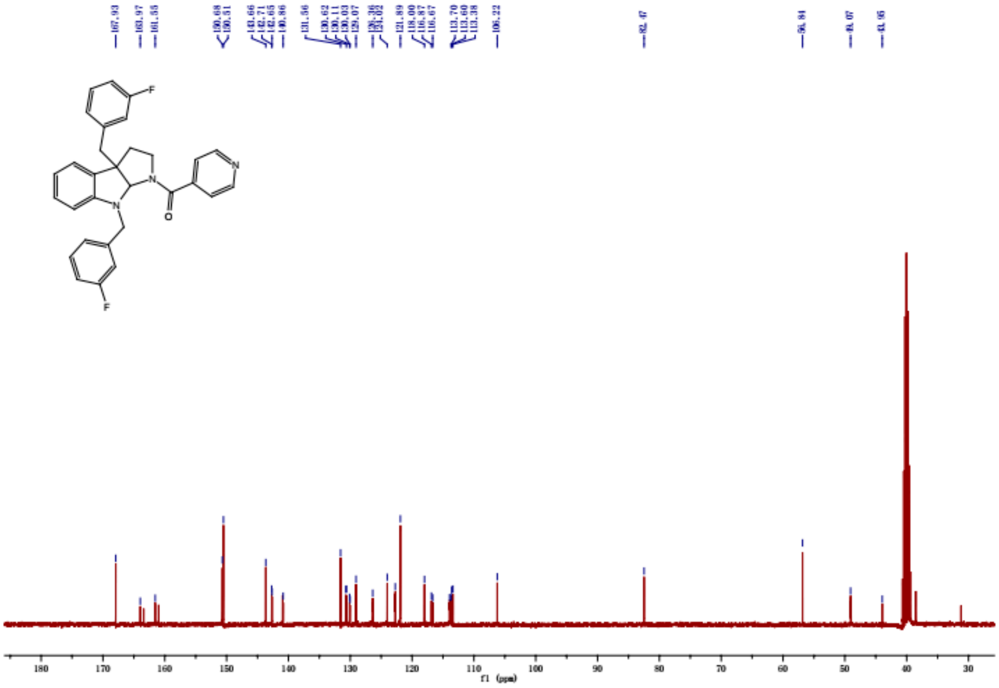

Figure S72. ^{13}C-NMR spectroscopic data for compound **b16**

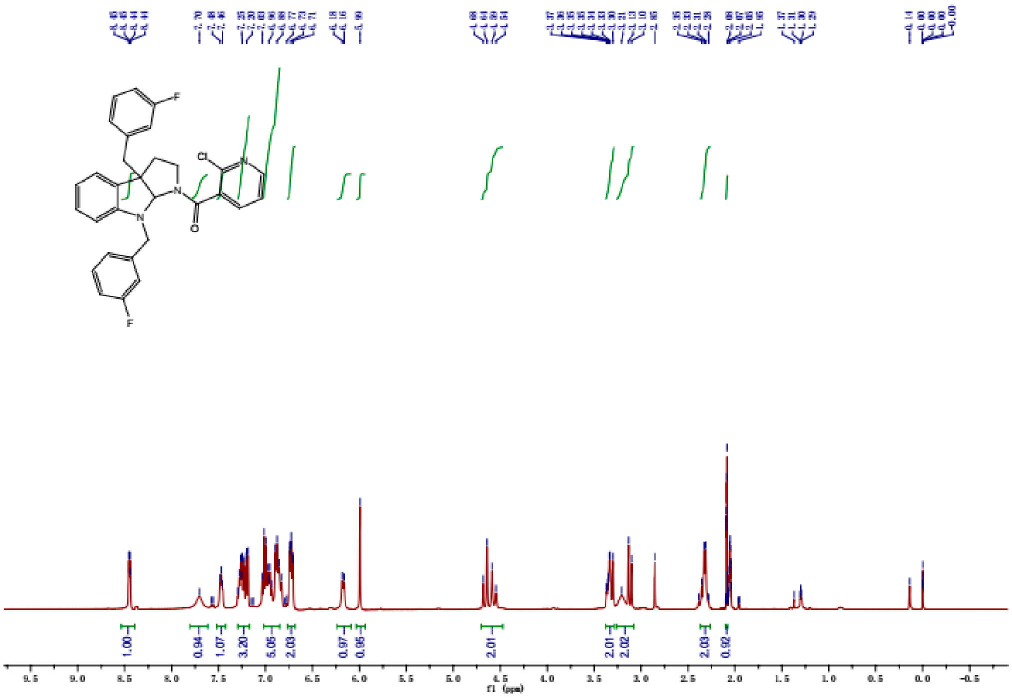

Figure S73. ^{1}H-NMR spectroscopic data for compound **b17**.

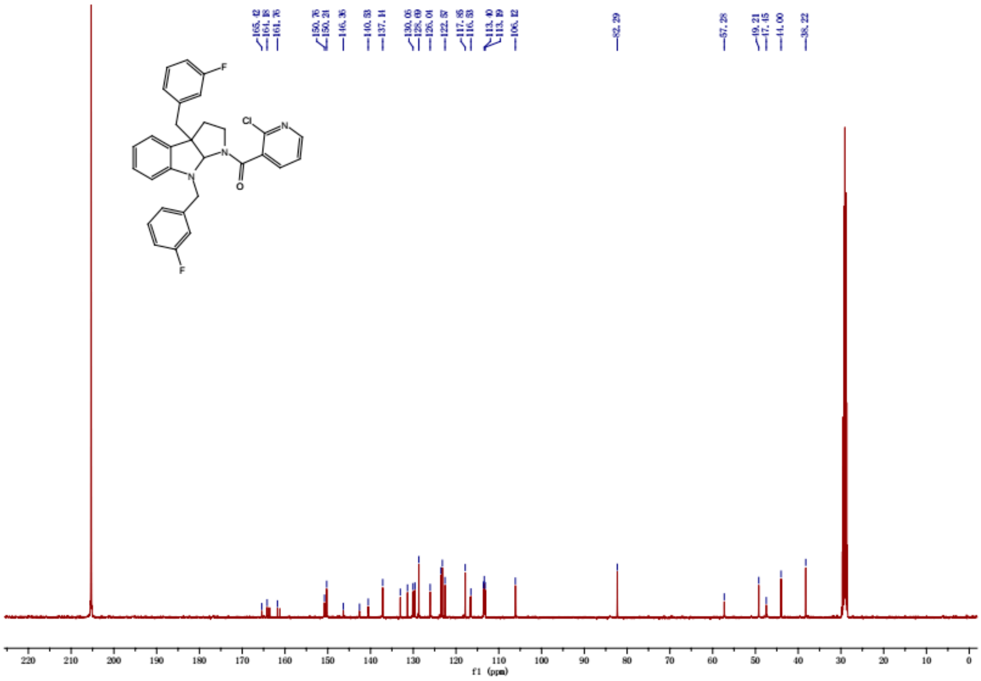

Figure S74. ^{13}C-NMR spectroscopic data for compound **b17**

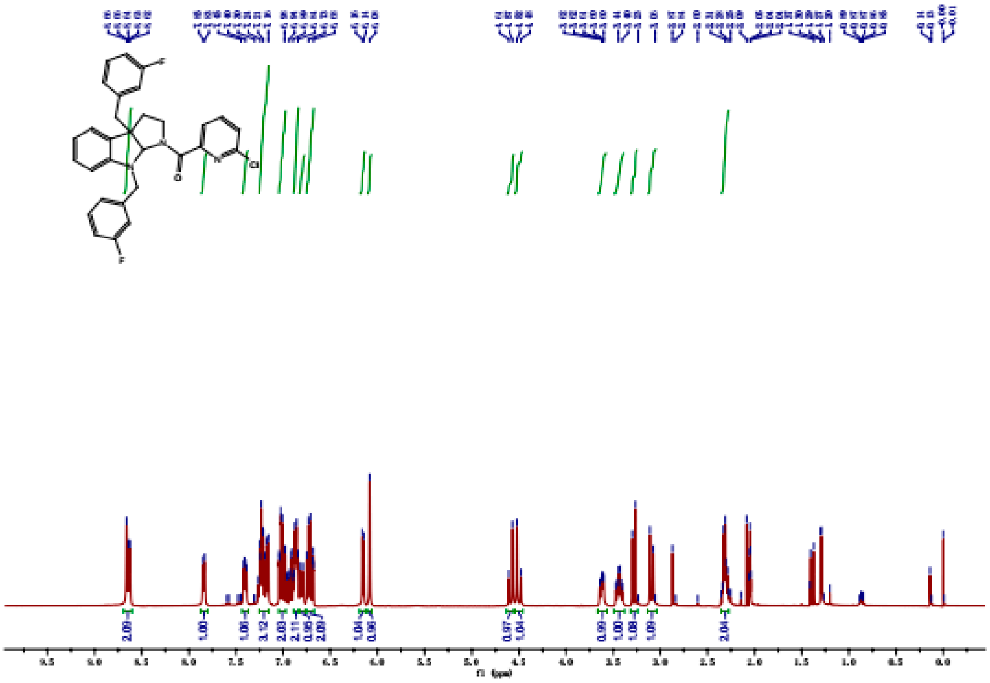

Figure S75. ^{1}H-NMR spectroscopic data for compound **b18**.

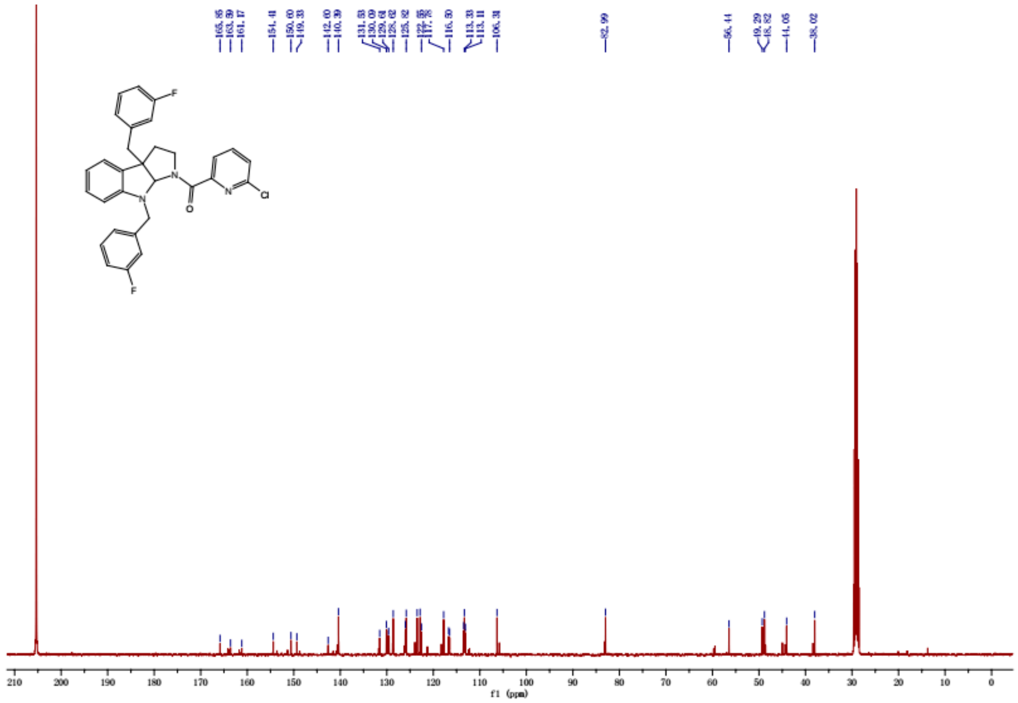

Figure S76. ^{13}C-NMR spectroscopic data for compound **b18**

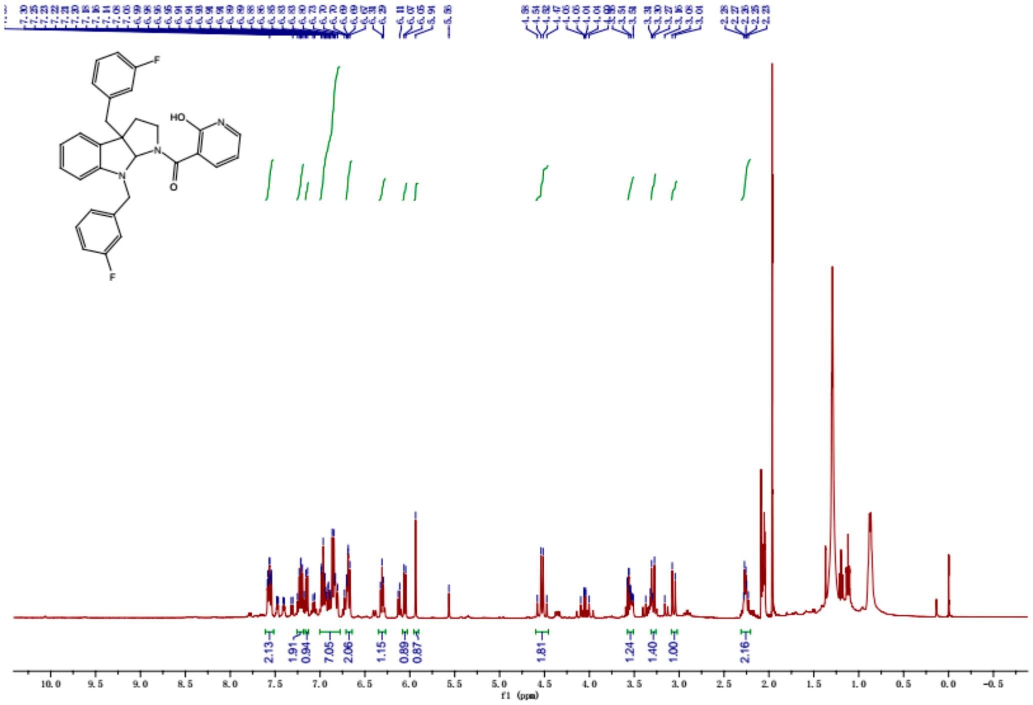

Figure S77. ^{1}H-NMR spectroscopic data for compound **b19**.

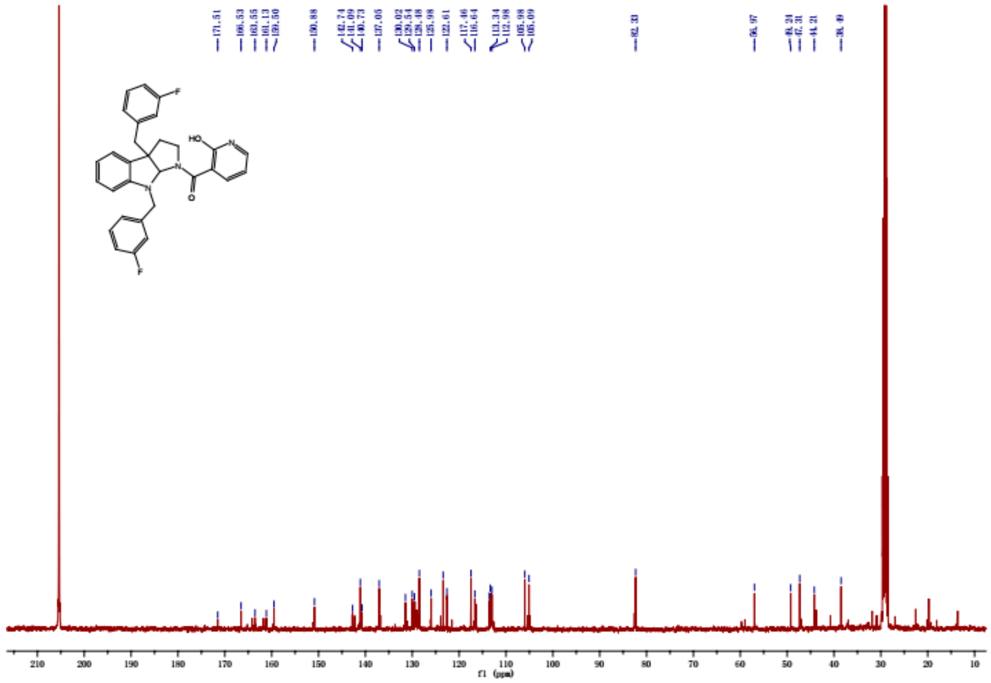

Figure S78. ^{13}C-NMR spectroscopic data for compound **b19**
